# Use of cervicovaginal fluid for the identification of biomarkers for pathologies of the female genital tract

**DOI:** 10.1186/1477-5956-8-63

**Published:** 2010-12-08

**Authors:** Geert Zegels, Geert AA Van Raemdonck, Wiebren AA Tjalma, Xaveer WM Van Ostade

**Affiliations:** 1 Laboratory of Proteinscience, Proteomics and Epigenetic Signaling, University of Antwerp, Universiteitsplein 1, 2610 Antwerp, Belgium; 2 Department of Gynecology and Gynecologic Oncology, University Hospital Antwerp, Wilrijkstraat 10, 2650 Antwerp, Belgium

## Abstract

Cervicovaginal fluid has an important function in the homeostasis and immunity of the lower female genital tract. Analysis of the cervicovaginal fluid proteome may therefore yield important information about the pathogenesis of numerous gynecological pathologies. Additionally, cervicovaginal fluid has great potential as a source of biomarkers for these conditions.

This review provides a detailed discussion about the human cervicovaginal proteome and the proteomics studies performed to characterize this biological fluid. Furthermore, infection-correlated pathological conditions of the female genital tract are discussed for which cervicovaginal fluid has been used in order to identify potential biomarkers. Recent years, numerous studies have analyzed cervicovaginal fluid samples utilizing antibody-based technologies, such as ELISA or Western blotting, to identify biomarkers for preterm birth, premature preterm rupture of membranes, bacterial vaginosis and cervical cancer. The present article will discuss the importance of proteomic technologies as alternative techniques to gain additional meaningful information about these conditions. In addition, the review focuses on recent proteomic studies on cervicovaginal fluid samples for the identification of potential biomarkers. We conclude that the use of proteomic technology for analysis of human cervicovaginal fluid samples is promising and may lead to the discovery of new biomarkers which can improve disease prevention and therapy development.

## Female genital tract physiology

The female genital tract is characterized by a unique immunologic micro-environment. It is essential for human reproduction that the immune system of the female genital tract is modulated correctly as it needs to tolerate the presence of sperm and fertilized oocytes without starting an immune reaction. Nevertheless, a strong immune system in the genital tract is very important to protect the interior organs and the fetus or embryo against the large pathogenic stress [1]. This obligation of a dual immune system, one that tolerates and one that reacts efficiently, is a biological challenge. Failure to successfully accomplish this challenge can lead to specific gynecopathological conditions which eventually may result in severe complications.

The lower female genital tract (vagina and ectocervix) is lined with a protective non-keratinized stratified squamous epithelium, whereas the higher genital tract (uterus, oviducts and ovaria) is lined with a columnar epithelium connected with tight junctions. The mucosa creates a physical barrier for invading microorganisms due to the production of a glycocalyx (i.e. a hydrophilic glycoprotein layer) which allows for hydration of the mucosa which hinders the adherence of bacteria on epithelial cells. Nevertheless, when bacteria are able to reach the mucosa and adhere to it, they will be removed by cell shedding [1-8].

The mucosa of the lower female genital tract is covered by numerous commensal bacteria which form an important protective factor. The normal vaginal bacterial flora consists predominantly of *Lactobacillus *spp. (e.g. *Lactobacillus jenensii, Lactobacillus crispatus*, *Lactobacillus iners*) but (facultative) anaerobic species such as *Gardnerella vaginalis *are also present although in much lower concentrations. Glycogen from exfoliated cells is first metabolized to glucose by epithelial cells which is then used by the vaginal flora to produce lactic acid, thereby maintaining a low vaginal pH (3.5-4.7). Also, some *Lactobacillus *spp. (e.g. *Lactobacillus crispatus, Lactobacillus rhamnosus *and *Lactobacillus acidophilus*) produce H_2_O_2 _at concentrations lethal for exterior microorganisms. Additionally, commensals compete with nonresident bacteria for available nutrients and some bacteria produce broad-spectrum antimicrobial peptides (i.e. bacteriocins). Together, these factors exert a selective antimicrobial activity which inhibits the growth of nonresident bacteria without affecting the survival of the commensal flora [1,2,7,9-11].

The presence of adaptive immunity mechanisms in the female genital tract is known, although detailed information is scarce. Langherhans (dendritic) cells, which reside in the genital mucosa, and epithelial cells are able to present antigens to T lymphocytes and thus can elicit an adaptive immune response [3,12]. Also, plasma cells are localized nearby submucosal glands and secrete, after activation, immunoglobulins (primarily IgG and secretory IgA) into the cervicovaginal fluid (CVF; see below). These immunoglobulins can recruit and activate other immune cells, hinder bacterial adherence and opsonize pathogens. Additionally, mucosal-associated lymphoreticular tissue (MALT), which consists mainly of T-lymphocytes and monocytes/macrophages, is located in the lamina propria of the cervix [2].

Another vital element of the immune system of the female genital tract is CVF. CVF is made of (i) vulvar secretions from sebaceous, sweat, Bartholins and Skene glands, (ii) plasma transudate through the vaginal wall, (iii) exfoliated cells, (iv) bacterial products, (v) cervical mucus (vi) endometrial and oviductal fluids and (vii) secretions from vaginal immune cells. The latter three are influenced by sex steroid hormones e.g. during the menstrual cycle and pregnancy [13,14]. CVF consists mainly of water and contains many different factors such as cholesterol, lipids, mucin, carbohydrates, amino acids, proteins and inorganic ions. It covers the lower female genital tract and hydrates the mucosa, creating a physical barrier for microbial invasion.

Many studies have used CVF as samples for the analysis of proteins and found correlations between different expression profiles and specific pathologies. It is therefore not surprising that during the last 5 years several comprehensive proteomics studies have been performed on CVF in order to catalogue the CVF proteome and to look for potential biomarkers.

## Cervicovaginal fluid as potential biomarker source

Over the last 10 years, the potential of using body fluid for the identification of diagnostic or prognostic biomarkers has been recognized [15]. This is also the case for CVF, where the low cost and ease of sample collection, circumvention of the risk associated with biopsies and the possibility of observing high numbers of patients using multiple samples are clear advantages of this body fluid [16-18]. Four different methods are frequently applied for the collection of CVF: 1) cervicovaginal washings (i.e. the cervicovagina is rinsed with washing buffer after which the fluid is collected), 2) cervicovaginal swabs (i.e. swabs or brushes are applied to the mucosal wall and rotated to collect the CVF), 3) cervicovaginal wicks (i.e. wicks such as tampons, strips or sponges are inserted in the female genital tract which absorb cervicovaginal secretions) and 4) diaphragm-like devices (i.e. cups placed over the cervix which collect cervical fluid). Few studies have been performed on the reliability and validity of the CVF collection methods. Snowhite *et al*. [19] compared the wicking method with cervicovaginal washings and found that cervicovaginal washings are the superior sample collection method for cytokine assays in terms of reproducibility. Another research group demonstrated that cervicovaginal washings are better samples as compared to swabs for assessment of HIV1 RNA in cervicovaginal secretions [20]. Lastly, collection of samples using cups has the advantage that ready-to-use samples are obtained. Because the secretions are not absorbed (cfr. swabs and wicks), no sample recovery steps need to be undertaken and protein loss is minimized. In addition, the cervical secretions are not diluted in a washing buffer during sample collection (cfr. cervicovaginal washings). However, because the cups are placed over the cervix, this sample collection method has the disadvantage that only cervical secretions and no vaginal secretions are collected.

These examples indicate that the sample collection method may influence results. Therefore, well defined and standardized sampling methods may help avoiding misinterpretation of results and decrease the technical variation of the experimental setup.

So far, plasma/serum is the most analyzed body fluid compared to other body fluids such as cerebrospinal fluid, urine, saliva, bronchoalveolar lavage fluid, nipple aspirate fluid, tear fluid and amniotic fluid [18]. A great advantage of plasma/serum is that these samples contain a plethora of potential biomarkers since this body fluid is in contact with nearly all organ systems. Nevertheless, some important disadvantages are inherent to the use of plasma/serum. Due to the presence of plasma/serum in the entire body, biomarkers often lack discriminatory power for a particular organ system and thus are not specific enough. Also, since the volume of plasma/serum is substantial, potential biomarkers are strongly diluted, resulting in lowered sensitivity. Moreover, it is well known that serum and plasma protein concentrations have a large dynamic range which often implies extensive sample preparation and separation steps. All together, these disadvantages result in a large inter- and intravariability of plasma/serum samples [16].

Other body fluids such as cerebrospinal fluid, urine or CVF are less subjected to these restraints. For instance, it can be expected that biomarkers found in CVF, being a "proximal" fluid, show more specificity and sensitivity for gynecopathological conditions as compared to blood [21]. This is due to 1) the relatively small volume/organ ratio of CVF, thereby increasing the possibility that potential biomarkers are less diluted, thus increasing their sensitivity and 2) CVF is a body fluid that is specific for the female genital tract and hence the CVF proteome will be related to conditions of these organs [16,18,21,22]. CVF is therefore an interesting body fluid to use for the development of biomarkers for gynecological pathologies. One must keep in mind however that many factors may significantly influence the protein composition of the CVF. For example, secretions of the genital tract are notably influenced by varying levels and ratios of estrogens and progesterone during the menstrual cycle [23] resulting in variable amounts of protein yield (for instance, we measured between 0.58 mg and 13.37 mg in cervicovaginal washings). Therefore it is practically impossible to quantify the normal amount of total protein content in healthy CVF. Although one can expect some changes in the amount of protein content for pathological conditions, these are often unnoticeable due to the large normal fluctuations. Because of this variation, it is often difficult to distinguish real biomarkers from natural occurring biophysiological variations [16,24]. For example, analysis of microbicide induced cytokine deregulation is often hindered by the menstrual cycle [4,25-27], pathological conditions, sexual intercourse [28], age [29], and the use of contraceptives [30-33] or hormonal substitution therapies. If one disregards these effects, they will inevitably lead to biased or confounded results. The use of experimental designs of high-quality and well determined and clinically validated criteria are required to detect real product induced cytokine changes from background levels [34].

The use of body fluid as biomarker source implies the application of proteomic methodologies, because body fluids do not contain a corresponding genome or transcriptome and therefore gene expression cannot be analyzed [17]. Most studies on the identification of potential biomarkers are restricted to the use of antibody dependent techniques (e.g. ELISA or Western blotting). Thus far, only a limited number of large proteomic studies have been performed on CVF and the majority of these studies focused on the characterization of the CVF proteome. Only during the last few years quantitative methods were used to detect differences in protein profiles between particular conditions [14,22,35-45].

## Proteomics techniques for the characterization of the human cervicovaginal proteome

Generally, a proteomic biomarker study can be divided in several steps which are critical to obtain clinically and statistically significant results. (i) The characterization and analysis of the proteome, which also includes analysis of variation, is a first essential stage followed by (ii) differential analysis of the proteome, including statistical analysis to identify potential biomarkers (discovery phase). In a next phase (iii) the potential markers need to be validated on other independent cohorts (validation phase) after which (iv) the implementation possibilities of the validated markers in a clinical environment can be investigated [16,18]. Only the last 5 years, studies started to investigate the human CVF proteome to characterize the protein composition and to detect protein expression differences between specific patient conditions (table 1) using comprehensive antibody-independent proteomics techniques.

**Table 1:** Overview of primarily qualitative proteomic studies performed on human CVF and cervical mucus.

*Study*	*Samples*	*Separation method*	*MS method*
Venkataraman *et al*., 2005[38]	Undiluted CVF collected in cup from healthy women (postmenarcheal, pre-menopausal)	2D-PAGE (1D: AU-PAGE; 2D: Tricine- SDS-PAGE)	MALDI-TOF-TOF

Di Quinzio *et al*., 2007[40]	Swabs from pregnant women (37 weeks gestation)	2D-PAGE (1^st ^D: IEF; 2^nd ^D SDS-PAGE) followed by RP-LC	MALDI-TOF or ESI-linear IT

Dasari *et al*., 2007[42]	Swabs from pregnant women (18,5 weeks gestation as mean)	1D-SDS-PAGE followed by offline 2D(SCX/RP)-LC	ESI-Q-TOF

Tang *et al*., 2007[45]	Washings from clinically normal women; 7 washings from women infected with *Candida *spp.	2D-PAGE (1^st ^D: IEF; 2^nd ^D SDS-PAGE)	MALDI-TOF-TOF

Shaw *et al*., 2007[44]	Gauze from healthy women	1D-SDS-PAGE or SCX-LC both followed by RP-LC	ESI-linear IT

Pereira *et al*., 2007[43]	Swabs from pregnant women (15.8-35.9 weeks gestation)	2D-DIGE or MudPIT(SCX/RP)-LC	ESI-Q-TOF

Andersch-Björkman *et al*., 2007[36]	Cervical aspiration using syringe from healthy women	1D-PAGE or SDS-agarose electrophoresis followed by RP-LC	ESI-FT-ICR

Klein *et al*., 2008[14]	Swabs from pregnant women (30.5 weeks gestation as mean)	RP-LC	ESI-IT

Zegels *et al*., 2009[22]	Washings from HPV-infected women	Ultrafiltration or C_4_-LC protein fractionation/C_18_-LC peptide separation	MALDI-TOF-TOF

Panicker *et al*., 2010[37]	Cervical mucus from healthy women obtained with sponges	2D-PAGE (1^st ^D: IEF; 2^nd ^D SDS-PAGE) or 1D-PAGE followed by RP-LC	ESI-Q-TOF

For each study the following information is presented: 1) the nature of the samples which were used in the study (sample collection method and patient physiology), 2) the methods used to separate proteins and peptides and 3) MS method used for analysis.

Venkataraman *et al*. [38] published one of the first human CVF proteomics papers and demonstrated that human CVF may play a critical role in the host defense against HIV. The authors showed that CVF contained intrinsic anti-HIV activity from which the greater part was found in the cationic protein/peptide fraction. This fraction was analyzed using two-dimensional (native acid urea-PAGE followed by Tricine SDS-page) gel electrophoresis after which spots were identified using MALDI-TOF/TOF tandem mass spectrometry. The authors identified 18 polypeptides including known antimicrobial peptides/proteins such as calgranulin A/B, lysozyme, human neutrophil peptide (HNP) 1, cathepsin G and several histones [2]. However, none of these cationic polypeptides alone had potential anti-HIV activity. In addition, cationic fraction-depleted CVF did not contain anti-HIV activity but this activity was completely restored after adding the isolated cationic fraction. This indicates that the anti-HIV activity is almost entirely dependent on the CVF cationic fraction and probably is the result of a synergistic combination of different molecules. Also, it can be expected that, apart from the 18 proteins identified in this study, the cationic fraction from CVF is constituted of other proteins and peptides (such as elafin, see later) which could also play a role in the anti-HIV activity from CVF.

Di Quinzio *et al*. [40] used full human CVF instead of a particular fraction for their proteomics analysis. Cervicovaginal swabs from five pregnant women were collected and separated using 2D (isoelectric focusing (IEF) followed by SDS-PAGE) gel electrophoresis. Subsequently, protein spots common to all five gels were tryptically digested and identified using MALDI-TOF mass spectrometry. Alternatively, the samples were first separated using capillary reversed phase (RP)-HPLC followed by electrospray ion trap tandem mass spectrometry. The authors identified 15 different proteins common to the five samples. These proteins function in different areas such as blood transport, structural integrity, fatty acid metabolism, calcium binding, inflammation, proteinase inhibition and oxidative stress defense [40]. The group further developed their proteomics platform to allow the analysis of differences in the CVF proteome caused by normal labour and pregnancy. In a first study, the authors compared the 12-29kDa CVF proteome fraction from pregnant women at 26-30 days before labour onset, 1-3 days before onset and during natural term labour. They identified seven different proteins (cystatin-A, interleukin-1 receptor antagonist, glutathione S-transferase P, peroxiredoxin-2, thioredoxin, copper-zinc superoxide dismutase, and epidermal fatty-acid binding protein) [39]. In a follow-up study, interleukin-1 receptor antagonist (IL-1RA), was validated and shown to decrease in concentration during the course of the pregnancy. Also, the concentrations of IL-1RA were 6-fold lower in case of preterm premature rupture of membranes (see below), which indicates that IL-1RA functions in the remodeling of the fetal membranes [46]. Furthermore, the group used the same techniques to analyze the 25-45 kDa human CVF proteome fraction and compared expression profiles between pregnant women at 14-17 days, 7-10 days and 0-3 days before natural labour and during labour. The authors found temporal changes in the expression of five proteins (serpin B3, serpin B1, annexin A3, collagen α2 type IV and albumin) which correlate with natural occurring term labour [47]. Recently, the same research group showed that 1) expression of α-enolase was significantly increased during spontaneous labour at term [48] and 2) parturition results in increased oxidative stress. This last phenomenon was reflected in the CVF since the total antioxidant capacity of CVF decreased drastically during labour, largely due to a decline in copper zinc superoxide dismutase and thioredoxin-4 CVF concentrations. The combination of total antioxidant capacity of CVF and copper zinc superoxide dismutase concentration showed high specificity and sensitivity for the prediction of labour within three days [49]. These studies are good examples of how proteomics may yield important information about complex biological phenomena such as pregnancy.

Although the studies from Venkataraman *et al*. [38] and Di Quinzio *et al*. [40] were among the first to use proteomics techniques to mine the CVF proteome, their analyses were restricted to analysis of CVF proteome subfractions. In contrast, Dasari *et al*. [42] performed the first large comprehensive analysis of the CVF proteome. The authors used 2D (strong cation exchange (SCX) followed by RP)-HPLC or SDS-PAGE followed by RP-HPLC as sample separation methods. CVF proteins or peptides were identified using ESI-Q/TOF tandem mass spectrometry combined with several search engines. The study primarily aimed to identify CVF proteins and 150 different proteins were detected of which the majority functioned either as immune and defense-related or as metabolic proteins. The authors also compared serum and amniotic fluid protein sets with the proteins identified in the study. 77 proteins were unique to CVF, whereas 56 proteins were also found in serum and 17 in amniotic fluid. Additionally, they found that there is a significant degree of complementarity between the experimental setups as well as between the different search engines (Sequest, X!Tandem and OpenSea) used for the identification of CVF proteins. For example, only 38% of the spectra were identified by all three search engines, indicating that implementation of different search algorithms can result in a significant increase in number of identifications. Finally, the authors emphasized the need for biological and technical replicates in order to identify as many proteins as possible from the CVF proteome [42].

The same research group published the first comparative quantitative proteomics study on CVF. Pereira *et al*. [43] used 2D (IEF followed by SDS-PAGE) differential gel electrophoresis (2D-DIGE) to analyze differences in CVF protein expression levels between women with preterm-labour, spontaneous preterm birth patients and controls. Alternatively, they performed also 2D (SCX followed by RP) HPLC followed by ESI-Q/TOF to identify present proteins and to quantify protein expression semiquantitatively using spectral counting. During these experiments, the authors identified 205 proteins. Of those, 28 (2D-LC-MS/MS analysis) and 17 (2D-DIGE) proteins showed statistically significant changes between preterm labour patients, patients with spontaneous preterm birth and controls which can be considered as potential biomarkers [43]. This study is further discussed in more detail below.

Tang *et al*. [45] also performed a characterization of the CVF proteome using 2D (IEF followed by SDS-PAGE) gel electrophoresis and MALDI-TOF/TOF analysis for the identification of CVF proteins. 59 different proteins were thus identified. The authors showed the presence of polymorphonuclear cells (e.g. neutrophils and eosinophils) in CVF and demonstrated that these immune cells could be activated and secreted proteins in the lower genital tract. Additionally, they showed that CVF contains a large fraction (47%) of proteins which are also found in plasma, which could be indicative for the permeability status of genital tract mucosa. Since the permeability is often increased in case of cervicovaginal infection, these plasma proteins could eventually be used together with secreted proteins from activated neutrophils and eosinophils (e.g. lactoferrin, myeloperoxidase, human neutrophil lipocalin and eosinophilic cationic protein) as markers for cervicovaginal inflammation [45].

The proteomics study which resulted in the largest set of protein identifications was performed by Shaw *et al*. [44]. The authors were able to identify 685 proteins from human CVF by means of 2D (SCX followed by RP)-HPLC and SDS-PAGE followed by RP-HPLC. Tandem mass spectrometric analysis was executed using an ESI-linear ion trap after which proteins were identified and functionally classified. Due to the complementary nature of the techniques and the incorporation of several technical replicates the authors were able to identify a large number of CVF proteins. Also, a less stringent identification methodology was used in this study, which may also account for this high number. Additionally, the authors focused on the kallikrein family (i.e. family of 15 secreted serine proteases with tryptic or chymotryptic activity) and confirmed the presence of different kallikreins (kallikrein 6, 7, 10, 11, 12 and 13) in CVF by ELISA [44]. Further research on this topic by the same authors revealed that kallikreins may play a physiologically active role in the desquamation of vaginal epithelial cells and activation of cervicovaginal antimicrobial proteins. The authors also hypothesized that cervicovaginal kallikrein expression may result in pathological conditions (e.g. PPROM) [50,51].

Klein *et al*. [14] performed proteome analyses on CVF samples from pregnant women who had symptoms of pending preterm birth. The authors identified 40, mainly high abundant, proteins using a shotgun proteomics approach using nanoscale RP-HPLC coupled to an ESI-ion trap mass spectrometer and showed that this method yields reproducible and reliable results. Additionally, they analyzed CVF using 2D-PAGE and demonstrated that the 2D-PAGE profiles are very similar for different samples, but that the spot intensities showed a certain degree of intersample variation.

Our group [22] also used proteomics technology to characterize the CVF proteome. During a colposcopy, the cervicovagina is rinsed with a 5% acetic acid solution in order to make HPV infected lesions visible. Normally, this washing fluid is discarded but was used by our group to perform CVF proteomics studies. Using C_4_-RP-HPLC fractionation on the protein level followed by C_18_-RP-HPLC on the peptide level, and MALDI-TOF/TOF mass spectrometry, we were able to identify 339 proteins. During these experiments, we also determined the technical variability of the platform. Three technical replicates were analyzed and 68% of the different proteins identified, were detected in all three replicates. Analysis of the chromatographic separations showed a high degree of reproducibility (coefficient of variation was 0.56%). This led to the conclusion that the largest contribution to the technical variation is made by the mass spectrometric analysis. Also, using an in-house build relational database, we compared the largest proteomics studies on CVF and determined a set of proteins which are identified in the vast majority of the studies, independent of patient physiology or used analytical methods. This overlapping protein set was the first step towards the delineation of the CVF core proteome (see below) [22].

Finally, two studies have been performed on cervical mucus [36,37]. Because cervical mucus is part of the CVF (see above), these studies can be seen as an analysis of a subfraction of the CVF proteome. Andersch-Björkamn *et al*. [36] used 1D-PAGE and SDS-agarose composite gel electrophoresis to separate proteins and subsequently performed RP-nano-HPLC peptide separations. Peptides were identified using ESI-FTICR tandem mass spectrometry. The authors identified 178 proteins from cervical mucus. Furthermore, they analyzed protein and mucin composition and the mucin *O*-glycosylation of the cervical mucus at different time points during the menstrual cycle. The results showed that cervical mucus was similar before and after ovulation, but differed at the time of ovulation. This observation was also seen in alterations of the *O*-glycosylation of the mucus. The physiological significance of these changes is not yet understood [36]. Panicker *et al*. [37] recently performed a study on the human cervical mucus proteome. The authors used 2D-PAGE and 1D-PAGE followed by nanocapillary RP-HPLC. Tandem mass spectrometry was performed using an ESI-Q-TOF to characterize the cervical mucus proteome. Immunodepletion of albumin and immunoglobulins did not result in a significant improvement. Although the authors noticed an overlap between the different experimental setups, each platform yielded a certain amount of unique proteins, indicating the importance of using different techniques in order to maximize proteome coverage. The authors also characterized the phosphorylation and glycosylation of cervical mucus proteins and found 14 proteins which were posttranslationally modified [37].

The studies described above primarily aimed to catalogue the CVF proteome. Therefore, in what follows, some upcoming facts of the CVF proteome will be reviewed in more detail. Beside these studies, four proteomic studies on CVF have been performed which focused on the detection of differences in protein expression profiles due to HIV resistance and pending preterm birth [35,41,52,53]. These quantitative studies are discussed further below.

## The cervicovaginal proteome: numbers and facts

852 different proteins were identified in diverse antibody-independent proteomics studies on human CVF and cervical mucus (listed in additional file 1) [14,22,36-38,40,42-45]. Also, during our latest experiments we were able to identify an additional 256 CVF proteins (unpublished data), making it a total of 1108 different CVF proteins which are identified thus far (listed in additional file 2). 252 and 257 proteins were also found in respectively plasma [54,55] and amniotic fluid [56-58] (shown in additional file 2). 733 proteins from the CVF proteome were not identified in plasma or amniotic fluid.

Additionally, 223 proteins were identified in cervical mucus [36,37]. Of those, 51 proteins were uniquely identified in cervical mucus (additional file 2) while the greater part (172 proteins) was also identified in studies on CVF [14,22,38,40,42-45]. This was to be expected because cervical mucus is a subfraction of CVF. This suggests that analysis of subfractions of the CVF proteome may still yield new identifications [22].

**Figure 1:**
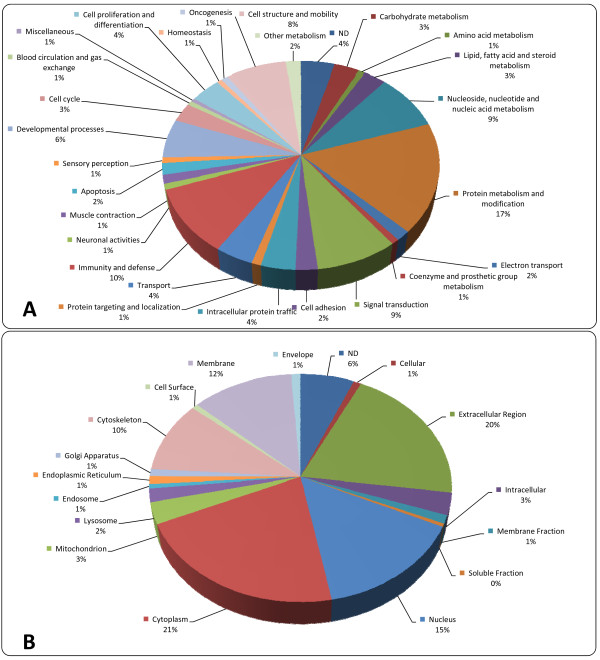
**Functional classification (A) and classification according to cellular localization (B) of all identified CVF proteins**. All proteins identified in the proteomics studies listed in table 1 were classified according to several general functional and cellular localization terms.

Functional classification (Figure 1A) of the CVF proteome shows a great diversity of biological roles of which "protein metabolism and modification" and "immunity and defense" are the largest categories (respectively 17% and 10%). Classification based upon cellular localization (Figure 1B) shows that most proteins are present in the cytoplasm or in the extracellular region (respectively 21% and 20%)

**Figure 2:**
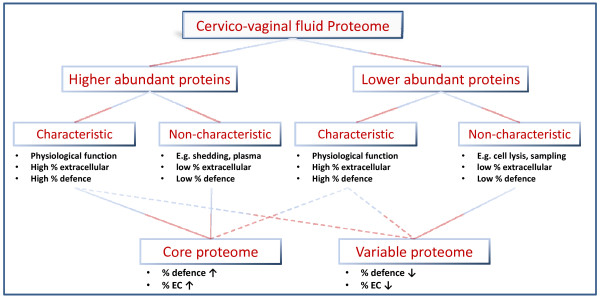
**Schematic overview of the CVF proteome composition**. The CVF proteome can be roughly divided into "higher" and "lower" abundant proteins. The proteins from these categories can be further subdivided based on their function and cellular localization. The term "characteristic" is used here for proteins with a function and cellular localization which is representative for the functions of CVF. "Non-characteristic" is used for proteins which presumably do not have a measurable or known significant biological function in this body fluid. For each category, a general indication of the percentage immunological and extracellular is given. Proteins from the subcategories subsequently construct the "core" and "variable" proteome.

Figure 2 gives a schematic overview of the proteome structure of the CVF. Although exceptions and deviations from this generalized scheme are most likely, it may help in explaining the difficulties encountered during CVF biomarker discovery. Roughly, a proteome consists of a plethora of proteins which are present in either higher concentrations (hence easier to identify) or lower concentrations (usually difficult to identify). The difference between the highest and the lowest protein concentration in a proteome is described as the dynamic range, which is specific for a particular sample and important to take into account when performing comprehensive proteomics studies. Indeed, the higher the dynamic range of the sample, the more difficult it is to describe its proteome as complete as possible. Implementation of different separation dimensions, optimization of the techniques and sample preparation may increase the sensitivity and resolving power of the proteomics platform, thus allowing the detection of more proteins.

Because CVF is an extracellular fluid with an important role in the innate immunity, it can be expected that physiological relevant proteins are extracellular, immunological molecules. We call these "characteristic" proteins since, based on their function and localization, they represent the fundamental characteristics of CVF. Both high and low abundant CVF proteins may fulfill a physiological function in the cervicovagina. For example, we found that the most abundant protein in CVF is S100A9 [22] which forms with S100A8 the calprotectin heterodimer. Calprotectin is a potent cervicovaginal antimicrobial peptide and inhibits bacterial growth by sequestration of zinc. Its importance is also reflected in the numerous physiological and pathological processes (e.g. cervical cancer, pregnancy and labour) for which correlations have been found with differences in calprotectin expression levels [59]. Examples of low abundant physiological relevant proteins are β-defensins or histones. In response to infectious stimuli, histones can create *neutrophil extracellular traps *(NET) (i.e. protrusions made of a DNA backbone in which histones, proteases and antimicrobial peptides/proteins are embedded) which are efficient in trapping and eliminating pathogens [60-62].

However, a substantial fraction of high and low abundant proteins may also originate from aberrant processes. For instance, sampling of CVF may cause disruption of the epithelial cell layer (e.g. when using brushes or tampons) and the continuous shedding of the epithelial cell layer may also bring in dead cells. These processes introduce many intracellular proteins (e.g. metabolic or cytoskeletal proteins) in the CVF which have no significant biological function in this body fluid. We therefore call these proteins "non-characteristic" since their function and localization do not match the CVF related characteristics.

In general, it can be assumed that due to the extracellular nature of the CVF and its importance in the innate immunity, the characteristic proteins will contain larger amounts of extracellular and immunological fractions as compared to the non-characteristic proteins.

Another part of the CVF is made of non-characteristic low abundant proteins of which the concentration shows large intra- and interindividual variations. These concern often intracellular proteins which take part in metabolic processes (e.g. glycolysis).

Although the higher abundant characteristic proteins may contain very interesting biomarkers, potential biomarkers usually reside in the lower abundant part of the proteome. Therefore, one of the main challenges is the isolation of the biomarker from the numerous surrounding background proteins. How can these two be distinguished? Since background proteins are usually sporadically and randomly detected, they show no correlation with a particular physiological or pathological condition. In contrast, there must be a clear and significant association between alterations in the concentration of a biomarker and a specific condition or pathology.

Taking all of the above into account, the CVF proteome can roughly be divided into two subfractions: the core and the variable proteome. The core proteome consists of proteins which are identified in most proteomics studies and therefore mainly represents high abundant (characteristic and non-characteristic) proteins, although characteristic low abundant proteins are also present. Hence, we expect the fraction of characteristic proteins to be high in the core proteome. We recently described a set of 136 proteins which are frequently identified, independent of patient physiology or used analytical methods. This subproteome has an increased extracellular immunological protein fraction [22], indicating that it is more relevant for CVF (as compared to the complete set of overlapping and non-overlapping proteins). It can therefore be considered as a first version of the core proteome. In contrast, the variable proteome contains proteins which are infrequently identified and often represent non-characteristic low abundant proteins. Therefore, the extracellular and immunological fraction is decreased in this part.

This subdivision may explain some dissimilarities seen between the different proteomics studies on CVF. The fractions of immunological and extracellular proteins are lower in the 'larger' studies of Shaw *et al*. [44] and Zegels *et al*. [22] (respectively 11.72% and 12.57%) as compared to the 'smaller' studies from Pereira *et al*. [43] and Dasari *et al*. [42] (respectively 18.95% and 19.61%). The same holds for the extracellular fraction [22]. In a recent experiment, we identified 706 proteins from which 267 proteins were new identifications (unpublished results). Only 5.33% of these proteins fell into the "immunity and defense" functional category, which is much lower than was found in any other study (see above).

**Figure 3:**
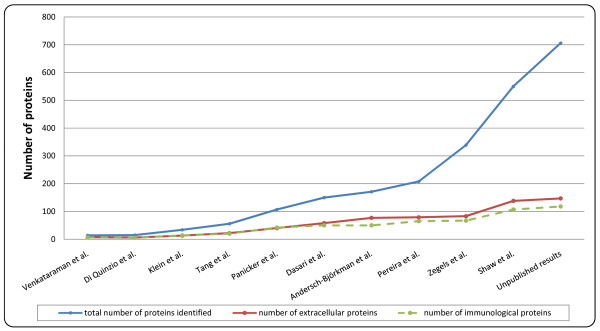
**Comparison of the total number of proteins versus the number of extracellular and immunological proteins**. For each study listed in table 1 the number of extracellular (red line), immunological (green line) proteins and total number of identifications (blue line) were determined. The obtained results were plotted progressively from the study with the lowest number of identifications to the study with highest number of identifications.

A general trend is noticeable in proteomic analysis of CVF towards the identification of increasing quantities of different proteins using comprehensive techniques. This tendency was facilitated by the drastic technological evolution which proteomics has undergone the last 5 to 10 years. The development of sensitive, high resolution mass spectrometers with fast scan rates allows the coverage of dynamic ranges up to six orders of magnitude. The dynamic range of the experimental setup was further enhanced by improving HPLC separation prior to mass spectrometric analysis (e.g. increased sensitivity and reproducibility and use of columns with higher peak capacity). Moreover, progress has been made in multidimensional separations in order to further increase the peak capacity. All together, the technological developments in proteomics resulted in analytical platforms which can now cover larger parts of the proteome [63]. This is also reflected in the studies on the CVF proteome where increasing numbers of proteins were identified with the contemporary techniques (Figure 3).

It is the common opinion that by digging deeper into the CVF proteome, more potential biomarkers could be isolated and multi-protein expression profiles can thus be analyzed. With this reasoning however, one must take into account some inherent disadvantages of large proteomic studies. In Figure 3, the total number of proteins identified and the numbers of immunological or extracellular proteins (which are characteristic for the CVF proteome) in the different studies are plotted. From this, it is clear that while the total number of identified proteins increases exponentially, the immunological and extracellular fraction only increases linearly. This graph indicates therefore that the majority of newly obtained identifications have no known function or their function in the CVF is questionable or not biologically relevant. We therefore hypothesize that although large number of proteins are identified in the recent sensitive large proteomics studies, the vast majority of newly identified proteins are most likely part of the variable proteome which consist mainly of non-characteristic proteins. Thus by digging deeper into the CVF proteome, the challenge of identifying specific biomarkers among this large amount of non-characteristic proteins increases significantly. Optimization of sample collection methods to minimize cell lysis may be a step forward to reduce the amount of non-characteristic proteins. On the other hand, epithelial cell shedding is an essential process in the removal of pathogens and many other factors (e.g. hormonal regulation, non-oral contraceptives) will always cause cell disruption and lysis, prior to sample collection.

## Cervicovaginal fluid biomarkers with potential clinical applications

The lower genital tract is exposed to a large microbial pressure and forms an important entry gate for a wide variety of pathogens such as *Candida *spp., *Trichomonas vaginalis*, *human immunodeficiency virus *(HIV) and *human papillomavirus *(HPV). In contrast, the upper female genital tract is believed to be sterile under normal circumstances. Nevertheless, pathogens such as *Neisseria gonorrhoeae *and *Chlamydia trachomatis *can occasionally ascend from the lower to the higher genital tract and can cause pathogenic conditions such as pelvic inflammatory disease (PID). The clinical impact of these infections may not be underestimated since they can lead to serious conditions such as preterm birth, increased susceptibility to sexually transmitted infections (STI), infertility and cancer [1,2]. Therefore, early diagnosis is essential for the successful treatment of the underlying pathology and to prevent irreversible complications. For this reason, there is a high demand for new diagnostic tools, such as biomarkers, which allow for better and earlier treatment of gynecological pathologies [17].

In the following paragraphs some important infection-correlated pathologies are discussed for which potential biomarkers were discovered using human CVF samples. Table 2 gives an overview of these potential biomarkers.

**Table 2:** Overview of potential CVF protein/peptide biomarkers for different diseases, conditions or statuses of the female genital tract.

*Condition or patient status*	*Potential CVF protein/peptide biomarker*	*Reference*	*Also found in larger proteomic studies*
**HPV infection and cervical cancer**			

	Il-6	[190,191]	
	Il-8	[190,191]	
	IL-12p40n	[65,192]	
	IFN-γ	[65,192]	
	Il-10	[65,192]	
	TGF-β1	[65,192]	
	TNF-α	[65,192]	
	Il-1β	[65,192]	
	Il-5	[193]	
	Anti HPV IgG	[194,195]	
	Anti HPV IgA	[196]	
	antigalactosyl (α1→3) galactose antibodies	[197]	

**HIV resistance**			

	Anti-HIV IgA and IgG antibodies	[103-113]	
	RANTES	[100,114]	

**Bacterial Vaginosis**			

	antimicrobial peptides (e.g. SLPI, defensins, lysozyme and lactoferrin)	[142-147]	[14,22,36-38,40,42-45]
	IL-1β	[198-208]	
	IL-8	[198,201,204,207,209-211]	
	IL-10	[120,128,210]	
	IL-4	[198,210]	

**Preterm Birth**			

	fetal fibronectin	[162-168]	[36,43,44]
	C-reactive protein	[212]	
	interleukin-6	[212-217]	
	interleukin-8	[215,216,218-220]	
	interleukin-1β	[220]	
	granulocytic elastase α1-antiprotease	[221,222]	
	prolactin	[223]	
	sialidase	[224]	
	monocyte chemotactic protein 1	[225]	
	insulin-like growth factor-binding protein-1	[226,227]	[43]
	defensins	[142]	[22,36-38,42-44,147]
	lactoferrin	[228]	[14,22,36-38,42-44]
	matrix metalloproteinases	[212]	[36,42-44]
	β-human chorionic gonadotrophin	[229,230]	

**PPROM**			

	β-human chorionic gonadotropin	[178,183,231-236]	
	insulin-like growth factor binding protein-1	[226,227,237-246]	[43]
	diamine oxidase	[240,247-251]	
	active ceruloplasmin	[252]	[36,37,42-45]
	fetal fibronectin	[250,253]	[36,43,44]
	C-reactive protein	[254]	
	α-fetoprotein	[183,231,250,255-259]	
	prolactin	[231,260-263]	
	placental α-microglobulin-1	[184]	
	interleukin-1 receptor antagonist	[46]	[22,40,42-45]

The table lists CVF protein biomarkers identified in non-proteomic studies using mainly antibody-dependent techniques (e.g. ELISA). Last column shows, when possible, the proteomics studies in which these biomarkers were also detected. Biomarkers identified the proteomics studies are not presented.

### Human papillomavirus infection and cervical cancer

HPV is one of the most common sexually transmitted infections. It is estimated that 70-80% of the sexually active female population will acquire HPV before the age of 50 [64] and 80% of the HPV-infected patients will spontaneously clear the virus within 2 years [65,66]. HPV is associated with high morbidity and mortality worldwide, since it can cause precancerous lesions and cervical cancer. The latter is the second most observed cancer in women and is caused for nearly 100% by HPV-infection [67]. There are more than 150 HPV types and almost 40 of them infect the anogenital region. HPV can be divided in low and high risk HPV. The most common low-risk types are type 6 and 11. The most common high-risk types are 16, 18, 45, 31 and 33). Cervical cancer is mainly caused by an infection with a high risk type [68]. Infection of squamous or glandular epithelial cells with HPV causes morphological alterations which lead to the formation of squamous or glandular intra-epithelial lesions. The severity of this precancerous condition depends on the extent, the site as well as on the depth of the infected epithelial layer [67,69].

Because of the high prevalence, the severe consequences and the lack of a good treatment of advanced cervical carcinoma, the emphasis of managing this condition lies on prevention and diagnosis in a stadium as early as possible. The recently introduced prophylactic HPV vaccination will prevent about 98% of infections with HPV 16 and 18 but will also in part, due to so called cross protection, prevent HPV 31 (89%), HPV 33 (82%), HPV 45 (100%) and any high risk HPV type 16/18/31/33/35/39/45/51/52/56/58/59/66/68 (70%). As such, the HPV vaccination will prevent about 70 - 80% of all cervical cancers [70,71]. Recently, clinical trials started with a nine-valent HPV vaccine which could potentially prevent 90% of all cervical cancers but information on this topic is still scarce. Therefore, it is still needed to screen women between the ages of 25 and 65 on a frequent basis by means of a Pap test (liquid based cytology) preferable with a type specific HPV testing. Screening using the classical Pap test has led to a considerable reduction of the mortality, yet there are still some notable shortcomings. The current screening is (1) labour intensive, (2) difficult to automatize, (3) vulnerable to inter- and intra-observer variability and (4) is not very sensitive [65,72,73]. Moreover research has demonstrated that despite intensive screening campaigns, the coverage in most Western countries stays low (e.g. 59% in Belgium) [74]. Testing for the presence of HPV DNA or RNA will increase the sensitivity, but it cannot predict progression to intra-epithelial lesions and eventually cervical cancer.

Taking all the above into account, this implicates that alternative screening methods (e.g. based upon protein biomarkers present in CVF) with both a higher sensitivity and specificity are needed. The identification of a highly sensitive and specific protein biomarker from CVF would mean a substantial improvement both on medical and social-economical field because an accurate biomarker for the detection of cervical neoplasia will result undoubtedly in a more precise and efficient screening which correlates with a further reduction of precancerous morbidity and cervical cancer morbidity and mortality [75]. Additionally, proteomic analysis of CVF could lead to biomarkers for the evaluation of patients after treatment [76] or to prognostic biomarkers which could predict the evolution from precancerous low-grade squamous intra-epithelial lesions (LSIL) to either high-grade squamous intra-epithelial lesions (HSIL) or clearance of the lesions. These prognostic biomarkers would benefit the screening method as the number of follow-up patients could be reduced significantly (up to 80%). Also, identification of proteins involved in the clearance of the virus could be of major importance for the future development of new antiviral therapies.

However, only few studies are performed on human CVF for the identification of such biomarkers as most large proteomic studies were restricted to cervical cancer tissue [77-82]. Studies which used CVF as samples for their analysis did not use antibody-independent proteomics techniques, but mainly ELISA or Western blotting, and were therefore restricted to the analysis of a small number of proteins taking part in the immune response against HPV. In table 1, several proteins are listed which are associated with HPV infection, cervical cancer and cancer prognosis and progression. Nevertheless, these proteins are primarily correlated with the prognosis of the disease but do not allow for a sensitive and/or specific prediction of healthy women which are at high risk. It is our opinion that such potential biomarkers have the highest chance to be discovered in a proteome wide approach, using proteomics techniques on a large number of samples.

#### HIV-resistance

More than 25 million people have already died because of HIV since the discovery of the virus in 1981, making it one of the most disastrous epidemics in human history. In 2008, 33.4 million people were living with HIV and 2.7 million people got infected by the virus [83,84], mainly due to heterosexual HIV transmission [84-86].

One of the largest problems remains the absence of an effective therapy. Also, despite the enormous efforts made, the development of a prophylactic AIDS-vaccine will likely not be available in the recent years to come [87]. This leads to an increased importance for microbicides (i.e. chemical entities that can prevent or reduce transmission infections) which can be applied locally (e.g. vaginally or rectally) as a potential alternative approach in HIV prevention [9,88,89]. However, the efficacy trials often gave rise to unsatisfactory results like enhanced HIV-transmission, toxicity and immune activation [41,90]. To overcome these problems, new powerful microbicides need to be identified which preferentially occur naturally and act under these *in vivo *circumstances against sexually transmitted HIV infections [41].

Analysis of samples obtained from *exposed seronegative individuals *(ESNs) may yield such new, therapeutically relevant microbicides. ESNs are individuals (<5% of the population) from high-risk cohorts that remain IgG-seronegative despite frequent HIV-exposure [91] and therefore show a certain degree of *in vivo *HIV-resistance. ESNs are found between commercial sex workers, hemophiliacs receiving HIV-contaminated blood, healthcare workers, children from HIV-infected mothers, intravenous drug users or they are the seronegative partner in a discordant couple [92-94].

Some biological factors are already associated with the ESN-status. Those include innate host factors like mutations of chemokine receptors (e.g. CCR5-Δ32), the upregulation of chemokines such as RANTES, MIP-1α and -1β due to genetic polymorphisms, specific human leukocyte antigen haplotypes (e.g. HLA-B*27 and HLA-B*57), natural killer cell activity regulated by killer Ig like receptor/HLA interaction, the presence of autoantibodies and/or alloantibodies and more efficient immunological effects after *toll like receptor *stimulation. Also, particular properties of the acquired immune system of the host show correlation with the ESN-status such as cytotoxic and helper T lymphocyte responses against HIV epitopes, humoral immune responses with the production of neutralizing anti-HIV antibodies and the presence of soluble inhibitory factors [92-99].

Generally, none of these associations alone can be held fully responsible for the observed HIV-resistance, and many unknown factors are yet to be discovered [41]. Current studies speculate that ESNs inhibit HIV-infection in a very early phase of viral transmission [100,101]. HIV-resistance may for example result from mechanisms present at the viral entry gate, which often is the mucosa of the female genital tract. Among these mechanism, proteins or peptides present in CVF of ESNs may play an important role [41].

Several studies on female ESNs have used CVF as choice of sample for analysis. It has been described that cervicovaginal lavages from ESNs sometimes contain gp41 and p24 HIV antigens, suggesting that there was an HIV exposure, but no seroconversion could be detected [102]. Anti-HIV IgA and IgG antibodies were detected in CVF obtained from heterosexual ESN women [103-113]. Additionally, it has been shown that levels of the HIV suppressive β-chemokine RANTES levels were significantly higher in CVF from ESNs [100,114]. Moreover, CVF contains proteins/peptides with intrinsic anti-HIV activity such as defensins, lactoferrin, lysozyme, cathelicidin and SLPI [22,92,115,116]. The proteomic study from Venkataraman *et al*. on CVF showed that the cationic fraction of CVF has natural anti-HIV activity. The authors speculated that this activity is the result from a complex synergism between different proteins in CVF [38]. It is possible that differences in protein expression patterns of those anti-HIV proteins/peptides may result in the observed HIV-resistance of some ESN cohorts. However, conventional techniques such as ELISA or Western blotting are incapable to analyze complex differential patterns from multiple proteins. Therefore, proteomics studies may aid in the elucidation of these factors.

Indeed, recently, Burgener *et al*. [35] studied HIV-resistance in ESN persons and used 2D-DIGE as a quantitative proteomics technique to compare protein expression patterns from ESNs with those from control groups. The authors identified 16 differentially expressed proteins with different biological functionalities such as protease inhibitors (e.g. serpin B3/B4/B13/B1 and cystatin A) and proteins with an immunological role (e.g. protein S100A7 and complement component 3). Iqbal *et al*. [41] used *surface enhanced laser desorption ionization *(SELDI)-TOF mass spectrometry for the analysis of the CVF proteome of a large population of ESNs and control groups. The authors demonstrated that elafin/trappin-2 is significantly upregulated in ESNs [41]. Elafin/trappin-2 is an antiprotease from the whey acidic protein (WAP) family, of which SLPI is also a member, that can be found in CVF (see above). It functions as an anti-inflammatory protein by counteracting proteases secreted by neutrophils. Due to the cationic nature of elafin/trappin-2 it can destabilize bacterial cell walls or viral envelopes, thus exerting a certain degree of antimicrobial activity [117]. At about the same time, elafin/trappin-2 was identified as a new anti-HIV factor which directly interacted with the virus for its inhibition, thus making it an interesting protein for further investigation [118]. Hence, both studies confirm each other, thereby increasing the possibility that elafin/trappin-2 effectively plays a significant role in HIV-resistance. Surprisingly, this protein was not among the differentially expressed proteins in the study of Burgener *et al*. [35] where a different technique was used (2D-PAGE vs. SELDI). These results indicate once again that different proteomics techniques complement each other and are necessary to uncover further other CVF proteins correlating with *in vivo *HIV-resistance. Moreover the use of samples obtained from dissimilar ethnological cohorts may yield different results as it becomes more evident that several different mechanisms may lead to HIV-resistance.

#### Bacterial vaginosis

Bacterial vaginosis (BV) is a frequently observed condition by which the normal vaginal flora is disrupted. The prevalence of this pathology in Europe is 5-30%. Other geographical regions, especially those with a lower socio-economic status like the sub-Saharan part of Africa, show higher prevalences (over 50%) [119-121].

BV is a non-inflammatory condition, although it is often classified as a vaginitis, characterized by the loss of hydrogen peroxide forming *Lactobacillus *spp. (e.g. *L. crispatus, L. acidophilus, L. rhamnosus) *and by an increased growth of anaerobic species (e.g. *Atopobium vaginae, Mobiluncus *spp., *Prevotella *spp.). Also, whereas a healthy vaginal flora contains only few different and predominantly *Lactobacillus *species, the vaginal flora correlated with BV is polymicrobial in nature [119]. The question remains whether the loss of the *Lactobacillus *spp. is due to the introduction of a yet unknown factor which results in the passive growth of the anaerobic species or, vice versa, whether there is a massive influx of anaerobic species which eradicate the lactobacilli [122,123]. Although BV is not classified as a sexually transmitted infection, the condition has several features indicating that it may be caused by an exogenous source [124]. Nevertheless, the etiology of BV is not yet well understood.

BV can lead to severe health problems and early diagnosis can prevent serious complications. A significant correlation exists between the presence of BV and preterm birth and it has been shown that treatment of BV may reduce the risk on preterm birth [119,120,124,125]. BV has also been linked with PID, miscarriage [124] and infertility [126] increased susceptibility for HIV and HPV [124,127-132], herpes simplex virus type 2 [133-135] and other lower genital tract pathogens [120].

The mechanism by which BV leads to those pathological conditions is not well known. It has been suggested that increased susceptibility to HIV and genital tract infections may be attributed to the production of succinate by anaerobes, which can inhibit neutrophilic function [136]. Also, some anaerobes produce sialydases which effect immune cells (e.g. inhibition of phagocytes) [137] and have mucinase activity [138]. Additionally, *Ureaplasma urealyticum *can produce proteases which can cleave IgA [139]. It has also been shown that CVF from some seropositive and seronegative women with BV has the capability to enhance HIV replication *in vitro*. This activity has been attributed to a certain soluble and protease sensitive HIV-inducing factor (HIF) which has not been identified yet. The general opinion hypothesizes that HIF is a mixture of products derived from BV associated bacteria on the one hand and host factors such as certain cytokines (e.g. myeloid-related protein 8) on the other hand [140,141].

No large proteomics studies have been performed on CVF from women with BV, but several ELISA experiments were executed. The results show that BV CVF samples are deficient in antimicrobial activity due to lower expression levels of several antimicrobial peptides such as SLPI, defensins, lysozyme and lactoferrin [142-147]. Furthermore, many studies also detected differences in expression levels of some proinflammatory cytokines correlated with BV (see table 1). These studies suggest that BV leads to the disruption of the normal innate and adaptive immune response and induces an inflammatory environment in the lower genital tract which can lead to increased susceptibility for HIV and other genital tract infections [120,128,148]. However, since only antibody dependent studies were performed, analysis was limited to a few well characterized proteins. Nevertheless, little is known about expression differences of multiple proteins, which may include new or unexpected ones. Differential analysis of the CVF proteome using proteomics approaches, may yield more information about the perturbation of the female genital tract immunity due to BV and further clarify the etiology of the disease.

#### Preterm Birth

As mentioned before, conditions such as bacterial vaginosis and other infections of the female genital tract are the main cause of preterm birth. Other risk factors include multiple pregnancy or uterine contractions of cervical incompetence. Nevertheless, a significant part of the observed preterm births are idiopathic, indicating that the etiology of preterm birth is still not well understood [149-151].

Preterm birth, defined by the WHO as birth occurring before 37 completed weeks of gestation, is a major cause of neonatal morbidity and mortality [152,153]. Recent reports estimate the incidence of preterm birth 9.6% worldwide, with the highest rates in Africa and North America (respectively 11.9% and 10.6%) and the lowest in Europe (6.2%). The impact of preterm birth on the health of the neonate can be very severe on short-term (e.g. respiratory stress syndrome, brain injury) as well as on long-term (e.g. chronic lung disease, sensory deficits, learning disabilities) thus affecting the child's development and the physical and psychological health of the infant's environment [153-155].

Preterm birth is difficult to treat, therapy is often inefficient because the underlying cause is seldom determined and the diagnosis is frequently made too late [154,156-159].

Due to the clinical importance of preterm birth, prevention strategies need more attention in the management of this condition [157,160]. Currently this is achieved by assessing the risk of the occurrence of preterm birth using several parameters or markers. History of preceding preterm birth is the most important risk factor and may relate to cervical anatomical problems. In addition, presence of any of the causal factors mentioned before drastically increases the chance on preterm birth [149,161]. CVF fetal fibronectin (FFN) concentrations are also correlated with the risk for preterm labour/birth, but quantification of CVF FFN alone has not enough discriminatory power [162-168]. Combination of this test with cervical ultrasound measurements however showed better correlations [166,169-172]. Several other markers have been correlated with the risk on preterm birth/labour, all of which are summed in table 1. Nevertheless, low specificity and precision are inherent to these primary predictors.

Therefore, new markers need to be identified which allow for more precise and specific risk assessment [149,157]. Besides, these markers may give additional information about the etiology and/or pathophysiology of this condition which can be used in the development of alternative therapies [43].

As mentioned before, Pereira *et al*. [43] identified potential biomarkers for preterm birth/labour in a proteomics study. These included fibronectin, several S100 proteins (calgranulin A, B and C), acute-phase reaction proteins (annexin A3, α-1-antitrypsin and α-1-acid glycoprotein) and proteins involved in cellular organization and motility (e.g. profilin-1, thymosin β-4, rho GDI 2).

Recently, the same research group looked for biomarkers for intra-amniotic infection, which is an important risk factor for preterm birth and may lead to severe neonatal pathologies. 170 CVF samples were classified in three different groups: intra-amniotic infection and preterm birth; no intra-amniotic but preterm birth; no intra-amniotic infection and preterm labour. The samples underwent 2D-LC (SCX/RP) combined with ESI-QTOF mass spectrometry. Quantification and comparison of protein expression levels between the three groups was achieved by spectral counting. The authors found 26 potential biomarkers for intra-amniotic infection (statistical significant expression differences), which included acute phase reactants (e.g. α1-antitrypsin), immune modulators (e.g. lysozyme and α-defensin), amniotic fluid proteins (e.g. haptoglobin) and extracellular matrix-signaling proteins (e.g fibronectin). 13 of those were confirmed as potential biomarkers for intra-amniotic infection in immunoassays performed on the 170 samples. Using four of these proteins (α1-acid-glycoprotein, insulin-like growth factor binding protein-1, calgranulin C and cystatin A) in a logistic regression model, a fairly accurate differentiation between patients with intra-amniotic infection and those without intra-amniotic infection could be obtained. Although further clinical validation of these potential biomarkers is required, these results may lead to the development of new diagnostic assays for intra-amniotic infection. Also, the authors showed that the etiology of preterm birth caused by intra-amniotic infection is different than preterm birth without infection [52].

Shah *et al*. [53] used the *stable isotope labeling by amino acids in cell culture *(SILAC) technique to label proteins from an endocervical and vaginal cell-line (respectively End1 and Vk2). After the labeling process, a labeled cellular secretome (i.e. a "standard secretome") from vaginal and endocervical cells could be purified. CVF proteins can then be relatively quantified in test samples in a precise and accurate way by means of this "standard secretome" and *multiple reaction monitoring *(MRM). This technique is called *stable isotope labeling proteome *(SILAP). Normally, MRM requires the addition of labeled synthetic proteotypic peptides, which are often very expensive, but the use of SILAP has the advantage that the labeled "standard secretome" can be used instead. Nevertheless, since the concentration of the peptides in the "standard secretome" is not accurately known, it is not possible to absolutely quantify proteins in the test samples. A set of 15 candidate CVF biomarkers were thus relatively quantified and expression profiles were compared between healthy controls and preterm birth cases. This resulted in three proteins which were significantly upregulated (desmoplakin isoform 1, stratifin and thrombospondin), but the possible role of these proteins in the pathogenesis of preterm birth is not yet known [53].

#### Preterm premature rupture of membranes

Many studies have been performed on preterm premature rupture of membranes (PPROM) which is an important risk factor of preterm birth (see above). PPROM is defined as "rupture of the fetal membranes prior to onset of labour in a patient who has a gestational age of less than 37 weeks" and has an incidence of 3-5% of all pregnancies [173-178]. A number of CVF proteins and peptides are associated with this condition and are listed in table 1. Also other non-protein markers are associated with PPROM such as lactate [173,179,180], urea [181] and creatinine [181-183]. However, no differential proteomics studies have been performed focusing on this condition. Nevertheless, it can be expected that, as is the case with the preterm birth in general, the use of proteomic technology may result in new insights in the etiology of PPROM and the isolation of clinical applicable biomarkers.

Amnisure^® ^International LLC (Cambridge, MA) has developed the FDA approved AmniSure^® ^ROM test for the diagnosis of PPROM. This simple immunoassay identifies placental α-microglobulin-1 which is abundant in amniotic fluid (2000-25000 ng/ml), but almost undetectable in cervicovaginal secretions (5-25 ng/ml). Only in the case of PPROM, higher concentrations are detectable in CVF. Analysis of clinical application possibilities of the test has shown a sensitivity of 99% and a specificity of 88-100% [184,185]. This test is a good example of the potential of CVF samples as biomarker sources and their applicability in clinical diagnosis. Although the test is still under investigation, the clinical use of such non-invasive test may eventually replace traditional diagnostic methods [177].

## Classical approaches versus new techniques

The vast majority of potential CVF biomarkers listed in table 1 was identified and validated using antibody-dependent classical approaches such as Western blotting or ELISA. Only the last few years, the use of proteomics as a new alternative technique for biomarker discovery has gained more interest due to the evolution of techniques such as chromatography and mass spectrometry from low reproducible and highly specialized methods to more user friendly, robust systems. The use of comprehensive proteome characterization and analysis has the great advantage that a huge amount of proteins (at least those which fall in the dynamic range of the applied techniques) are under consideration as potential biomarkers. Other techniques such as ELISA require the use of antibodies, which may restrict investigation to those biomarkers for which antibodies are available and purchased. Additionally, antibody-based techniques are sometimes incapable of discriminating between different subtle protein forms, whereas mass spectrometry can solve this problem. For example, it has been published that two α-defensins (HNP 1 and 2) are very sensitive and specific biomarkers for intra-amniotic infection. However, using ELISA, it is not possible to discriminate between HNP1 and 2 due to high sequence similarity. In contrast, due to the mass difference between HNP1 and 2, mass spectrometry can differentiate between both forms [186,187].

Nevertheless, this does not imply that proteomics is superior to antibody dependent methods in biomarker discovery experiments and makes them completely obsolete. Indeed, antibody-based validation is often required to confirm proteomics results. Also, as can be seen in table 1, a large part of the biomarkers identified in non-proteomics studies, are not identified in any of the CVF proteome characterization studies mentioned above. Therefore, techniques such as Western blotting or ELISA are rather complementary then inferior to proteomics.

## Conclusions

CVF has great potential as a source for biomarkers for pathologies of the female genital tract. Many details about the complex physiology of the female genital tract, even under normal circumstances, are yet unknown and only few proteomics studies have been executed on this topic [39,46,47]. Nevertheless, CVF may yield a wealth of information about the "normal" functioning of the female genital tract and proteomic analysis may improve our understanding of micro-environmental changes due to the influence of multifaceted conditions such as menstrual cycle, age or pregnancy.

Despite the progress made in the discovery phase (table 1), not many of the potential biomarkers find their way towards a clinical application. Only the AmniSure^® ^ROM test for the diagnosis of PPROM, based upon the detection of placental α-microglobulin-1, has been further validated in clinical trials and is FDA approved. The problem in the validation of biomarkers and the subsequent development of biomarker based diagnostic tests, often lies in the transition from discovery to validation phase. Frequently, too little effort has been made in setting up statistical relevant experiments and important aspects such as sample size, inter- and intraindividual and technical variabilities are often underestimated or not well defined. This inevitably leads to results which lack statistical power and to potential biomarkers which generally do not survive further validation [153,178,188,189]. In order to obtain statistically significant and practically useable highly specific and sensitive CVF biomarkers, several steps must be undertaken. 1) It is essential to analyze and characterize the CVF proteome precisely to be able to define the physiological environment in which experiments are conducted [18]. 2) Also, the inter- and intraindividual variability must be determined, which is critical in order to determine the number of samples needed to obtain statistically relevant results and to distinguish real biomarkers from natural occurring biophysiological variations. So far, although quantitative and comparative experiments were performed on CVF, not much attention has been given to the contribution of biological variation [16,24]. A good understanding of the effect of factors affecting the proteome composition (e.g. age, use of contraceptives, menstrual cycle, etc.) is therefore essential and should be taken into account during sample collection. Additionally, the choice of control groups in the experimental setup can have a drastic effect on the eventual results. For example, some markers (e.g. fibronectin, C-reactive protein, prolactin) for preterm birth are often found in preterm premature rupture of membranes (table 1). Nevertheless, markers for preterm birth which were found to be highly specific, may be in fact low specific if PPROM patients were included in the control group.

Up to now, most studies using CVF as clinical samples, used antibody dependent techniques such as ELISA and Western blotting, although these are only capable to provide very limited amounts of information so that important data are inevitably missed. Nevertheless, many of these studies improved our understanding of different pathologies and conditions. However, the use of proteomic technology has the advantage to analyze many different proteins at the same time and can yield large amounts of information. We strongly believe that quantitative and comparative proteomic analysis of the CVF proteome will yield new complementary insights and information about the biophysiological nature of the female genital tract which may lead to further progression in our understanding of gynecological pathologies. All together, this may result in a better management of these diseases and thus, hopefully, will reduce associated morbidity and mortality for women, mothers and children.

## Abbreviations

2D-DIGE: 2D differential gel electrophoresis; BV: bacterial vaginosis; CVF: cervicovaginal fluid; ESI: electrospray ionization; ESN: exposed seronegative individuals; FFN: fetal fibronectin; FTICR: fourier transform ion cyclotron resonance; HIF: HIV-inducing factor; HIV: human immunodeficiency virus; HLA: human leukocyte antigen; HNP: human neutrophil peptide; HPV: human papillomavirus; HSIL: high-grade squamous intra-epithelial lesions; IEF: isoelectric focusing; IL-1RA: interleukin-1 receptor antagonist; LC: liquid chromatography; LSIL: low-grade squamous intra-epithelial lesions; MALDI: matrix assisted laser desorption ionization; MIP: macrophage inhibitory protein; MRM: multiple reaction monitoring; MS: mass spectrometry; ND: not determined; NET: neutrophil extracellular traps; PAGE: polyacrylamide gel electrophoresis; PID: pelvic inflammatory disease; PPROM: preterm premature rupture of membranes; Q: quadrupole; RANTES: regulated on activation, normal T-cell expressed and secreted; RP: reversed phase; SCX: strong cation exchange; SELDI: surface enhanced laser desorption ionization; SILAC: stable isotope labeling by amino acids in cell culture; SILAP: stable isotope labeling proteome; SLPI: secretory leukocyte protease inhibitor; STI: sexually transmitted infection; TOF: time-of-flight; WAP: whey acidic protein

## Competing interests

The authors declare that they have no competing interests.

## Authors' contributions

GZ was responsible for the research and the writing of the manuscript. GAAVR and WAAT wrote a significant part about proteomics and HPV infection, provided advice and reviewed the manuscript. XWMVO was responsible for the supervision and thorough review of the manuscript. All authors read and approved the final manuscript.

## Supplementary Material

Additional file 1 **Overview of all identifications obtained in different proteomic studies on human CVF**. Click here for file

Additional file 2 **List of proteins identified from CVF and cervical mucus**. Swissprot accession number, the protein name, cellular localization and the different biological functions of the protein are given. Yellow highlighted proteins were also identified in human amniotic fluid, green highlighted proteins were identified in human serum and orange highlighted proteins were identified in serum as well as in amniotic fluid. Proteins marked with * are uniquely identified in cervical mucus and not in any other study on human CVF. Click here for file
