# Supplementary material for: Use of cervicovaginal fluid for the identification of biomarkers for pathologies of the female genital tract
Source: Proteome Sci. 2010 Dec 8;8:63. doi: 10.1186/1477-5956-8-63 (PMC3016264; doi:10.1186/1477-5956-8-63)
Supplement: Additional file 1 — Overview of all identifications obtained in different proteomic studies on human CVF. [file 1477-5956-8-63-S1.PDF]

Additional file 1 – Overview of all identifications obtained in different proteomic studies on human CVF

Accession No	Protein Name	Venkataraman et al, 2005[1]	Di Quinzio et al, 2007[2]	Dasari et al, 2007[3]	Tang et al, 2007[4]	Shaw et al, 2007[5]	Pereira et al, 2007[6]	Andersch-Björkman et al, 2007[7]	Klein et al, 2008[8]	Zegels et al, 2009[9]	Panicker et al, 2010[10]
681073A	Haptoglobin alpha1S				√						
A5YKK6	CCR4-NOT transcription complex, subunit 1 isoform a					√					
A6NHG4	D-dopachrome decarboxylase-like protein					√					
A6NL28	Putative tropomyosin alpha-3 chain-like protein									√	
A8K2U0	Alpha-2-macroglobulin-like protein 1 precursor					√				√	
A8K5I0	Heat shock 70 kDa protein 1B							√			
A8MQ03	UPF0574 protein C9orf169									√	
A8MQC9	Uncharacterized protein CLCA4					√					
A9Z1Y9	Thymosin beta-4-like protein 6									√	
BAC01816	Immunoglobulin λ light chain VLJ				√						
O00151	PDZ and LIM domain protein 1					√					
O00204	Sulfotransferase family cytosolic 2B member 1					√					
O00299	Chloride intracellular channel protein 1				√			√			
O00391	Sulfhydryl oxidase 1 precursor					√		√			
O00468	Agrin precursor							√			
O00555	Voltage-dependent P/Q-type calcium channel subunit alpha-1A									√	
O00584	Ribonuclease T2 precursor					√					
O00592	Podocalyxin-like precursor isoform 1							√			
O00754	mannosidase, alpha, class 2B, member 1 precursor					√					
O14745	Ezrin-radixin-moesin-binding phosphoprotein 50					√		√			
O15031	plexin-B2 precursor					√					
O15144	Actin-related protein 2/3 complex subunit 2									√	
O15145	Actin-related protein 2/3 complex subunit 3					√					
O15231	Zinc finger protein 185					√				√	
O15260	Surfeit locus protein 4					√					
O15263	Beta-defensin 2 precursor									√	
O15393	Transmembrane protease, serine 2 precursor					√					
O15400	syntaxin 7					√					
O15457	MutS protein homolog 4					√					
O43175	D-3-phosphoglycerate dehydrogenase					√				√	
O43240	Kallikrein-10 precursor					√				√	
O43278	Kunitz-type protease inhibitor 1 precursor					√					
O43451	Maltase-glucoamylase, intestinal					√					
O43490	Prominin 1							√			
O43497	Voltage-dependent T-type calcium channel subunit alpha-1G					√					
O43707	Actinin, alpha 4			√		√	√	√		√	
O43781	Dual-specificity tyrosine-phosphorylation regulated kinase 3					√					
O43852	Calumenin precursor					√					
O43866	CD5 antigen-like precursor					√					
O60218	Aldo-keto reductase family 1, member B10 (aldose reductase)					√	√				
O60235	Transmembrane protease, serine 11D precursor			√		√	√			√	
O60259	Kallikrein-8 precursor					√					
O60346	PH domain leucine-rich repeat										√
O60391	Glutamate [NMDA] receptor subunit 3B precursor					√					
O60437	Periplakin			√		√	√			√	
O60504	Vinexin			√							
O60664	Mannose-6-phosphate receptor binding protein 1						√			√	
O60814	Histone H2B type 1-K									√	
O75015	Fc-gamma receptor IIIb					√					
O75083	WD repeat protein 1					√					
O75131	Copine-3						√				
O75223	Protein C7orf24			√		√	√				
O75339	Cartilage intermediate layer protein							√			

Accession No	Protein Name	Venkataraman et al, 2005[1]	Di Quinzio et al, 2007[2]	Dasari et al, 2007[3]	Tang et al, 2007[4]	Shaw et al, 2007[5]	Pereira et al, 2007[6]	Andersch-Björkman et al, 2007[7]	Klein et al, 2008[8]	Zegels et al, 2009[9]	Panicker et al, 2010[10]
O75351	Vacuolar protein sorting-associating protein 4B					√					
O75367	Core histone macro-H2A.1					√					
O75369	Filamin-B					√				√	
O75448	Thyroid hormone receptor-associated protein complex 100 kDa component					√					
O75556	Secretoglobin family 2A, member 1							√			
O75594	Peptidoglycan recognition protein precursor						√				
O75629	Protein CREG1 precursor					√					
O75882	Attractin precursor					√		√			
O94823	Probable phospholipid-transporting ATPase VB									√	
O94919	Endonuclease domain-containing 1 protein precursor					√					
O95171	Sciellin			√		√	√			√	
O95274	Ly6/PLAUR domain-containing protein 3 precursor					√				√	
O95361	Tripartite motif-containing protein 16					√					
O95831	Programmed cell death protein 8, mitochondrial precursor					√					
O95994	Anterior gradient protein 2 homolog precursor										√
P00338	L-lactate dehydrogenase A chain					√	√	√		√	
P00390	Glutathione reductase, mitochondrial precursor					√	√				
P00441	Superoxide dismutase [Cu-Zn]		√	√		√	√			√	
P00450	Ceruloplasmin precursor			√	√	√	√	√			√
P00491	Purine nucleoside phosphorylase				√	√	√				
P00492	Hypoxanthine-guanine phosphoribosyltransferase						√				
P00558	Phosphoglycerate kinase 1			√		√	√	√		√	√
P00738	Haptoglobin			√	√	√	√	√		√	√
P00746	Complement factor D precursor							√			
P00747	Plasminogen precursor			√		√					
P00748	Coagulation factor XII precursor					√					
P00751	Complement factor B precursor					√	√	√			√
P00797	Renin							√			
P00915	Carbonic anhydrase 1							√		√	√
P00918	Carbonic anhydrase 2							√		√	
P01008	Antithrombin-III precursor			√	√	√		√			
P01009	Alpha-1-antitrypsin precursor		√	√		√	√	√	√	√	√
P01011	Alpha-1-antichymotrypsin precursor			√		√		√	√		√
P01019	Angiotensinogen precursor [Contains: Angiotensin I						√	√			
P01023	Alpha-2-macroglobulin precursor					√	√	√			
P01024	Complement component 3			√	√	√	√	√		√	√
P01028	Complement C4 precursor			√			√				
P01033	Metalloproteinase inhibitor 1 precursor									√	
P01034	Cystatin-C							√		√	
P01040	Cystatin A (stefin A)	√	√	√	√	√	√	√		√	√
P01042	Kininogen			√			√	√			
P01591	Immunoglobulin J chain			√		√	√	√		√	
P01593	Ig kappa chain V-I					√					
P01597	Ig kappa chain V-I region DEE			√							
P01605	Ig kappa chain V-I region Lay									√	√
P01606	Ig kappa chain V-I region OU										√
P01616	Ig kappa chain V-II region MIL					√					
P01617	Ig kappa chain V-II region TEW			√			√				√
P01619	Ig kappa chain V-III region B6										√
P01620	Ig kappa chain V-III region SIE			√							√
P01625	Ig kappa chain V-IV region Len			√		√	√				
P01700	Ig lambda chain V-I region HA										√
P01701	Ig lambda chain V-I region NEW						√				
P01703	Ig lambda chain V-I region NEWM			√							
P01708	Ig lambda chain V-II region BUR					√					
P01714	Ig lambda chain V-III region SH					√					

Accession No	Protein Name	Venkataraman et al, 2005[1]	Di Quinzio et al, 2007[2]	Dasari et al, 2007[3]	Tang et al, 2007[4]	Shaw et al, 2007[5]	Pereira et al, 2007[6]	Andersch-Björkman et al, 2007[7]	Klein et al, 2008[8]	Zegels et al, 2009[9]	Panicker et al, 2010[10]
P01743	Ig heavy chain V-I region HG3 precursor					√					
P01766	Ig heavy chain V-III region BRO					√	√				√
P01768	Ig heavy chain V-III region CAM					√					
P01771	Ig heavy chain V-III region HIL					√					
P01772	Ig heavy chain V-III region KOL						√				
P01833	Polymeric immunoglobulin receptor			√		√	√	√	√	√	√
P01834	Ig kappa chain C region			√		√	√	√	√	√	√
P01842	Ig lambda chain C regions			√		√	√		√	√	√
P01857	Ig gamma-1 chain C region			√	√	√	√	√	√	√	√
P01859	Ig gamma-2 chain C region			√		√	√			√	√
P01860	Ig gamma-3 chain C region						√	√		√	
P01861	Ig gamma-4 chain C region			√		√	√			√	
P01871	Ig mu chain C region			√		√	√	√			
P01876	Ig alpha-1 chain C region			√		√	√	√		√	√
P01877	Ig alpha-2 chain C region			√		√				√	
P02042	Hemoglobin subunit delta									√	√
P02100	Hemoglobin subunit epsilon									√	
P02511	Alpha-crystallin B chain					√				√	
P02545	Lamin-A/C					√	√			√	
P02647	Apolipoprotein A1			√	√	√	√	√		√	√
P02652	Apolipoprotein A-II precursor									√	
P02671	Fibrinogen alpha chain precursor			√		√	√	√		√	
P02675	Fibrinogen beta chain precursor			√	√	√	√	√		√	
P02679	Fibrinogen gamma chain			√		√	√	√			
P02730	Solute carrier family 4,anion exchanger,member 15							√			
P02749	Apolipoprotein H (beta-2-glycoprotein I)			√		√	√			√	
P02750	Leucine-rich alpha-2-glycoprotein precursor						√				
P02751	Fibronectin precursor					√	√	√			
P02760	AMBP protein precursor [Contains: Alpha-1-microglobulin						√				
P02763	Alpha-1-acid glycoprotein 1			√	√	√	√	√		√	√
P02765	Alpha-2-HS-glycoprotein			√			√	√		√	
P02766	Transthyretin		√		√	√	√	√		√	√
P02768	Serum albumin precursor	√	√	√	√	√	√	√	√	√	√
P02774	Vitamin D-binding protein precursor			√	√	√	√	√		√	
P02787	Serotransferrin			√	√	√	√	√	√	√	√
P02788	Lactotransferrin			√	√	√	√	√	√	√	√
P02790	Hemopexin			√		√	√	√		√	√
P02792	Ferritin light chain					√		√			
P03973	Antileukoproteinase 1 precursor			√		√	√	√	√	√	√
P04003	C4b-binding protein alpha chain precursor					√					
P04004	Vitronectin			√			√	√			√
P04040	Catalase			√	√	√	√				√
P04075	Fructose-bisphosphate aldolase A			√		√	√			√	√
P04080	Cystatin B	√		√	√	√	√	√		√	√
P04083	Annexin A1			√	√	√	√	√	√	√	√
P04114	Apolipoprotein B-100 precursor									√	
P04179	Superoxide dismutase [Mn], mitochondrial										√
P04196	Histidine-rich glycoprotein precursor					√		√			
P04207	Ig kappa chain V-III region CLL [Precursor]						√				
P04208	Ig lambda chain V-I region WAH					√					
P04217	Alpha-1-B glycoprotein				√		√	√			
P04279	Semenogelin-1			√						√	
P04406	Glyceraldehyde-3-phosphate dehydrogenase, liver			√	√	√	√	√	√	√	√
P04433	Ig kappa chain V-III region VG precursor									√	√
P04745	Alpha-amylase							√			
P04792	Heat-shock protein beta-1			√		√	√	√	√	√	√

Accession No	Protein Name	Venkataraman et al, 2005[1]	Di Quinzio et al, 2007[2]	Dasari et al, 2007[3]	Tang et al, 2007[4]	Shaw et al, 2007[5]	Pereira et al, 2007[6]	Andersch-Björkman et al, 2007[7]	Klein et al, 2008[8]	Zegels et al, 2009[9]	Panicker et al, 2010[10]
P05089	Arginase-1					√					√
P05090	Apolipoprotein D precursor					√					
P05107	Integrin beta-2 precursor					√					
P05109	Calgranulin A (S100A8)	√		√	√	√	√	√	√	√	√
P05154	Plasma serine protease inhibitor precursor					√		√		√	
P05155	Plasma protease C1 inhibitor precursor					√		√			
P05156	Complement factor I precursor (C3B/C4B inactivator)							√			
P05164	Myeloperoxidase precursor			√		√	√	√	√	√	
P05204	Non-histone chromosomal protein HMG-17			√						√	
P05386	60S acidic ribosomal protein P1			√							
P05387	60S acidic ribosomal protein P2			√				√		√	
P05388	60S acidic ribosomal protein P10			√							
P06312	Ig kappa chain V-IV region precursor						√				√
P06314	Ig kappa light chain VLJ region							√			
P06396	Gelsolin					√	√	√			
P06576	ATP synthase subunit beta, mitochondrial [Precursor]										√
P06702	Calgranulin B (S100A9)	√		√	√	√	√	√	√	√	√
P06703	Protein S100-A6										√
P06727	Apolipoprotein A-IV							√			
P06731	Carcinoembryonic antigen-related cell adhesion molecule 5 precursor			√		√		√		√	
P06733	Alpha-enolase			√	√		√	√		√	√
P06744	Glucose-6-phosphate isomerase							√		√	
P06753	Tropomyosin 3					√	√			√	
P07108	Acyl-CoA binding protein		√	√		√	√			√	
P07237	Protein disulfide-isomerase precursor			√		√	√				√
P07305	Histone H1.0									√	
P07339	Cathepsin D precursor					√	√	√			
P07355	Annexin A2			√	√	√	√	√	√	√	√
P07384	Calpain-1 catalytic subunit					√	√				
P07437	Tubulin, beta polypeptide							√			
P07476	Involucrin			√		√	√		√	√	
P07686	Beta-hexosaminidase beta chain precursor					√					
P07737	Profilin 1			√	√	√	√	√		√	√
P07858	Cathepsin B			√		√	√	√			
P07900	Heat shock protein HSP 90-alpha 2			√		√	√				√
P07911	Uromodulin precursor					√					
P07919	Ubiquinol-cytochrome c reductase complex 11 kDa protein					√					
P07948	Tyrosine-protein kinase Lyn					√					
P07951	Tropomyosin beta chain									√	√
P07998	Ribonuclease pancreatic precursor					√					
P08107	Heat shock 70 kDa protein 1			√		√	√			√	√
P08123	Collagen alpha 2 T					√	√	√		√	
P08174	Complement decay-accelerating factor precursor					√					
P08236	Beta-glucuronidase precursor					√					
P08238	Heat shock protein HSP 90-beta					√	√			√	√
P08246	Leukocyte elastase precursor					√	√	√	√	√	
P08311	Cathepsin G	√		√		√	√	√	√	√	
P08603	Complement factor H			√		√	√	√		√	
P08670	Vimentin			√		√	√	√		√	
P08697	Alpha-2-antiplasmin precursor					√					
P08708	40S ribosomal protein S17									√	
P08758	Annexin A5; Calphobindin I				√		√	√			√
P08833	Insulin-like growth factor binding protein 1						√				
P09211	Glutathione S-transferase P		√	√	√	√	√	√		√	√
P09429	High mobility group protein B1					√					
P09466	Glycodelin precursor									√	

Accession No	Protein Name	Venkataraman et al, 2005[1]	Di Quinzio et al, 2007[2]	Dasari et al, 2007[3]	Tang et al, 2007[4]	Shaw et al, 2007[5]	Pereira et al, 2007[6]	Andersch-Björkman et al, 2007[7]	Klein et al, 2008[8]	Zegels et al, 2009[9]	Panicker et al, 2010[10]
P09493	Tropomyosin alpha-1 chain					√					√
P09497	Clathrin light chain B									√	
P09525	annexin IV					√		√			
P09651	Heterogeneous nuclear ribonucleoprotein A1					√				√	
P09668	Cathepsin H precursor					√					
P09758	Tumor-associated calcium signal transducer 2 precursor					√					
P09960	Leukotriene A-4 hydrolase					√					
P09972	Fructose-bisphosphate aldolase C					√					
P0AA25	IgE Fv SPE7 chain B complexed with a recomb.thioredoxin							√			
P0C0L4	Complement C4-A										√
P0C0L5	Complement C4-B precursor					√					
P0C0S5	Histone H2A.Z (H2A/z).					√					
P0C0S8	Histone H2A type 1									√	
P0C869	Cytosolic phospholipase A2 beta									√	
P10153	Nonsecretory ribonuclease precursor					√	√				
P10155	60 kDa SS-A/Ro ribonucleoprotein					√					
P10253	Alpha-glucosidase							√			
P10412	Histone H1.4									√	
P10599	Thioredoxin		√	√	√	√	√			√	√
P10606	Cytochrome c oxidase subunit 5B, mitochondrial precursor									√	
P10619	Lysosomal protective protein precursor					√					
P10909	Clusterin precursor					√		√		√	
P11021	78 kDa glucose-regulated protein			√		√	√	√			√
P11142	Heat shock 70kDa protein 8			√		√	√			√	√
P11216	Glycogen phosphorylase, brain form					√					
P11413	glucose-6-phosphate dehydrogenase isoform a				√	√					
P11499	Heat shock protein HSP 90-beta							√			
P12036	Neurofilament heavy polypeptide									√	
P12273	Prolactin-inducible protein precursor					√				√	
P12429	Annexin A3		√	√	√	√	√	√		√	√
P12724	Eosinophil cationic protein precursor			√		√	√			√	
P12814	Alpha-actinin-1					√				√	
P12821	Angiotensin I converting enzyme isoform 1							√			
P12830	E-cadherin					√					
P13611	Versican core protein precursor					√					
P13639	Elongation factor 2			√		√	√				
P13667	Protein disulfide-isomerase A4 precursor					√					
P13671	Complement component C6 precursor					√		√			
P13796	Lymphocyte cytosolic protein 1 (L-plastin)			√	√	√	√		√	√	√
P13797	Plastin-3					√				√	
P13929	Beta-enolase					√					
P13987	CD59 glycoprotein precursor			√		√	√			√	
P14136	Glial fibrillary acidic protein									√	
P14174	Macrophage migration inhibitory factor									√	
P14314	Glucosidase 2 subunit beta precursor					√					
P14317	Hematopoietic lineage cell-specific protein					√					
P14384	Carboxypeptidase M							√		√	
P14550	Alcohol dehydrogenase [NADP+]										√
P14618	Pyruvate kinase isozymes M1/M2			√		√	√	√		√	√
P14625	Endoplasmin precursor					√					√
P14780	Matrix metalloproteinase-9 precursor			√		√	√	√			
P14854	Cytochrome c oxidase subunit VIb isoform 1					√					
P14923	Desmoplakin-3			√		√	√				
P15056	B-Raf proto-oncogene serine/threonine-protein kinase									√	
P15104	Glutamine synthetase									√	√
P15144	Membrane alanine aminopeptidase precursor							√			

Accession No	Protein Name	Venkataraman et al, 2005[1]	Di Quinzio et al, 2007[2]	Dasari et al, 2007[3]	Tang et al, 2007[4]	Shaw et al, 2007[5]	Pereira et al, 2007[6]	Andersch-Björkman et al, 2007[7]	Klein et al, 2008[8]	Zegels et al, 2009[9]	Panicker et al, 2010[10]
P15153	Ras-related C3 botulinum toxin substrate 2 precursor					√		√			
P15259	Phosphoglycerate mutase 2					√				√	
P15289	Arylsulfatase A precursor					√					
P15309	Prostatic acid phosphatase precursor					√					
P15311	Ezrin					√				√	
P15538	cytochrome P450, family 11, subfamily B, polypeptide 1 isoform 2 precursor					√					
P15924	Desmoplakin			√		√	√			√	
P16035	Metalloproteinase inhibitor 2									√	
P16401	Histone H1.5 (Histone H1a)			√		√	√			√	
P16402	Histone H1.3			√						√	
P16403	Histone H1.2						√			√	
P16870	Carboxipeptidase E				√						
P17066	Heat shock 70 kDa protein 6										√
P17174	Aspartate aminotransferase, cytoplasmic							√			
P17213	bactericidal/permeability-increasing protein precursor					√					
P17858	liver phosphofructokinase isoform b					√					
P17900	Ganglioside GM2 activator precursor					√				√	
P17931	Galectin-3					√				√	
P18054	Arachidonate 12-lipoxygenase, 12S-type					√				√	
P18136	Ig kappa chain V-III region HIC [Precursor]					√					
P18206	Vinculin			√		√	√			√	
P18510	Interleukin 1 receptor antagonist protein		√	√	√	√	√			√	
P18621	60S ribosomal protein L17 (L23) isoform 5					√				√	
P18669	Phosphoglycerate mutase 1			√		√	√	√		√	
P18858	DNA ligase 1					√					
P18859	ATP synthase, H+ transportine, mitochondrial FO complex,					√					
P19021	Peptidylglycine alpha-amidating monooxygenase							√			
P19105	Myosin regulatory light chain 2, nonsarcomeric					√					
P19120	Heat shock cognate 71 kDa protein							√			
P19440	Gamma-glutamyl transpeptidase							√			
P19447	TFIIH basal transcription factor complex helicase XPB subunit									√	
P19652	Alpha-1-acid glycoprotein 2 precursor					√	√	√			
P19827	Inter-alpha (globulin) inhibitor H1							√			
P19957	Elafin precursor					√				√	
P19961	Alpha-amylase 2B precursor					√					
P20020	Plasma membrane calcium-transporting ATPase1					√					
P20061	Transcobalamin-1 precursor					√					
P20160	Azurocidin 1 (cationic antimicrobial protein 37)					√	√	√		√	
P20670	Histone H2A.o	√		√							
P20700	Lamin-B1					√					
P20810	Calpastatin (Calpain inhibitor) (Sperm BS-17 component)			√		√	√			√	
P20908	Collagen alpha-1(V) chain precursor					√					
P20930	Filaggrin					√				√	
P21128	Placental protein 11 precursor					√					
P21333	Filamin-A					√	√				
P21589	5' nucleotidase,ecto							√			
P21817	Ryanodine receptor 1					√					
P22090	40S ribosomal protein S4, Y isoform 1					√					
P22314	Ubiquitin-activating enzyme E1					√					
P22352	Plasma glutathione peroxidase							√			
P22528	Cornifin B			√		√	√			√	
P22531	Small proline-rich protein 2E									√	
P22532	Small proline-rich protein 2D			√			√			√	
P22626	Heterogeneous nuclear ribonucleoprotein A2/B1					√					
P22735	Protein-glutamine gamma-glutamyltransferase K			√		√				√	
P22894	Neutrophil collagenase precursor					√	√				

Accession No	Protein Name	Venkataraman et al, 2005[1]	Di Quinzio et al, 2007[2]	Dasari et al, 2007[3]	Tang et al, 2007[4]	Shaw et al, 2007[5]	Pereira et al, 2007[6]	Andersch-Björkman et al, 2007[7]	Klein et al, 2008[8]	Zegels et al, 2009[9]	Panicker et al, 2010[10]
P23083	Ig heavy chain V-I region V35 precursor						√				
P23142	Fibulin-1 precursor					√		√		√	
P23246	Splicing factor, proline-and gluatmine-rich					√					
P23284	peptidylprolyl isomerase B precursor					√		√			
P23396	40S ribosomal protein S3					√					
P23526	Adenosylhomocysteinase					√		√			
P23528	cofilin-1			√		√		√		√	
P23786	Carnitine O-palmitoyltransferase 2, mitochondrial									√	
P24158	Myeloblastin precursor			√		√	√			√	√
P24821	Tenascin C							√			
P25311	Alpha-2-glycoprotein 1, zinc				√	√		√			
P25685	DnaJ homolog subfamily B member 1									√	
P25774	Cathepsin S precursor					√					
P25789	Proteasome subunit alpha type 4					√					
P25815	S100 calcium binding protein P					√	√				
P26038	Moesin			√		√	√				
P26373	60S ribosomal protein L13									√	
P26447	S100 calcium binding protein A4						√				
P26641	Elongation factor 1-gamma					√					
P27105	Erythrocyte band 7 integral membrane protein							√			
P27169	Serum paroxonase 1							√			
P27482	Calmodulin-like protein 3			√		√	√			√	
P27487	Dipeptidyl peptidase 4					√		√			
P27797	Calreticulin precursor					√					√
P27816	Microtubule-associated protein 4			√							
P27824	Calnexin precursor					√					
P27918	Properdin precursor					√					
P28001	Histone H2A.a						√				
P28066	Proteasome subunit alpha type 5										√
P28799	Granulins precursor			√		√	√				
P29034	Protein S100-A2						√				
P29218	Inositol monophosphatase					√					
P29373	Cellular retinoic acid-binding protein 2			√		√	√			√	
P29401	Transketolase				√	√	√				
P29508	Squamous cell carcinoma antigen 1 (SCCA-1); Serpin B3		√	√	√	√	√	√	√	√	√
P29590	Probable transcription factor PML					√					
P30041	Peroxiredoxin-6									√	
P30043	Flavin reductase					√				√	√
P30044	Peroxiredoxin-5, mitochondrial precursor							√			√
P30046	D-dopachrome tautomerase						√				
P30050	60S ribosomal protein L12					√					
P30086	Phosphatidylethanolamine-binding protein; Prostatic binding protein			√	√	√	√	√		√	
P30101	Glucose regulated protein, 58kDa; protein disulfide-isomerase A3			√							
P30153	Serine/threonine-protein phosphatase 2A 65 kDa regulatory subunit A					√					
P30456	HLA class I histocompatibility antigen, A-43 alpha chain precursor					√					
P30475	HLA class I histocompatibility antigen, B-39 alpha chain precursor					√					
P30740	Monocyte/neutrophil elastase inhibitor		√	√		√	√	√	√	√	√
P31146	Coronin, actin binding protein, 1A						√				
P31151	S100 calcium-binding protein A7 (psoriasin)			√		√	√			√	√
P31689	DnaJ homolog subfamily A member 1					√					
P31930	Ubiquinol-cytochrome-c reductase complex core protein I					√					
P31942	Heterogeneous nuclear ribonucleoprotein H3					√					
P31944	Caspase-14 precursor					√					
P31946	14-3-3 protein beta/alpha									√	
P31947	Stratifin; 14-3-3 protein sigma			√		√	√			√	√
P31948	Stress-induced-phosphoprotein 1					√					

Accession No	Protein Name	Venkataraman et al, 2005[1]	Di Quinzio et al, 2007[2]	Dasari et al, 2007[3]	Tang et al, 2007[4]	Shaw et al, 2007[5]	Pereira et al, 2007[6]	Andersch-Björkman et al, 2007[7]	Klein et al, 2008[8]	Zegels et al, 2009[9]	Panicker et al, 2010[10]
P31949	S100 calcium-binding protein A11 (calgizzarine)					√	√	√		√	√
P31997	Carcinoembryonic antigen-related cell adhesion molecule 8 precursor									√	
P32119	Peroxiredoxin-2		√			√		√		√	
P32243	Homeobox protein OTX2					√					
P32320	Cytidine deaminase				√	√	√			√	
P32926	Desmoglein-3			√		√	√			√	
P33241	Lymphocyte-specific protein 1					√					
P33778	Histone H2B.f						√				
P34059	N-acetylgalactosamine-6-sulfatase precursor					√					
P34931	Heat shock 70 kDa protein 1L										√
P34932	Heat shock 70 kDa protein 4					√					
P35221	Catenin-alpha-1					√					
P35237	Serpin B6 (Placental thrombin inhibitor)			√		√					
P35268	60S ribosomal protein L22									√	
P35321	Small-proline rich protein 1A; Cornifin A			√		√	√		√	√	
P35325	Small proline-rich protein 2B			√						√	
P35326	Small proline-rich protein 2A			√		√	√		√	√	
P35555	Fibrillin-1 precursor					√					
P35579	Myosin-9					√	√	√		√	
P35658	Nuclear pore complex protein Nup214					√					
P35754	Glutaredoxin-1					√	√				
P35789	Zinc finger protein 93					√					
P36222	Chitinase 3-like 1							√			
P36952	Maspin precursor; Serpin B5 precursor						√				
P36957	Dihydrolipoyllysine-residue succinyltransferase component					√					
P37802	Transgelin-2					√	√				
P37837	Transaldolase			√		√	√				√
P38159	RNA binding motif protein, X-linked-like 1					√					
P38646	Heat shock 70 kDa protein 9					√					
P39019	40S ribosomal protein S19									√	
P39023	60S ribosomal protein L3									√	
P39060	Type XVIII collagen long variant					√					
P40121	Macrophage-capping protein									√	
P40926	Malate dehydrogenase, mitochondrial precursor					√					
P41439	folate receptor 3 precursor					√					
P42677	40S ribosomal protein S27					√					
P42766	60S ribosomal protein L35									√	
P43490	Nicotinamide phosphoribosyltransferase					√					
P43652	Afamin precursor					√		√			
P46108	Proto-oncogene c-crk					√					
P46776	60S ribosomal protein L27a									√	
P46778	60S ribosomal protein L21									√	
P46821	Microtubule-associated protein 1B					√					
P46940	Ras GTPase-activating-like protein IQGAP1					√					
P46976	Glycogenin 1					√					
P47710	Casein alphaS1							√			
P47756	F-actin cappling protein subunit beta					√	√				
P47914	60S ribosomal protein L29									√	
P47929	Lectin, galactoside-binding, soluble, 7 (galectin 7)	√		√		√	√	√		√	
P48594	Squamous cell carcinoma antigen 2; Serpin B4			√	√	√	√			√	
P49061	Metalloproteinase inhibitor 1 precursor							√			
P49189	aldehyde dehydrogenase 9A1					√					
P49327	Fatty acid synthase					√					
P49411	Tu translation elongation factor, mitochondrial					√					
P49773	Histidine triad nucleotide-binding protein 1									√	
P49862	Kallikrein-7 precursor					√					

Accession No	Protein Name	Venkataraman et al, 2005[1]	Di Quinzio et al, 2007[2]	Dasari et al, 2007[3]	Tang et al, 2007[4]	Shaw et al, 2007[5]	Pereira et al, 2007[6]	Andersch-Björkman et al, 2007[7]	Klein et al, 2008[8]	Zegels et al, 2009[9]	Panicker et al, 2010[10]
P49913	Cathelicidin antimicrobial peptide precursor									√	√
P50213	Isocitrate dehydrogenase [NAD] subunit alpha, mitochondrial										√
P50453	Serpin B9					√					
P50750	Cell division protein kinase 9					√					
P50914	60S ribosomal protein L14									√	
P50995	Annexin A11				√		√				
P51149	Ras-related protein Rab-7						√				
P51589	Cytochrome P450 2J2					√					
P51659	Peroxisomal multifunctional enzyme type 2					√					
P51805	Plexin-A3 precursor					√					
P51884	Lumican precursor						√				
P51991	Heterogeneous nuclear ribonucleoprotein A3					√					
P52209	6-phosphogluconate dehydrogenase, decarboxylating						√	√		√	√
P52565	Rho GDP dissociation inhibitor (GDI) alpha						√				
P52566	Rho GDP dissociation inhibitor (GDI) beta				√		√				
P52823	Stanniocalcin 1 precursor							√			
P52907	F-actin capping protein alpha-1 subunit				√	√	√				√
P54108	Cysteine-rich secretory protein 3			√		√	√	√		√	
P54253	Ataxin-1									√	
P54652	Heat shock-related 70 kDa protein 2									√	
P55000	Secreted Ly-6/uPAR-related protein 1 precursor					√					
P55072	Transitional endoplasmic reticulum ATPase					√		√			
P55145	ARMET protein precursor					√				√	
P55196	Afadin (Protein AF-6)					√					
P55786	Puromycin-sensitive aminopeptidase					√	√				
P56537	Eukaryotic translation initiation factor 6					√					
P58062	Serine protease inhibitor Kazal-type 7 precursor					√					
P58546	Myotrophin					√	√				
P59665	Neutrophil defensin 1 precursor	√		√		√	√	√		√	√
P59666	Neutrophil defensin 3 precursor									√	
P60174	Triosephosphate isomerase			√		√	√	√		√	√
P60510	Serine/threonine-protein phosphatase 4 catalytic subunit					√					
P60660	Myosin light polypeptide 6							√			
P60709	Actin, cytoplasmic 1		√	√	√	√	√	√		√	√
P60866	40S ribosomal protein S20									√	
P60900	Proteasome subunit alpha type 6						√				
P60903	S100 calcium binding protein A10; Calpactin I light chain					√	√			√	
P60953	Cell division control protein 42 homolog precursor					√					
P60985	Keratinocyte differentiation-associated protein									√	
P61160	Actin-like protein 2					√	√				
P61254	60S ribosomal protein L26									√	
P61457	Pterin-4-alpha-carbinolamine dehydratase					√					
P61586	Transforming protein RhoA						√				
P61626	Lysozym C	√		√	√	√	√	√	√	√	√
P61769	Beta-2-microglobulin									√	
P61916	Epididymal secretory protein E1						√				
P61978	Heterogeneous nuclear ribonucleoprotein K					√					
P62081	40S ribosomal protein S7									√	
P62158	Calmodulin					√				√	
P62195	Protease regulatory subunit 8, 26S				√						
P62244	40S ribosomal protein S15a					√					
P62249	40S ribosomal protein S16									√	
P62258	14-3-3 protein epsilon										√
P62263	40S ribosomal protein S14									√	
P62266	40S ribosomal protein S23									√	
P62269	40S ribosomal protein S18									√	

Accession No	Protein Name	Venkataraman et al, 2005[1]	Di Quinzio et al, 2007[2]	Dasari et al, 2007[3]	Tang et al, 2007[4]	Shaw et al, 2007[5]	Pereira et al, 2007[6]	Andersch-Björkman et al, 2007[7]	Klein et al, 2008[8]	Zegels et al, 2009[9]	Panicker et al, 2010[10]
P62280	40S ribosomal protein S11									√	
P62318	Small nuclear ribonucleoprotein Sm D3					√					
P62328	Thymosin beta-4			√			√				
P62330	ADP-ribosylation factor 6					√					
P62424	60S ribosomal protein L7a									√	
P62736	Actin, aortic smooth muscle									√	
P62753	40S ribosomal protein S6					√				√	
P62805	Histone H4	√		√		√	√		√	√	√
P62807	Histone H2B.a/g/h/k/l						√				
P62829	60S ribosomal protein L23									√	
P62847	40S ribosomal protein S24									√	
P62851	40S ribosomal protein S25									√	
P62854	40S ribosomal protein S26									√	
P62861	40S ribosomal protein S30									√	
P62888	60S ribosomal protein L30					√					
P62899	60S ribosomal protein L31									√	
P62906	60S ribosomal protein L10a									√	
P62910	60S ribosomal protein L32									√	
P62917	60S ribosomal protein L8									√	
P62937	Peptidyl-prolyl cis-trans isomerase A (Cyclophilin A)			√	√		√	√		√	√
P62988	Ubiquitin			√		√	√			√	√
P63102	14-3-3 zeta isoform							√			
P63104	14-3-3 protein zeta/delta			√			√			√	√
P63167	Dynein light chain 1, cytoplasmic					√					
P63173	60S ribosomal protein L38									√	
P63220	40S ribosomal protein S21									√	
P63241	Eukaryotic initiation factor 5A isoform I variant A					√					
P63313	Thymosin beta-10						√			√	
P67775	Serine/threonine-protein phosphatase 2A catalytic subunit alpha isoform					√					
P67936	Tropomyosin alpha 4 chain			√			√			√	
P68032	Actin, alpha cardiac muscle 1									√	
P68036	Ubiquitin-conjugating enzyme E2 L3						√				
P68104	eukaryotic translation elongation factor 1 alpha 1			√		√		√		√	√
P68366	Tubulin alpha-1 chain					√				√	
P68431	Histone H3.1						√				
P68871	Hemoglobin beta chain					√	√		√	√	√
P69891	Hemoglobin subunit gamma-1									√	
P69905	Hemoglobin alpha subunit					√	√	√	√	√	√
P78417	Glutathione transferase omega 1						√				
P80188	Neutrophil gelatinase-associated lipocalin	√		√	√	√	√	√	√	√	√
P80511	Protein S100-A12				√	√	√			√	√
P80723	Brain abundant, membrane attached signal protein 1			√			√				
P81605	Dermcidin precursor					√				√	
P83731	60S ribosomal protein L24									√	
P84103	Splicing factor, arginine/serine-rich 3									√	
P98088	Mucin-5AC precursor					√	√				
P98187	Cytochrome P450 4F8									√	
P99999	Cytochrome c					√				√	
Q00610	clathrin heavy chain 1					√					
Q00688	FK506-binding protein 3					√					
Q00796	Sorbitol dehydrogenase							√		√	
Q00839	heterogeneous nuclear ribonucleoprotein U isoform a					√					
Q01082	Spectrin beta chain, brain 1					√					
Q01105	Protein SET					√					
Q01459	Di-N-acetylchitobiase precursor					√					
Q01469	Fatty acid-binding protein, epidermal	√	√	√	√	√	√	√	√	√	√

Accession No	Protein Name	Venkataraman et al, 2005[1]	Di Quinzio et al, 2007[2]	Dasari et al, 2007[3]	Tang et al, 2007[4]	Shaw et al, 2007[5]	Pereira et al, 2007[6]	Andersch-Björkman et al, 2007[7]	Klein et al, 2008[8]	Zegels et al, 2009[9]	Panicker et al, 2010[10]
Q01518	CAP, adenylate cyclase-associated protein 1 (yeast)						√			√	
Q01954	Zinc finger protein basonuclin-1									√	
Q02383	Semenogelin-2			√						√	
Q02413	Desmoglein-1 precursor					√	√				
Q02487	Desmocollin-2 precursor			√		√	√			√	
Q02818	Nucleobindin-1 precursor					√					
Q02878	60S ribosomal protein L6									√	
Q03013	glutathione S-transferase M4 isoform 1					√					
Q03252	Lamin B2					√					
Q05524	Alpha-enolase, lung specific			√							
Q05639	Elongation factor 1-alpha 2									√	
Q05682	Caldesmon					√					
Q06323	Proteasome activator complex subunit 1									√	
Q06830	Peroxiredoxin 1			√		√	√	√		√	√
Q07065	Cytoskeleton associated protein 4					√				√	
Q07157	Tight-junction protein ZO-1					√					
Q07654	trefoil factor 3 precursor					√		√		√	
Q08188	Protein-glutamine gamma-glutamyltransferase E precursor					√				√	
Q08380	Galectin-3-binding protein precursor					√		√		√	
Q08EQ4	Thymosin beta-4-like protein 1									√	
Q09666	Neuroblast differentiation-associated protein AHNAK			√		√	√		√	√	
Q0VD83	apolipoprotein B48 receptor					√					
Q10588	ADP-ribosyl cyclase 2 precursor					√	√				
Q12802	A-kinase anchor protein 13 (AKAP 13)					√					
Q12841	Follistatin-related protein 1 precursor					√					
Q12888	Tumor suppressor p53-binding protein 1									√	
Q12889	Oviductal glycoprotein							√			
Q12906	Interleukin enhancer-binding factor 3					√					
Q13011	Delta(3,5)-Delta(2,4)-dienoyl-CoA isomerase, mitochondrial precursor					√					
Q13162	Peroxiredoxin-4					√					
Q13231	Chitotriosidase-1 precursor					√					
Q13283	Ras-GTPase-activating protein-binding protein 1					√					
Q13421	Mesothelin precursor							√			
Q13445	Transmembrane emp24 domain-containing protein 1 precursor					√					
Q13557	calcium/calmodulin-dependent protein kinase II delta isoform 1					√					
Q13614	Myotubularin-related protein 2					√					
Q13765	Nascent polypeptide-associated complex alpha subunit					√					
Q13813	Spectrin alpha chain, brain					√					
Q13835	Plakophilin 1			√		√	√			√	
Q13867	Bleomycin hydrolase					√					
Q13938	Calcyphosine isoform a							√			
Q14055	Collagen alpha-2(IX) chain precursor					√					
Q14116	Interleukin-18 precursor									√	
Q14134	Tripartite motif-containing protein 29					√				√	
Q14166	Tubulin--tyrosine ligase-like protein 12					√					
Q14210	Lymphocyte antigen 6D precursor									√	
Q14508	WAP four-disulfide core domain protein 2 precursor					√		√		√	
Q14515	SPARC-like protein 1 precursor					√		√			
Q14624	Inter-alpha-trypsin inhibitor heavy chain H4 precursor					√	√	√			√
Q14651	Plastin-1										√
Q14677	Clathrin interactor-1					√					
Q14764	Major vault protein					√					
Q14839	Chromodomain helicase-DNA-binding protein 4					√					
Q15056	Eukaryotic translation initiation factor 4H									√	
Q15075	Early endosome antigen 1					√					
Q15084	Protein disulfide-isomerase A6 precursor					√		√			

Accession No	Protein Name	Venkataraman et al, 2005[1]	Di Quinzio et al, 2007[2]	Dasari et al, 2007[3]	Tang et al, 2007[4]	Shaw et al, 2007[5]	Pereira et al, 2007[6]	Andersch-Björkman et al, 2007[7]	Klein et al, 2008[8]	Zegels et al, 2009[9]	Panicker et al, 2010[10]
Q15149	Plectin-1					√				√	
Q15185	Prostaglandin E synthase 3 (Cytosolic prostaglandin E2 synthase)					√					
Q15365	Poly(rC)-binding protein 1					√					
Q15424	Scaffold attachment factor B					√					
Q15459	Splicing factor 3 subunit 1					√					
Q15468	SCL-interrupting locus protein					√					
Q15555	Microtubule-associated protein RB/EB family member 2					√					
Q15642	Cdc-42 interacting protein 4					√					
Q15643	Thyroid receptor-interacting protein 11					√					
Q15651	High mobility group nucleosome-binding domain-containing protein 3									√	
Q15768	Ephrin B3							√			
Q15843	NEDD8									√	
Q15847	Adipose most abundant gene transcript 2 protein									√	
Q16270	Insulin-like growth factor-binding protein 7 precursor					√					
Q16363	Laminin-alpha-4 chain precursor					√					
Q16537	serine/threonine protein phosphatase 2A						√				
Q16610	Extracellular matrix protein 1 precursor			√		√	√			√	
Q16629	Splicing factor, arginine/serine-rich 7									√	
Q16651	Prostasin preproprotein							√			
Q16695	Histone H3.1t									√	
Q16825	Tyrosine-protein phosphatase non-receptor type 21									√	
Q1U7T2	Oligopeptide/dipeptide ABC transporter, ATP-binding protein-like				√						
Q32MZ4	Leucine-rich repeat flightless-interacting protein 1					√					
Q3KNS1	Patched domain-containing protein 3					√					
Q3KQU3	MAP7 domain-containing protein 1									√	
Q3MII2	Serine proteinase inhibitor				√						
Q495M9	Usher syndrome type-1G protein					√					
Q4L180	GPBP-interacting protein 130d					√					
Q4VJB6	14-3-3 protein epsilon isoform transcript variant 1							√			
Q53FA7	Putative quinone oxidoreductase					√					
Q53G64	Anterior gradient 2 homolog							√			
Q53GZ6	Heat shock 70kDa protein 8 isoform 1				√						
Q53RT3	Retroviral-like aspartic protease 1 precursor					√					
Q5CZC0	Fibrous sheath-interacting protein 2									√	
Q5D862	Filaggrin-2 (FLG-2)					√					
Q5H9J7	Protein BEX5					√					
Q5JNX2	Complement component 4a							√			
Q5QNY2	Heat shock 70kDa protein				√						
Q5T0N1	Tetratricopeptide repeat-containing protein					√					
Q5T0Z8	Uncharacterized protein C6orf132									√	
Q5T3I0	G patch domain containing 4 protein isoform 1					√					
Q5TZ20	Olfactory receptor 2G6									√	
Q5TZA2	Rootletin									√	
Q5VTE0	Putative elongation factor 1-alpha-like 3									√	
Q5VTM1	Protein FAM25									√	
Q68CK4	Leucine-rich alpha-2-glycoprotein 1							√			
Q6E0U4	Dermokine precursor					√				√	
Q6IBS0	Twinfilin-2					√					
Q6N089	Ig gamma-1 chain C region					√					
Q6NUR7	Villin 23							√			
Q6P2D8	X-ray radiation resistance-associated protein 1					√					
Q6P3W6	Neuroblastoma breakpoint family member 10									√	
Q6P4A8	Putative phospholipase B-like 1 precursor					√					
Q6PJF2	IgM, Fab fragment of cold agglutinin chain C							√			
Q6UWN5	Ly6/PLAUR domain-containing protein 5 precursor					√					
Q6UWP8	Suprabasin precursor					√				√	

Accession No	Protein Name	Venkataraman et al, 2005[1]	Di Quinzio et al, 2007[2]	Dasari et al, 2007[3]	Tang et al, 2007[4]	Shaw et al, 2007[5]	Pereira et al, 2007[6]	Andersch-Björkman et al, 2007[7]	Klein et al, 2008[8]	Zegels et al, 2009[9]	Panicker et al, 2010[10]
Q6WCQ1	Myosin phosphatase-Rho interacting protein					√					
Q6XPR3	Repetin									√	
Q6ZMR5	Transmembrane protease, serine 11A					√					
Q6ZN66	Guanylate-binding protein 6					√					
Q6ZVX7	Putative uncharacterized protein LOC342897					√				√	
Q71DI3	Histone H3.2									√	
Q71UI9	Histone H2AV					√					
Q71UM5	40S ribosomal protein S27-like protein									√	
Q7L7L0	Histone H2A type 3									√	
Q7Z3Z4	Piwi-like protein 4					√					
Q7Z406	myosin, heavy chain 14 isoform 1					√				√	
Q86SG5	Protein S100-A7-like 1					√				√	
Q86T26	Transmembrane protease, serine 11B					√					
Q86UP2	Kinectin					√					
Q86VD1	MORC family CW-type zinc finger 1					√					
Q86WI1	Fibrocystin L							√			
Q86XP0	Cytosolic phospholipase A2 delta					√					
Q86YZ3	Hornerin					√					
Q8IUE6	Histone H2A type 2-B									√	
Q8IUS5	Abhydrolase domain-containing protein 7									√	
Q8IVV2	Lipoxygenase homology domain-containing protein 1									√	
Q8IY18	SMC5 structural maintenance of chromosomes 5-like 1					√					
Q8IY33	MICAL-like protein 2					√					
Q8IZQ1	WD repeat and FYVE domain-containing protein 3									√	
Q8N0V4	Leucine-rich repeat LGI family member 2 precursor					√					
Q8N1A0	Keratin-like protein KRT222									√	
Q8N257	Histone H2B type 3-B									√	
Q8N2Z9	Centromere protein S					√					
Q8N355	IGLC1 protein					√					
Q8N568	Serine/threonine-protein kinase DCAMKL2					√					
Q8N6Q3	CD177 antigen precursor					√					
Q8N7U6	EF-hand domain-containing family member B					√					
Q8NA31	Coiled-coil domain-containing protein 79									√	
Q8NAC3	Interleukin-17 receptor C precursor									√	
Q8NBI6	Protein C3orf21					√					
Q8NC51	Plasminogen activator inhibitor 1 RNA-binding protein					√					
Q8NCB2	CaM kinase-like vesicle-associated protein					√					
Q8NCR0	UDP-GalNAc:beta-1,3-N-acetylgalactosaminyltransferase 2									√	
Q8NFC6	Protein FAM44A					√					
Q8NGC9	Olfactory receptor 11H4									√	
Q8NHM4	Putative trypsin-6									√	
Q8NHS3	Major facilitator superfamily domain-containing protein 8									√	
Q8TC20	Cancer-associated gene 1 protein									√	
Q8TD31	Coiled-coil alpha-helical rod protein 1									√	
Q8TDC3	BR serine/threonine-protein kinase 1					√					
Q8TDL5	Long palate, lung and nasal epithelium carcinoma associated protein 1			√			√	√	√		
Q8TE68	Epidermal growth factor receptor kinase substrate 8-like protein 1					√					
Q8TER0	Sushi, nidogen and EGF-like domain-containing protein 1									√	
Q8TER5	Protein SOLO									√	
Q8WV44	Tripartite motif protein 41					√					
Q8WVV4	Premature ovarian failure, 1B					√					
Q8WW22	DnaJ homolog subfamily A member 4					√					
Q8WWI1	LIM domain only protein 7					√				√	
Q8WWY7	WAP four-disulfide core domain protein 12 precursor					√					
Q8WXH0	Nesprin-2									√	
Q8WXI7	Mucin-16					√					

Accession No	Protein Name	Venkataraman et al, 2005[1]	Di Quinzio et al, 2007[2]	Dasari et al, 2007[3]	Tang et al, 2007[4]	Shaw et al, 2007[5]	Pereira et al, 2007[6]	Andersch-Björkman et al, 2007[7]	Klein et al, 2008[8]	Zegels et al, 2009[9]	Panicker et al, 2010[10]
Q8WXX0	Ciliary dynein heavy chain 7					√					
Q8WYL5	Protein phosphatase Slingshot homolog 1					√					
Q8WYP5	transcription factor ELYS					√					
Q8WZ42	Titin					√					
Q92520	Predicted osteoblast protein							√			
Q92597	Protein NDRG1					√				√	
Q92614	Myosin-XVIIIa (Myosin containing a PDZ domain)					√					
Q92736	Ryanodine recpetor 2					√					
Q92743	HtrA serine peptidase 1							√			
Q92765	Secreted frizzled-related protein 3 precursor									√	
Q92817	Envoplakin			√		√				√	
Q92820	Gamma-glutamyl hydrolase precursor					√					
Q92876	Kallikrein-6 precursor					√				√	
Q93077	Histone H2A type 1-C									√	
Q93100	Phosphorylase b kinase regulatory subunit beta									√	
Q96BT7	Alkylated DNA repair protein alkB homolog 8					√					
Q96C19	EF-hand domain-containing protein 2					√					
Q96C86	Scavenger mRNA decapping enzyme DcpS					√					
Q96CS3	UBX domain-containing protein 8					√					
Q96F07	cytoplasmic FMR1 interacting protein 2					√					
Q96FF9	Sororin									√	
Q96FQ6	Protein S100-A16									√	
Q96FX8	p53 apoptosis effector related to PMP-22					√					
Q96HC4	PDZ and LIM domain protein 5									√	
Q96HE7	ERO1-like protein alpha precursor					√				√	
Q96JD0	Amyloid lambda 6 light chain variable region SAR					√					
Q96JY6	PDZ and LIM domain protein 2					√					
Q96KC8	DnaJ homolog subfamily C member 1						√				
Q96P63	Serpin B12					√				√	
Q96PQ0	VPS10 domain-containing receptor SorCS2 precursor					√					
Q96QH2	PML-RARA-regulated adapter molecule 1 (PRAM-1) (PRAM)					√					
Q96RY5	Protein cramped-like					√					
Q96S94	Cyclin-L2					√					
Q96TA1	Niban-like protein					√					
Q99102	mucin 4 isoform a					√					
Q99497	DJ-1 protein						√				
Q99523	Sortilin precursor					√					
Q99536	Vesicle amine transport protein 1							√			
Q99538	Legumain precursor					√					
Q99835	Smoothened homolog precursor									√	
Q99877	Histone H2B type 1-N									√	
Q99880	Histone H2B.c	√		√						√	
Q9BPY8	Homeodomain-only protein					√					
Q9BQE3	Tubulin alpha-1C chain									√	
Q9BQR3	Serine protease 27 precursor					√					
Q9BRA2	Thioredoxin-like protein 5					√					
Q9BS26	Thioredoxin domain-containing protein 4 precursor					√					
Q9BW04	specifically androgen-regulated protein					√				√	
Q9BXL7	Caspase recruitment domain-containing protein 11					√					
Q9BYB0	proline-rich synapse-associated protein 2 isoform 1					√					
Q9BYE4	Small proline-rich protein 2G									√	
Q9BYT8	Neurolysin, mitochondrial									√	
Q9BZA7	Protocadherin-11 X-linked precursor					√					
Q9C0A6	SET domain-containing protein 5									√	
Q9C0I9	Leucine-rich repeat-containing protein 27					√					
Q9GZP4	UPF0424 protein C1orf128					√					

Accession No	Protein Name	Venkataraman et al, 2005[1]	Di Quinzio et al, 2007[2]	Dasari et al, 2007[3]	Tang et al, 2007[4]	Shaw et al, 2007[5]	Pereira et al, 2007[6]	Andersch-Björkman et al, 2007[7]	Klein et al, 2008[8]	Zegels et al, 2009[9]	Panicker et al, 2010[10]
Q9GZV4	Eukaryotic translation initiation factor 5A-2									√	
Q9H008	Phospholysine phosphohistidine inorganic pyrophosphate phosphatase					√					
Q9H0W9	Ester hydrolase C11orf54					√					
Q9H1E1	Ribonuclease 7 precursor					√				√	
Q9H201	Epsin-3					√					
Q9H251	Cadherin related 23					√					
Q9H361	Polyadenylate-binding protein 3					√					
Q9H3S7	Tyrosine-protein phosphatase non-receptor type 23					√					
Q9H4M9	EH-domain-containing protein 1					√					
Q9H5V8	CUB domain-containing protein 1					√					
Q9H6S3	Epidermal growth factor receptor kinase substrate 8-like protein 2					√					
Q9H7D7	WD repeat-containing protein 26									√	
Q9HAY6	Beta,beta-carotene 15,15'-monooxygenase									√	
Q9HC84	Mucin-5B precursor			√		√	√			√	
Q9HCE3	Zinc finger protein 532					√					
Q9HD89	Resistin precursor					√					
Q9NQ38	Serine protease inhibitor Kazal-type 5			√		√	√			√	
Q9NR45	Sialic acid synthase					√					
Q9NRL2	Bromodomain adjacent to zinc finger domain protein 1A					√					
Q9NS15	latent transforming growth factor beta binding protein 3					√					
Q9NSY1	BMP-2-inducible protein kinase					√					
Q9NU02	Ankyrin repeat domain-containing protein 5					√					
Q9NX62	Inositol monophosphatase 3					√					
Q9NYK1	Toll-like receptor 7 precursor									√	
Q9NYQ8	Protocadherin Fat 2 precursor					√					
Q9NZH8	Interleukin-1 family member 9					√					
Q9NZQ8	Transient receptor potential cation channel, subfamily M, member 5					√					
Q9NZT1	Calmodulin-like protein 5					√				√	
Q9P0G3	Kallikrein-14									√	
Q9P1Z0	Zinc finger and BTB domain-containing protein 4					√					
Q9P202	Whirlin					√					
Q9P258	Protein RCC2					√					
Q9UBC9	Small proline-rich protein 3			√		√	√		√	√	√
Q9UBD6	Rhesus-associated C glycoprotein					√					
Q9UBG3	Cornulin					√				√	
Q9UBR2	Cathepsin Z precursor					√					
Q9UBX7	Kallikrein 11 precursor			√		√	√			√	
Q9UFN0	Protein NipSnap3A					√					
Q9UGL9	NICE-1 protein			√			√				
Q9UGM3	deleted in malignant brain tumors 1 isoform c precursor					√		√			
Q9UGV6	High mobility group protein 1-like 10						√				
Q9UH77	Kelch-like protein 3					√					
Q9UHG3	Prenylcysteine oxidase precursor					√					
Q9UHL4	Dipeptidyl-peptidase 2 precursor					√					
Q9UI42	Carboxypeptidase A4 precursor					√					
Q9UIV8	Serpin B13					√				√	√
Q9UJY1	Heat shock protein beta-8					√				√	
Q9UKR0	Kallikrein-12 precursor					√					
Q9UKR3	Kallikrein 13 precursor			√		√	√			√	
Q9UL16	Coiled-coil domain-containing protein 19					√					
Q9UL52	Transmembrane protease, serine 11E			√		√				√	
Q9ULH0	Ankyrin repeat-rich membrane spanning protein					√					
Q9ULH4	Leucine-rich repeat and fibronectin type-III domain-containing protein 2					√					
Q9ULV0	Myosin-5B					√				√	
Q9UM54	Myosin-6					√					
Q9UNZ2	NSFL1 cofactor p47					√					

Accession No	Protein Name	Venkataraman et al, 2005[1]	Di Quinzio et al, 2007[2]	Dasari et al, 2007[3]	Tang et al, 2007[4]	Shaw et al, 2007[5]	Pereira et al, 2007[6]	Andersch-Björkman et al, 2007[7]	Klein et al, 2008[8]	Zegels et al, 2009[9]	Panicker et al, 2010[10]
Q9UPP5	KIAA1107 protein					√					
Q9UPQ7	PDZ domain-containing RING finger protein 3									√	
Q9UPT5	Exocyst complex component 7					√					
Q9UPY3	dicer1					√					
Q9Y285	Phenylalanyl-tRNA synthetase alpha chain					√					
Q9Y2B0	MIR-interacting saposin-like protein precursor					√					
Q9Y2V2	Calcium-regulated heat stable protein 1									√	
Q9Y3T6	R3H and coiled-coil domain-containing protein 1									√	
Q9Y446	Plakophilin-3									√	
Q9Y490	Talin-1					√	√				
Q9Y4K1	Absent in melanoma 1 protein					√				√	
Q9Y5Y6	Suppressor of tumorigenicity protein 14					√					
Q9Y6R7	Fc fragment of IgG binding protein					√		√			√
Q9Y6V0	Protein piccolo (Aczonin)					√					

1. Venkataraman N, Cole AL, Svoboda P, Pohl J, Cole AM: **Cationic polypeptides are required for anti-HIV-1 activity of human vaginal fluid.** *J Immunol* 2005, **175:**7560-7567.
2. Di Quinzio MK, Oliva K, Holdsworth SJ, Ayhan M, Walker SP, Rice GE, Georgiou HM, Permezel M: **Proteomic analysis and characterisation of human cervico-vaginal fluid proteins.** *Aust N Z J Obstet Gynaecol* 2007, **47:**9-15.
3. Dasari S, Pereira L, Reddy AP, Michaels JE, Lu X, Jacob T, Thomas A, Rodland M, Roberts CT, Jr., Gravett MG et al.: **Comprehensive proteomic analysis of human cervical-vaginal fluid.** *J Proteome Res* 2007, **6:**1258-1268.
4. Tang LJ, De SF, Odreman F, Venge P, Piva C, Guaschino S, Garcia RC: **Proteomic analysis of human cervical-vaginal fluids.** *J Proteome Res* 2007, **6:**2874-2883.
5. Shaw JL, Smith CR, Diamandis EP: **Proteomic analysis of human cervico-vaginal fluid.** *J Proteome Res* 2007, **6:**2859-2865.
6. Pereira L, Reddy AP, Jacob T, Thomas A, Schneider KA, Dasari S, Lapidus JA, Lu X, Rodland M, Roberts CT, Jr. et al.: **Identification of novel protein biomarkers of preterm birth in human cervical-vaginal fluid.** *J Proteome Res* 2007, **6:**1269-1276.
7. Andersch-Bjorkman Y, Thomsson KA, Holmen Larsson JM, Ekerhovd E, Hansson GC: **Large scale identification of proteins, mucins, and their O-glycosylation in the endocervical mucus during the menstrual cycle.** *Mol Cell Proteomics* 2007, **6:**708-716.
8. Klein LL, Jonscher KR, Heerwagen MJ, Gibbs RS, McManaman JL: **Shotgun proteomic analysis of vaginal fluid from women in late pregnancy.** *Reprod Sci* 2008, **15:**263-273.
9. Zegels G, Van Raemdonck GA, Coen EP, Tjalma WA, Van Ostade XW: **Comprehensive proteomic analysis of human cervical-vaginal fluid using colposcopy samples.** *Proteome Sci* 2009, **7:**17.
10. Panicker G, Ye Y, Wang D, Unger ER: **Characterization of the Human Cervical Mucous Proteome.** *Clin Proteomics* 2010, **6:**18-28.
