# Supplementary material for: Use of cervicovaginal fluid for the identification of biomarkers for pathologies of the female genital tract
Source: Proteome Sci. 2010 Dec 8;8:63. doi: 10.1186/1477-5956-8-63 (PMC3016264; doi:10.1186/1477-5956-8-63)
Supplement: Additional file 2 — List of proteins identified from CVF and cervical mucus. Swissprot accession number, the protein name, cellular localization and the different biological functions of the protein are given. Yellow highlighted proteins were also identified in human amniotic fluid, green highlighted proteins were identified in human serum and orange highlighted proteins were identified in serum as well as in amniotic fluid. Proteins marked with * are uniquely identified in cervical mucus and not in any other study on human CVF. [file 1477-5956-8-63-S2.PDF]

Additional file 2: list of proteins identified from CVF and cervical mucus.

Swissprot accession number, the protein name, cellular localization and the different biological functions of the protein are given. Yellow highlighted proteins are proteins which were also identified in human amniotic fluid, green highlighted proteins were identified in human serum and orange highlighted proteins were identified in serum as well as in amniotic fluid. Proteins marked with * are uniquely identified in cervical mucus and not in any other study on human CVF.

681073A	Haptoglobin alpha1S	ND	ND
A5YKK6	CCR4-NOT transcription complex, subunit 1 isoform a	Nucleus	Nucleoside, nucleotide and nucleic acid metabolism
A6NHG4	D-dopachrome decarboxylase-like protein	Cytoplasm	Other metabolism
A6NL28	Putative tropomyosin alpha-3 chain-like protein	Cytoplasm	ND
A8K2U0	Alpha-2-macroglobulin-like protein 1 precursor	Extracellular Region	Protein metabolism and modification<br>Signal transduction<br>Immunity and defense
A8MQ03	UPF0574 protein C9orf169	ND	ND
A8MQC9	Uncharacterized protein CLCA4	ND	ND
A9Z1Y9	Thymosin beta-4-like protein 6	Cytoplasm	Cell structure and mobility
BAC01816	Immunoglobulin λ light chain VLJ	ND	Immunity and defense
O00151	PDZ and LIM domain protein 1	Nucleus	Muscle contraction<br>Cell structure and mobility
O00204	Sulfotransferase family cytosolic 2B member 1	Cytoplasm	Lipid, fatty acid and steroid metabolism
O00299	Chloride intracellular channel protein 1	Nucleus	Transport<br>Homeostasis
O00391	Sulfhydryl oxidase 1 precursor	Extracellular Region	Protein metabolism and modification<br>Electron transport
O00555	Voltage-dependent P/Q-type calcium channel subunit alpha-1A	Nucleus	Transport<br>Neuronal activities<br>Muscle contraction
O00584	Ribonuclease T2 precursor	Extracellular Region	Nucleoside, nucleotide and nucleic acid metabolism
O00754	mannosidase, alpha, class 2B, member 1 precursor	Lysosome	Carbohydrate metabolism<br>Protein metabolism and modification

Accession No	Protein Name	Cellular Localisation	Functional Terms
O14745	Ezrin-radixin-moesin-binding phosphoprotein 50	Cytoskeleton	Protein metabolism and modification
			Transport
O15031	plexin-B2 precursor	Membrane	Protein metabolism and modification
			Signal transduction
O15144	Actin-related protein 2/3 complex subunit 2	Cytoskeleton	Protein metabolism and modification
			Cell structure and mobility
			Cell structure and mobility
O15145	Actin-related protein 2/3 complex subunit 3	Cytoskeleton	Cell structure and mobility
O15231	Zinc finger protein 185	Nucleus	Cell proliferation and differentiation
O15260	Surfeit locus protein 4	Cytoplasm	Intracellular protein traffic
			Protein targeting and localization
O15263	Beta-defensin 2 precursor	Extracellular Region	Nucleoside, nucleotide and nucleic acid metabolism
			Immunity and defense
O15393	Transmembrane protease, serine 2 precursor	Membrane	Protein metabolism and modification
O15400	syntaxin 7	Endosome	Intracellular protein traffic
O15457	MutS protein homolog 4	Nucleus	Nucleoside, nucleotide and nucleic acid metabolism
			Developmental processes
O43175	D-3-phosphoglycerate dehydrogenase	Extracellular Region	Amino acid metabolism
O43240	Kallikrein-10 precursor	Extracellular Region	Protein metabolism and modification
O43278	Kunitz-type protease inhibitor 1 precursor	Extracellular Region	Protein metabolism and modification
O43451	Maltase-glucoamylase, intestinal	Membrane	Carbohydrate metabolism
O43497	Voltage-dependent T-type calcium channel subunit alpha-1G	Membrane	Transport
			Neuronal activities
			Muscle contraction
O43707	Actinin, alpha 4	Nucleus	Cell structure and mobility
O43781	Dual-specificity tyrosine-phosphorylation regulated kinase 3	Nucleus	Protein metabolism and modification
O43852	Calumenin precursor	Cytoplasm	Signal transduction
O43866	CD5 antigen-like precursor	Extracellular Region	Transport
			Immunity and defense
			Apoptosis

Accession No	Protein Name	Cellular Localisation	Functional Terms
O60218	Aldo-keto reductase family 1, member B10 (aldose reductase)	Cellular	Nucleoside, nucleotide and nucleic acid metabolism
O60235	Transmembrane protease, serine 11D precursor	Extracellular Region	Nucleoside, nucleotide and nucleic acid metabolism
			Protein metabolism and modification
O60259	Kallikrein-8 precursor	Extracellular Region	Protein metabolism and modification
			Developmental processes
			Cell cycle
			Cell proliferation and differentiation
O60285	NUAK family SNF1-like kinase 1	ND	Protein metabolism and modification
O60346	PH domain leucine-rich repeat	Nucleus	Apoptosis
O60391	Glutamate [NMDA] receptor subunit 3B precursor	Membrane	Signal transduction
			Transport
			Neuronal activities
O60437	Periplakin	Cytoskeleton	Developmental processes
			Cell proliferation and differentiation
O60504	Vinexin	Membrane	Cell structure and mobility
O60664	Mannose-6-phosphate receptor binding protein 1	Endosome	Lipid, fatty acid and steroid metabolism
O60814	Histone H2B type 1-K	Nucleus	Nucleoside, nucleotide and nucleic acid metabolism
			Immunity and defense
O75015	Fc-gamma receptor IIIb	Membrane	Signal transduction
			Immunity and defense
O75083	WD repeat protein 1	Cytoskeleton	Sensory perception
			Cell structure and mobility
O75131	Copine-3	Cytoplasm	Intracellular protein traffic
O75223	Protein C7orf24	ND	ND
O75351	Vacuolar protein sorting-associating protein 4B	Membrane	Protein metabolism and modification
			Intracellular protein traffic
			Protein targeting and localization
			Cell structure and mobility
O75367	Core histone macro-H2A.1	Nucleus	Nucleoside, nucleotide and nucleic acid metabolism
O75369	Filamin-B	Membrane	Cell structure and mobility

Accession No	Protein Name	Cellular Localisation	Functional Terms
O75448	Thyroid hormone receptor-associated protein complex 100 kDa component	Nucleus	Nucleoside, nucleotide and nucleic acid metabolism
O75594	Peptidoglycan recognition protein precursor	Extracellular Region	Carbohydrate metabolism<br>Immunity and defense<br>Apoptosis
O75629	Protein CREG1 precursor	Nucleus	Nucleoside, nucleotide and nucleic acid metabolism
O75882	Attractin precursor	Extracellular Region	Cell adhesion<br>Immunity and defense
O94823	Probable phospholipid-transporting ATPase VB	Membrane	Lipid, fatty acid and steroid metabolism<br>Transport
O94919	Endonuclease domain-containing 1 protein precursor	Extracellular Region	Nucleoside, nucleotide and nucleic acid metabolism
O95171	Sciellin	Cytoskeleton	Developmental processes
O95274	Ly6/PLAUR domain-containing protein 3 precursor	Membrane	Cell adhesion<br>Oncogenesis<br>Cell structure and mobility
O95361	Tripartite motif-containing protein 16	Cytoplasm	Protein metabolism and modification
O95831	Programmed cell death protein 8, mitochondrial precursor	Mitochondrion	Apoptosis
P00338	L-lactate dehydrogenase A chain	Cytoplasm	Carbohydrate metabolism
P00390	Glutathione reductase, mitochondrial precursor	Cytoplasm	Nucleoside, nucleotide and nucleic acid metabolism<br>Electron transport
P00441	Superoxide dismutase [Cu-Zn]	Extracellular Region	Immunity and defense
P00450	Ceruloplasmin precursor	Extracellular Region	Homeostasis
P00491	Purine nucleoside phosphorylase	Cytoplasm	Nucleoside, nucleotide and nucleic acid metabolism
P00492	Hypoxanthine-guanine phosphoribosyltransferase	Cytoplasm	Nucleoside, nucleotide and nucleic acid metabolism
P00558	Phosphoglycerate kinase 1	Cytoplasm	Carbohydrate metabolism
P00738	Haptoglobin	Extracellular Region	Protein metabolism and modification<br>Immunity and defense<br>Blood circulation and gas exchange
P00747	Plasminogen precursor	Extracellular Region	Lipid, fatty acid and steroid metabolism<br>Protein metabolism and modification<br>Transport

Accession No	Protein Name	Cellular Localisation	Functional Terms
			Immunity and defense
			Apoptosis
			Developmental processes
			Blood circulation and gas exchange
			Cell proliferation and differentiation
P00748	Coagulation factor XII precursor	Extracellular Region	Protein metabolism and modification
			Immunity and defense
			Cell structure and mobility
P00751	Complement factor B precursor	Extracellular Region	Protein metabolism and modification
			Immunity and defense
P00915	Carbonic anhydrase 1	Cytoplasm	Nucleoside, nucleotide and nucleic acid metabolism
P00918	Carbonic anhydrase 2	Cytoplasm	Nucleoside, nucleotide and nucleic acid metabolism
P01008	Antithrombin-III precursor	Extracellular Region	Protein metabolism and modification
P01009	Alpha-1-antitrypsin precursor	Extracellular Region	Protein metabolism and modification
P01011	Alpha-1-antichymotrypsin precursor	Extracellular Region	Protein metabolism and modification
P01019	Angiotensinogen precursor [Contains: Angiotensin I	Extracellular Region	Protein metabolism and modification
P01023	Alpha-2-macroglobulin precursor	Extracellular Region	Protein metabolism and modification
			Signal transduction
			Immunity and defense
P01024	Complement component 3	Extracellular Region	Immunity and defense
P01028	Complement C4 precursor	Extracellular Region	Immunity and defense
P01033	Metalloproteinase inhibitor 1 precursor	Extracellular Region	Protein metabolism and modification
			Developmental processes
P01034	Cystatin-C	Extracellular Region	Protein metabolism and modification
P01040	Cystatin A (stefin A)	Cytoskeleton	Protein metabolism and modification
P01042	Kininogen	Extracellular Region	Immunity and defense
			Blood circulation and gas exchange
P01591	Immunoglobulin J chain	Extracellular Region	Immunity and defense
P01593	Ig kappa chain V-I	Extracellular Region	Immunity and defense
P01597	Ig kappa chain V-I region DEE	Extracellular Region	Immunity and defense

Accession No	Protein Name	Cellular Localisation	Functional Terms
P01605	Ig kappa chain V-I region Lay	Extracellular Region	Immunity and defense
P01606	Ig kappa chain V-I region OU	Extracellular Region	Immunity and defense
P01616	Ig kappa chain V-II region MIL	Extracellular Region	Immunity and defense
P01617	Ig kappa chain V-II region TEW	Extracellular Region	Immunity and defense
P01620	Ig kappa chain V-III region SIE	Extracellular Region	Immunity and defense
P01625	Ig kappa chain V-IV region Len	Extracellular Region	Immunity and defense
P01701	Ig lambda chain V-I region NEW	Extracellular Region	Immunity and defense
P01703	Ig lambda chain V-I region NEWM	Extracellular Region	Immunity and defense
P01708	Ig lambda chain V-II region BUR	Extracellular Region	Immunity and defense
P01714	Ig lambda chain V-III region SH	Extracellular Region	Immunity and defense
P01743	Ig heavy chain V-I region HG3 precursor	Extracellular Region	Immunity and defense
P01766	Ig heavy chain V-III region BRO	Extracellular Region	Immunity and defense
P01768	Ig heavy chain V-III region CAM	Extracellular Region	Immunity and defense
P01771	Ig heavy chain V-III region HIL	Extracellular Region	Immunity and defense
P01772	Ig heavy chain V-III region KOL	Extracellular Region	Immunity and defense
P01833	Polymeric immunoglobulin receptor	Membrane	Intracellular protein traffic
			Immunity and defense
P01834	Ig kappa chain C region	Extracellular Region	Immunity and defense
P01842	Ig lambda chain C regions	Membrane	Immunity and defense
P01857	Ig gamma-1 chain C region	Membrane	Immunity and defense
P01859	Ig gamma-2 chain C region	Membrane	Immunity and defense
P01860	Ig gamma-3 chain C region	Membrane	Immunity and defense
P01861	Ig gamma-4 chain C region	Membrane	Immunity and defense
P01871	Ig mu chain C region	Membrane	Immunity and defense
P01876	Ig alpha-1 chain C region	Extracellular Region	Immunity and defense
P01877	Ig alpha-2 chain C region	Membrane	Immunity and defense
P02042	Hemoglobin subunit delta	Cytoplasm	Transport
			Blood circulation and gas exchange
P02100	Hemoglobin subunit epsilon	Cytoplasm	Transport
			Blood circulation and gas exchange

Accession No	Protein Name	Cellular Localisation	Functional Terms
P02511	Alpha-crystallin B chain	Cytoplasm	Muscle contraction
			Sensory perception
P02545	Lamin-A/C	Nucleus	Cell structure and mobility
P02647	Apolipoprotein A1	Extracellular Region	Lipid, fatty acid and steroid metabolism
			Transport
P02652	Apolipoprotein A-II precursor	Extracellular Region	Lipid, fatty acid and steroid metabolism
			Transport
P02671	Fibrinogen alpha chain precursor	Extracellular Region	Immunity and defense
			Blood circulation and gas exchange
			Cell proliferation and differentiation
P02675	Fibrinogen beta chain precursor	Extracellular Region	Immunity and defense
			Blood circulation and gas exchange
			Cell proliferation and differentiation
P02679	Fibrinogen gamma chain	Extracellular Region	Immunity and defense
			Blood circulation and gas exchange
			Cell proliferation and differentiation
P02749	Apolipoprotein H (beta-2-glycoprotein I)	Extracellular Region	Immunity and defense
P02750	Leucine-rich alpha-2-glycoprotein precursor	Extracellular Region	Signal transduction
			Developmental processes
P02751	Fibronectin precursor	Extracellular Region	Signal transduction
			Cell adhesion
			Intracellular protein traffic
			Cell structure and mobility
P02760	AMBP protein precursor [Contains: Alpha-1-microglobulin	Extracellular Region	Protein metabolism and modification
P02763	Alpha-1-acid glycoprotein 1	Extracellular Region	Immunity and defense
P02765	Alpha-2-HS-glycoprotein	Extracellular Region	Protein metabolism and modification
			Immunity and defense
			Developmental processes
P02766	Transthyretin	Extracellular Region	Transport
P02768	Serum albumin precursor	Extracellular Region	Transport

Accession No	Protein Name	Cellular Localisation	Functional Terms
P02774	Vitamin D-binding protein precursor	Extracellular Region	Coenzyme and prosthetic group metabolism
			Transport
P02787	Serotransferrin	Extracellular Region	Transport
			Miscellaneous
P02788	Lactotransferrin	Extracellular Region	Transport
			Miscellaneous
P02790	Hemopexin	Extracellular Region	Coenzyme and prosthetic group metabolism
			Transport
P02792	Ferritin light chain	Intracellular	Transport
			Homeostasis
P28066	Proteasome subunit alpha type 5	Cytoplasm	Protein metabolism and modification
			Cell Cycle
P03973	Antileukoproteinase 1 precursor	Extracellular Region	Protein metabolism and modification
			Immunity and defense
P04003	C4b-binding protein alpha chain precursor	Extracellular Region	Immunity and defense
P04004	Vitronectin	Extracellular Region	Signal transduction
			Cell adhesion
P04040	Catalase	Membrane	Electron transport
			Immunity and defense
P04075	Fructose-bisphosphate aldolase A	Cytoskeleton	Carbohydrate metabolism
P04080	Cystatin B	Extracellular Region	Protein metabolism and modification
P04083	Annexin A1	Cytoskeleton	Lipid, fatty acid and steroid metabolism
			Signal transduction
			Cell structure and mobility
P04114	Apolipoprotein B-100 precursor	Extracellular Region	Lipid, fatty acid and steroid metabolism
			Transport
P04196	Histidine-rich glycoprotein precursor	Extracellular Region	Immunity and defense
P04207	Ig kappa chain V-III region CLL [Precursor]	Extracellular Region	Immunity and defense
P04208	Ig lambda chain V-I region WAH	Extracellular Region	Immunity and defense
P04217	Alpha-1-B glycoprotein	Extracellular Region	Signal transduction

Accession No	Protein Name	Cellular Localisation	Functional Terms
			Immunity and defense
P04279	Semenogelin-1	Extracellular Region	Developmental processes
P04406	Glyceraldehyde-3-phosphate dehydrogenase, liver	Cytoplasm	Carbohydrate metabolism
P04433	Ig kappa chain V-III region VG precursor	Extracellular Region	Immunity and defense
P04792	Heat-shock protein beta-1	Cytoskeleton	Protein metabolism and modification
			Immunity and defense
P05089	Arginase-1	Cytoplasm	Amino acid metabolism
P05090	Apolipoprotein D precursor	Extracellular Region	Coenzyme and prosthetic group metabolism
			Transport
P05107	Integrin beta-2 precursor	Membrane	Signal transduction
			Cell adhesion
P05109	Calgranulin A (S100A8)	Extracellular Region	Immunity and defense
			Cell structure and mobility
P05154	Plasma serine protease inhibitor precursor	Extracellular Region	Protein metabolism and modification
P05155	Plasma protease C1 inhibitor precursor	Extracellular Region	Protein metabolism and modification
P05164	Myeloperoxidase precursor	Lysosome	Immunity and defense
P05204	Non-histone chromosomal protein HMG-17	Nucleus	Nucleoside, nucleotide and nucleic acid metabolism
			Cell cycle
P05386	60S acidic ribosomal protein P1	Cytoplasm	Protein metabolism and modification
P05387	60S acidic ribosomal protein P2	Cytoplasm	Protein metabolism and modification
P05388	60S acidic ribosomal protein P10	Cytoplasm	Protein metabolism and modification
P06312	Ig kappa chain V-IV region precursor	Extracellular Region	Immunity and defense
P06396	Gelsolin	Extracellular Region	Cell structure and mobility
P06702	Calgranulin B (S100A9)	Extracellular Region	Signal transduction
			Immunity and defense
P06731	Carcinoembryonic antigen-related cell adhesion molecule 5 precursor	Membrane	Signal transduction
			Cell adhesion
P06733	Alpha-enolase	Nucleus	Carbohydrate metabolism
P06744	Glucose-6-phosphate isomerase	Extracellular Region	Carbohydrate metabolism
P06753	Tropomyosin 3	Cytoskeleton	Muscle contraction

Accession No	Protein Name	Cellular Localisation	Functional Terms
			Developmental processes
			Cell structure and mobility
P07108	Acyl-CoA binding protein	ND	Lipid, fatty acid and steroid metabolism
P07237	Procollagen-proline, 2-oxoglutarate 4-dioxygenase (proline 4-hydroxylase), beta polypeptide; Protein disulfide-isomerase precursor	Extracellular Region	Protein metabolism and modification
P07305	Histone H1.0	Cytoskeleton	Nucleoside, nucleotide and nucleic acid metabolism
P07339	Cathepsin D precursor	Extracellular Region	Protein metabolism and modification
P07355	Annexin A2	Membrane	Intracellular protein traffic
			Developmental processes
			Cell structure and mobility
P07384	Calpain-1 catalytic subunit	Cytoplasm	Protein metabolism and modification
			Signal transduction
P07476	Involucrin	Cytoskeleton	Cell proliferation and differentiation
			Cell structure and mobility
P07686	Beta-hexosaminidase beta chain precursor	Lysosome	Carbohydrate metabolism
			Lipid, fatty acid and steroid metabolism
P07737	Profilin 1	Cytoskeleton	Nucleoside, nucleotide and nucleic acid metabolism
			Cell adhesion
			Developmental processes
P07858	Cathepsin B	Intracellular	Protein metabolism and modification
			Oncogenesis
P07900	Heat shock protein HSP 90-alpha 2	Cytoplasm	Protein metabolism and modification
			Immunity and defense
P07911	Uromodulin precursor	Membrane	Immunity and defense
			Cell proliferation and differentiation
P07919	Ubiquinol-cytochrome c reductase complex 11 kDa protein, mitochondrial precursor	Envelope	Electron transport
P07948	Tyrosine-protein kinase Lyn	Membrane	Carbohydrate metabolism
			Protein metabolism and modification
			Signal transduction
			Transport

Accession No	Protein Name	Cellular Localisation	Functional Terms
			Immunity and defense<br>Developmental processes<br>Cell cycle<br>Cell proliferation and differentiation<br>Oncogenesis
P07951	Tropomyosin beta chain	Cytoskeleton	Muscle contraction<br>Developmental processes<br>Cell structure and mobility
P07998	Ribonuclease pancreatic precursor	Extracellular Region	Nucleoside, nucleotide and nucleic acid metabolism
P08107	Heat shock 70 kDa protein 1	Mitochondrion	Protein metabolism and modification<br>Immunity and defense
P08123	Collagen alpha 2 T	Extracellular Region	Cell adhesion<br>Cell structure and mobility
P08174	Complement decay-accelerating factor precursor	Membrane	Immunity and defense
P08236	Beta-glucuronidase precursor	Lysosome	Carbohydrate metabolism
P08238	Heat shock protein HSP 90-beta	Cytoplasm	Protein metabolism and modification<br>Immunity and defense
P08246	Leukocyte elastase precursor	Extracellular Region	Protein metabolism and modification<br>Immunity and defense
P08311	Cathepsin G	Lysosome	Protein metabolism and modification<br>Immunity and defense<br>Apoptosis
P08603	Complement factor H	Extracellular Region	Immunity and defense
P08670	Vimentin	Cytoskeleton	Developmental processes<br>Cell structure and mobility
P08697	Alpha-2-antiplasmin precursor	Extracellular Region	Protein metabolism and modification
P08708	40S ribosomal protein S17	Cytoplasm	Protein metabolism and modification
P08758	Annexin A5; Calphobindin I	Cytoplasm	Lipid, fatty acid and steroid metabolism
P08833	Insulin-like growth factor binding protein 1	Extracellular Region	Signal transduction<br>Homeostasis

Accession No	Protein Name	Cellular Localisation	Functional Terms
P09211	Glutathione S-transferase P	Cytoplasm	Immunity and defense
P09429	High mobility group protein B1	Nucleus	Nucleoside, nucleotide and nucleic acid metabolism
			Signal transduction
			Apoptosis
			Developmental processes
			Cell proliferation and differentiation
P09466	Glycodelin precursor	Extracellular Region	Developmental processes
P09493	Tropomyosin alpha-1 chain	Cytoskeleton	Muscle contraction
			Developmental processes
			Cell structure and mobility
P09497	Clathrin light chain B	Golgi Apparatus	Intracellular protein traffic
P09525	annexin IV	Cytoplasm	Lipid, fatty acid and steroid metabolism
P09651	Heterogeneous nuclear ribonucleoprotein A1	Nucleus	Nucleoside, nucleotide and nucleic acid metabolism
P09668	Cathepsin H precursor	Lysosome	Protein metabolism and modification
P09758	Tumor-associated calcium signal transducer 2 precursor	Membrane	Signal transduction
			Cell proliferation and differentiation
P09960	Leukotriene A-4 hydrolase	Cytoplasm	Lipid, fatty acid and steroid metabolism
			Protein metabolism and modification
			Immunity and defense
P09972	Fructose-bisphosphate aldolase C	Cytoskeleton	Carbohydrate metabolism
P0C0L5	Complement C4-B precursor	Extracellular Region	Immunity and defense
P0C0S5	Histone H2A.Z (H2A/z).	Nucleus	Nucleoside, nucleotide and nucleic acid metabolism
P0C0S8	Histone H2A type 1	Nucleus	Nucleoside, nucleotide and nucleic acid metabolism
P0C869	Cytosolic phospholipase A2 beta	Cytoplasm	Lipid, fatty acid and steroid metabolism
			Immunity and defense
			Cell proliferation and differentiation
P10153	Nonsecretory ribonuclease precursor	Extracellular Region	Nucleoside, nucleotide and nucleic acid metabolism
P10155	60 kDa SS-A/Ro ribonucleoprotein	Intracellular	Nucleoside, nucleotide and nucleic acid metabolism
			Protein metabolism and modification
P10412	Histone H1.4	Intracellular	Nucleoside, nucleotide and nucleic acid metabolism

Accession No	Protein Name	Cellular Localisation	Functional Terms
P10599	Thioredoxin	Cytoplasm	Electron transport
			Signal transduction
			Immunity and defense
			Apoptosis
			Cell proliferation and differentiation
			Other metabolism
P10606	Cytochrome c oxidase subunit 5B, mitochondrial precursor	Mitochondrion	Electron transport
P10619	Lysosomal protective protein precursor	Lysosome	Protein metabolism and modification
			Signal transduction
			Intracellular protein traffic
P10909	Clusterin precursor	Extracellular Region	Apoptosis
P11021	78 kDa glucose-regulated protein	Cell Surface	Protein metabolism and modification
P11142	Heat shock 70kDa protein 8	Cell Surface	Protein metabolism and modification
			Immunity and defense
P11216	Glycogen phosphorylase, brain form	Cytoplasm	Carbohydrate metabolism
P11413	glucose-6-phosphate dehydrogenase isoform a	Cytoplasm	Carbohydrate metabolism
P12036	Neurofilament heavy polypeptide	Cytoskeleton	Cell structure and mobility
P12273	Prolactin-inducible protein precursor	Extracellular Region	ND
P12429	Annexin A3	Cytoplasm	Lipid, fatty acid and steroid metabolism
P12724	Eosinophil cationic protein precursor	Extracellular Region	Nucleoside, nucleotide and nucleic acid metabolism
P12814	Alpha-actinin-1	Membrane	Cell structure and mobility
P12830	E-cadherin	Cytoskeleton	Signal transduction
			Cell adhesion
P13611	Versican core protein precursor	Extracellular Region	Signal transduction
			Cell proliferation and differentiation
			Cell structure and mobility
P13639	Elongation factor 2	Cytoplasm	Protein metabolism and modification
P13667	Protein disulfide-isomerase A4 precursor	Cytoplasm	Protein metabolism and modification
P13671	Complement component C6 precursor	Extracellular Region	Immunity and defense
P13796	Lymphocyte cytosolic protein 1 (L-plastin)	Cytoplasm	Cell structure and mobility

Accession No	Protein Name	Cellular Localisation	Functional Terms
P13797	Plastin-3	Cytoskeleton	Cell structure and mobility
P13929	Beta-enolase	Cytoplasm	Carbohydrate metabolism
P13987	CD59 glycoprotein precursor	Membrane	Immunity and defense
P14136	Glial fibrillary acidic protein	Cytoskeleton	Cell structure and mobility
P14174	Macrophage migration inhibitory factor	Extracellular Region	Immunity and defense
P14314	Glucosidase 2 subunit beta precursor	Intracellular	Carbohydrate metabolism
			Protein metabolism and modification
P14317	Hematopoietic lineage cell-specific protein	Nucleus	Nucleoside, nucleotide and nucleic acid metabolism
P14384	Carboxypeptidase M	Membrane	Protein metabolism and modification
P14618	Pyruvate kinase isozymes M1/M2	Cytoplasm	Carbohydrate metabolism
P14625	Endoplasmin precursor	Membrane	Protein metabolism and modification
			Immunity and defense
P14780	Matrix metalloproteinase-9 precursor	Extracellular Region	Protein metabolism and modification
P14854	Cytochrome c oxidase subunit VIb isoform 1	Mitochondrion	Electron transport
P14923	Desmoplakin-3	Membrane	Signal transduction
P15056	B-Raf proto-oncogene serine/threonine-protein kinase	Membrane	Signal transduction
			Apoptosis
			Cell proliferation and differentiation
			Oncogenesis
P15104	Glutamine synthetase	Mitochondrion	Amino acid metabolism
			Other metabolism
P15153	Ras-related C3 botulinum toxin substrate 2 precursor	Membrane	Signal transduction
			Cell structure and mobility
P15259	Phosphoglycerate mutase 2	ND	Carbohydrate metabolism
P15289	Arylsulfatase A precursor	Extracellular Region	Lipid, fatty acid and steroid metabolism
			Other metabolism
P15309	Prostatic acid phosphatase precursor	Extracellular Region	Nucleoside, nucleotide and nucleic acid metabolism
			Other metabolism
P15311	Ezrin	Membrane	Cell structure and mobility
P15538	cytochrome P450, family 11, subfamily B, polypeptide 1 isoform 2 precursor	Mitochondrion	Lipid, fatty acid and steroid metabolism

Accession No	Protein Name	Cellular Localisation	Functional Terms
			Electron transport
P15924	Desmoplakin	Cytoskeleton	Protein metabolism and modification
			Developmental processes
			Cell proliferation and differentiation
P16035	Metalloproteinase inhibitor 2	Extracellular Region	Protein metabolism and modification
P16401	Histone H1.5 (Histone H1a)	Intracellular	Nucleoside, nucleotide and nucleic acid metabolism
P16402	Histone H1.3	Intracellular	Nucleoside, nucleotide and nucleic acid metabolism
P16403	Histone H1.2	Intracellular	Nucleoside, nucleotide and nucleic acid metabolism
P16870	Carboxipeptidase E	Membrane	Protein metabolism and modification
P17213	bactericidal/permeability-increasing protein precursor	Membrane	Immunity and defense
P17858	liver phosphofructokinase isoform b	Cytoplasm	Carbohydrate metabolism
P17900	Ganglioside GM2 activator precursor	Lysosome	Lipid, fatty acid and steroid metabolism
P17931	Galectin-3	Nucleus	Cell adhesion
			Immunity and defense
			Apoptosis
P18054	Arachidonate 12-lipoxygenase, 12S-type	Membrane	Lipid, fatty acid and steroid metabolism
			Immunity and defense
P18136	Ig kappa chain V-III region HIC [Precursor]	Extracellular Region	Immunity and defense
P18206	Vinculin	Membrane	Cell structure and mobility
P18510	Interleukin 1 receptor antagonist protein	Extracellular Region	Signal transduction
			Immunity and defense
P18621	60S ribosomal protein L17 (L23) isoform 5	Cytoplasm	Protein metabolism and modification
P18669	Phosphoglycerate mutase 1	Cytoplasm	Carbohydrate metabolism
P18858	DNA ligase 1	Nucleus	Nucleoside, nucleotide and nucleic acid metabolism
			Cell cycle
P18859	ATP synthase, H+ transportine, mitochondrial FO complex,	Envelope	Electron transport
			Transport
P19105	Myosin regulatory light chain 2, nonsarcomeric	Cytoskeleton	Muscle contraction
			Developmental processes
			Cell structure and mobility

Accession No	Protein Name	Cellular Localisation	Functional Terms
P19447	TFIIH basal transcription factor complex helicase XPB subunit	Nucleus	Nucleoside, nucleotide and nucleic acid metabolism
P19652	Alpha-1-acid glycoprotein 2 precursor	Extracellular Region	Immunity and defense
P19957	Elafin precursor	Extracellular Region	Protein metabolism and modification
			Immunity and defense
P19961	Alpha-amylase 2B precursor	Extracellular Region	Carbohydrate metabolism
P20020	Plasma membrane calcium-transporting ATPase1	Membrane	Transport
			Homeostasis
P20061	Transcobalamin-1 precursor	Extracellular Region	Coenzyme and prosthetic group metabolism
			Transport
P20160	Azurocidin 1 (cationic antimicrobial protein 37)	Extracellular Region	Protein metabolism and modification
			Immunity and defense
P20670	Histone H2A.o	Nucleus	Nucleoside, nucleotide and nucleic acid metabolism
P20700	Lamin-B1	Envelope	Cell structure and mobility
P20810	Calpastatin (Calpain inhibitor) (Sperm BS-17 component)	Cytoplasm	Protein metabolism and modification
P20908	Collagen alpha-1(V) chain precursor	Extracellular Region	Developmental processes
P20930	Filaggrin	Cytoskeleton	Protein metabolism and modification
			Developmental processes
			Cell structure and mobility
P21128	Placental protein 11 precursor	Extracellular Region	Other metabolism
P21333	Filamin-A	Cytoskeleton	Cell structure and mobility
P21817	Ryanodine receptor 1	Membrane	Signal transduction
			Transport
			Muscle contraction
P22090	40S ribosomal protein S4, Y isoform 1	Cytoplasm	Protein metabolism and modification
P22314	Ubiquitin-activating enzyme E1	Cytoplasm	Protein metabolism and modification
P22528	Cornifin B	Cytoskeleton	Protein metabolism and modification
			Developmental processes
			Cell proliferation and differentiation
P22531	Small proline-rich protein 2E	Cytoskeleton	Developmental processes
			Cell proliferation and differentiation

Accession No	Protein Name	Cellular Localisation	Functional Terms
P22532	Small proline-rich protein 2D	Cytoskeleton	Developmental processes
			Cell proliferation and differentiation
P22626	Heterogeneous nuclear ribonucleoprotein A2/B1	Nucleus	Nucleoside, nucleotide and nucleic acid metabolism
P22735	Protein-glutamine gamma-glutamyltransferase K	Cytoskeleton	Protein metabolism and modification
			Developmental processes
P22894	Neutrophil collagenase precursor	Extracellular Region	Protein metabolism and modification
P23083	Ig heavy chain V-I region V35 precursor	Extracellular Region	Immunity and defense
P23142	Fibulin-1 precursor	Extracellular Region	Signal transduction
			Sensory perception
			Developmental processes
P23246	Splicing factor, proline-and gluatmine-rich	Nucleus	Nucleoside, nucleotide and nucleic acid metabolism
P23284	peptidylprolyl isomerase B precursor	Endoplasmic Reticulum	Protein metabolism and modification
			Intracellular protein traffic
			Immunity and defense
P23396	40S ribosomal protein S3	Cytoplasm	Protein metabolism and modification
P23526	Adenosylhomocysteinase	Cytoplasm	Nucleoside, nucleotide and nucleic acid metabolism
P23528	cofilin-1	Nucleus	Cell structure and mobility
P23786	Carnitine O-palmitoyltransferase 2, mitochondrial	Envelope	Amino acid metabolism
			Lipid, fatty acid and steroid metabolism
P24158	Myeloblastin precursor	Cellular	Protein metabolism and modification
P25311	Alpha-2-glycoprotein 1, zinc	Extracellular Region	Immunity and defense
P25685	DnaJ homolog subfamily B member 1	Nucleus	Protein metabolism and modification
P25774	Cathepsin S precursor	Extracellular Region	Protein metabolism and modification
			Immunity and defense
P25789	Proteasome subunit alpha type 4	Cytoplasm	Protein metabolism and modification
P25815	S100 calcium binding protein P	Nucleus	Cell structure and mobility
P26038	Moesin	Cytoskeleton	Cell structure and mobility
P26373	60S ribosomal protein L13	Cytoplasm	Protein metabolism and modification
P26447	S100 calcium binding protein A4 (calcium protein, calvasculin, metastasin, murine placental homolog)	ND	Immunity and defense

Accession No	Protein Name	Cellular Localisation	Functional Terms
P26641	Elongation factor 1-gamma	Cytoplasm	Protein metabolism and modification
P27482	Calmodulin-like protein 3	ND	Signal transduction
			Cell cycle
			Cell proliferation and differentiation
P27487	Dipeptidyl peptidase 4	Cell Surface	Protein metabolism and modification
			Signal transduction
			Immunity and defense
P27797	Calreticulin precursor	Endoplasmic Reticulum	Protein metabolism and modification
P27816	Microtubule-associated protein 4	Cytoskeleton	Apoptosis
			Cell structure and mobility
P27824	Calnexin precursor	Endoplasmic Reticulum	Protein metabolism and modification
			Intracellular protein traffic
P27918	Properdin precursor	Extracellular Region	Immunity and defense
P28001	Histone H2A.a	Nucleus	Nucleoside, nucleotide and nucleic acid metabolism
P28799	Granulins precursor	Extracellular Region	Signal transduction
P29034	Protein S100-A2	Nucleus	Oncogenesis
P29218	Inositol monophosphatase	Cytoplasm	Lipid, fatty acid and steroid metabolism
P29373	Cellular retinoic acid-binding protein 2	Cytoplasm	Lipid, fatty acid and steroid metabolism
			Coenzyme and prosthetic group metabolism
			Signal transduction
			Transport
			Developmental processes
P29401	Transketolase	Cytoplasm	Carbohydrate metabolism
			Coenzyme and prosthetic group metabolism
P29508	Squamous cell carcinoma antigen 1 (SCCA-1); Serpin B3	Extracellular Region	Protein metabolism and modification
P29590	Probable transcription factor PML	Nucleus	Protein metabolism and modification
P30041	Peroxiredoxin-6	Cytoplasm	Immunity and defense
P30043	Flavin reductase	Cytoplasm	Other metabolism
P30046	D-dopachrome tautomerase	Cytoplasm	Immunity and defense
P30050	60S ribosomal protein L12	Cytoplasm	Protein metabolism and modification

Accession No	Protein Name	Cellular Localisation	Functional Terms
P30086	Phosphatidylethanolamine-binding protein; Prostatic binding protein; neuropolypeptide h3	Cytoplasm	Signal transduction
P30101	Glucose regulated protein, 58kDa; protein disulfide-isomerase A3	Cytoplasm	Protein metabolism and modification
P30153	Serine/threonine-protein phosphatase 2A 65 kDa regulatory subunit A	Cytoskeleton	Lipid, fatty acid and steroid metabolism
			Nucleoside, nucleotide and nucleic acid metabolism
			Protein metabolism and modification
			Signal transduction
			Cell adhesion
			Apoptosis
			Developmental processes
			Cell proliferation and differentiation
			Other metabolism
P30456	HLA class I histocompatibility antigen, A-43 alpha chain precursor	Membrane	Immunity and defense
			Immunity and defense
P30475	HLA class I histocompatibility antigen, B-39 alpha chain	Membrane	Immunity and defense
P30740	Monocyte/neutrophil elastase inhibitor; Serine (or cysteine) proteinase inhibitor, clade B (ovalbumin), member 1	Cytoplasm	Protein metabolism and modification
P31146	Coronin, actin binding protein, 1A	Cytoskeleton	Signal transduction
			Intracellular protein traffic
			Transport
			Cell cycle
			Cell proliferation and differentiation
			Cell structure and mobility
P31151	S100 calcium-binding protein A7 (psoriasin)	Extracellular Region	Immunity and defense
			Developmental processes
			Cell proliferation and differentiation
P31689	DnaJ homolog subfamily A member 1	Membrane	Protein metabolism and modification
			Immunity and defense
P31930	Ubiquinol-cytochrome-c reductase complex core protein I, mitochondrial precursor	Envelope	Electron transport
P31942	Heterogeneous nuclear ribonucleoprotein H3	Nucleus	Nucleoside, nucleotide and nucleic acid metabolism

Accession No	Protein Name	Cellular Localisation	Functional Terms
P31944	Caspase-14 precursor	Nucleus	Protein metabolism and modification
			Apoptosis
P31946	14-3-3 protein beta/alpha	Cytoplasm	Signal transduction
			Protein targeting and localization
			Cell cycle
P31947	Stratifin; 14-3-3 protein sigma	Extracellular Region	Signal transduction
			Protein targeting and localization
			Cell cycle
P31948	Stress-induced-phosphoprotein 1	Nucleus	Immunity and defense
P31949	S100 calcium-binding protein A11 (calgizzarine)	Nucleus	Nucleoside, nucleotide and nucleic acid metabolism
			Cell cycle
			Oncogenesis
P31997	Carcinoembryonic antigen-related cell adhesion molecule 8 precursor	Extracellular Region	Signal transduction
			Cell adhesion
P32119	Peroxiredoxin-2	Cytoplasm	Immunity and defense
P32243	Homeobox protein OTX2	Nucleus	Nucleoside, nucleotide and nucleic acid metabolism
			Developmental processes
P32320	Cytidine deaminase	Extracellular Region	Nucleoside, nucleotide and nucleic acid metabolism
P32926	Desmoglein-3	Membrane	Signal transduction
			Cell adhesion
P33241	Lymphocyte-specific protein 1	Cytoskeleton	Signal transduction
			Immunity and defense
			Cell structure and mobility
P33778	Histone H2B.f	Intracellular	Other metabolism
P34059	N-acetylgalactosamine-6-sulfatase precursor	Lysosome	Carbohydrate metabolism
			Other metabolism
P34932	Heat shock 70 kDa protein 4	Cytoplasm	Protein metabolism and modification
P35221	Catenin-alpha-1	Cytoskeleton	Cell structure and mobility
P35237	Serpin B6 (Placental thrombin inhibitor)	Cytoplasm	Protein metabolism and modification
P35268	60S ribosomal protein L22	Cytoplasm	Protein metabolism and modification

Accession No	Protein Name	Cellular Localisation	Functional Terms
P35321	Small-proline rich protein 1A; Cornifin A	Cytoskeleton	Protein metabolism and modification
			Developmental processes
			Cell proliferation and differentiation
P35325	Small proline-rich protein 2B	Cytoskeleton	Developmental processes
			Cell proliferation and differentiation
P35326	Small proline-rich protein 2A	Cytoskeleton	Developmental processes
			Cell proliferation and differentiation
P35555	Fibrillin-1 precursor	Extracellular Region	Signal transduction
			Sensory perception
			Developmental processes
P35579	Myosin-9	Cytoskeleton	Developmental processes
			Cell structure and mobility
P35658	Nuclear pore complex protein Nup214	Envelope	Intracellular protein traffic
P35754	Glutaredoxin-1	Cytoplasm	Other metabolism
P35789	Zinc finger protein 93	Nucleus	Nucleoside, nucleotide and nucleic acid metabolism
			Cell proliferation and differentiation
P36952	Maspin precursor; Serpin B5 precursor	Extracellular Region	Protein metabolism and modification
P36957	Dihydrolipoyllysine-residue succinyltransferase component	Mitochondrion	Other metabolism
P37802	Transgelin-2	Nucleus	Muscle contraction
P37837	Transaldolase	Cytoplasm	Carbohydrate metabolism
P38159	RNA binding motif protein, X-linked-like 1	Nucleus	Nucleoside, nucleotide and nucleic acid metabolism
			Developmental processes
P38646	Heat shock 70 kDa protein 9	Cell Surface	Protein metabolism and modification
			Immunity and defense
P39019	40S ribosomal protein S19	Nucleus	Protein metabolism and modification
P39023	60S ribosomal protein L3	Nucleus	Protein metabolism and modification
P39060	Type XVIII collagen long variant	Extracellular Region	Developmental processes
P40121	Macrophage-capping protein	Cytoskeleton	Cell structure and mobility
P40926	Malate dehydrogenase, mitochondrial precursor	Mitochondrion	Carbohydrate metabolism
P41439	folate receptor 3 precursor	Membrane	Coenzyme and prosthetic group metabolism

Accession No	Protein Name	Cellular Localisation	Functional Terms
			Transport
P42677	40S ribosomal protein S27	Cytoplasm	Protein metabolism and modification
P42766	60S ribosomal protein L35	Nucleus	Protein metabolism and modification
P43490	Nicotinamide phosphoribosyltransferase	Cytoplasm	Signal transduction
			Immunity and defense
P43652	Afamin precursor	Extracellular Region	Transport
P46108	Proto-oncogene c-crk	Endosome	Nucleoside, nucleotide and nucleic acid metabolism
			Signal transduction
			Cell structure and mobility
P46776	60S ribosomal protein L27a	Cytoplasm	Protein metabolism and modification
P46778	60S ribosomal protein L21	Cytoplasm	Protein metabolism and modification
P46821	Microtubule-associated protein 1B	Cytoskeleton	Cell structure and mobility
P46940	Ras GTPase-activating-like protein IQGAP1	Cytoskeleton	Signal transduction
			Cell cycle
P46976	Glycogenin 1	Cytoplasm	Carbohydrate metabolism
P47756	F-actin cappling protein subunit beta	Cytoskeleton	Cell structure and mobility
P47914	60S ribosomal protein L29	Cytoplasm	Protein metabolism and modification
P47929	Lectin, galactoside-binding, soluble, 7 (galectin 7)	Extracellular Region	Cell adhesion
			Immunity and defense
			Apoptosis
P48594	Squamous cell carcinoma antigen 2; Serpin B4	Cytoplasm	Protein metabolism and modification
P49189	aldehyde dehydrogenase 9A1	Cytoplasm	Nucleoside, nucleotide and nucleic acid metabolism
P49327	Fatty acid synthase	Cytoplasm	Amino acid metabolism
			Lipid, fatty acid and steroid metabolism
P49411	Tu translation elongation factor, mitochondrial	Mitochondrion	Protein metabolism and modification
P49773	Histidine triad nucleotide-binding protein 1	Cytoskeleton	Signal transduction
P49862	Kallikrein-7 precursor	Extracellular Region	Protein metabolism and modification
			Developmental processes
			Cell cycle
			Cell proliferation and differentiation

Accession No	Protein Name	Cellular Localisation	Functional Terms
P49913	Cathelicidin antimicrobial peptide precursor	Extracellular Region	Immunity and defense
P50453	Serpin B9	Cytoplasm	Protein metabolism and modification
P50750	Cell division protein kinase 9	Nucleus	Protein metabolism and modification
			Cell cycle
P50914	60S ribosomal protein L14	Cytoplasm	Protein metabolism and modification
P50995	Annexin A11	Envelope	Lipid, fatty acid and steroid metabolism
P51149	Ras-related protein Rab-7	Lysosome	Signal transduction
			Intracellular protein traffic
P51589	Cytochrome P450 2J2	Endoplasmic Reticulum	Lipid, fatty acid and steroid metabolism
			Electron transport
P51659	Peroxisomal multifunctional enzyme type 2	Cytoplasm	Lipid, fatty acid and steroid metabolism
P51805	Plexin-A3 precursor	Membrane	Protein metabolism and modification
			Signal transduction
			Developmental processes
P51884	Lumican precursor	Extracellular Region	Signal transduction
			Sensory perception
P51991	Heterogeneous nuclear ribonucleoprotein A3	Nucleus	Nucleoside, nucleotide and nucleic acid metabolism
P52209	6-phosphogluconate dehydrogenase, decarboxylating	ND	Carbohydrate metabolism
P52565	Rho GDP dissociation inhibitor (GDI) alpha	Cytoskeleton	Signal transduction
P52566	Rho GDP dissociation inhibitor (GDI) beta	Cytoskeleton	Signal transduction
P52907	F-actin capping protein alpha-1 subunit	Cytoskeleton	Cell structure and mobility
P54108	Cysteine-rich secretory protein 3	Extracellular Region	Developmental processes
P54253	Ataxin-1	Nucleus	Nucleoside, nucleotide and nucleic acid metabolism
P54652	Heat shock-related 70 kDa protein 2	Cell Surface	Protein metabolism and modification
			Immunity and defense
P55000	Secreted Ly-6/uPAR-related protein 1 precursor	Extracellular Region	Immunity and defense
P55072	Transitional endoplasmic reticulum ATPase	Nucleus	Protein metabolism and modification
			Intracellular protein traffic
			Protein targeting and localization
			Transport

Accession No	Protein Name	Cellular Localisation	Functional Terms
P55145	ARMET protein precursor	Extracellular Region	Neuronal activities
P55196	Afadin (Protein AF-6)	Membrane	Signal transduction
			Cell adhesion
			Cell structure and mobility
P55786	Puromycin-sensitive aminopeptidase	Cytoplasm	Protein metabolism and modification
P56537	Eukaryotic translation initiation factor 6	Nucleus	Protein metabolism and modification
P58062	Serine protease inhibitor Kazal-type 7 precursor	Extracellular Region	Protein metabolism and modification
P58546	Myotrophin	Intracellular	Protein metabolism and modification
			Developmental processes
			Cell proliferation and differentiation
P59665	Neutrophil defensin 1 precursor	Extracellular Region	Immunity and defense
P59666	Neutrophil defensin 3 precursor	Extracellular Region	Immunity and defense
P60174	Triosephosphate isomerase	Cytoplasm	Carbohydrate metabolism
			Nucleoside, nucleotide and nucleic acid metabolism
P60510	Serine/threonine-protein phosphatase 4 catalytic subunit	Cytoskeleton	Cell structure and mobility
P60709	Actin, cytoplasmic 1	Cytoskeleton	Intracellular protein traffic
			Transport
			Cell cycle
			Cell structure and mobility
P60866	40S ribosomal protein S20	Cytoplasm	Protein metabolism and modification
P60900	Proteasome subunit alpha type 6	Cytoplasm	Protein metabolism and modification
P60903	S100 calcium binding protein A10; Calpactin I light chain	Mitochondrion	Developmental processes
P60953	Cell division control protein 42 homolog precursor	Membrane	Signal transduction
			Cell structure and mobility
P60985	Keratinocyte differentiation-associated protein	Extracellular Region	Developmental processes
			Cell proliferation and differentiation
P61160	Actin-like protein 2	Cytoskeleton	Intracellular protein traffic
			Transport
			Cell cycle
			Cell structure and mobility

Accession No	Protein Name	Cellular Localisation	Functional Terms
P61254	60S ribosomal protein L26	Cytoplasm	Protein metabolism and modification
P61457	Pterin-4-alpha-carbinolamine dehydratase	Nucleus	Nucleoside, nucleotide and nucleic acid metabolism
			Coenzyme and prosthetic group metabolism
P61586	Transforming protein RhoA	Cytoskeleton	Signal transduction
			Cell structure and mobility
P61626	Lysozym C	Extracellular Region	Carbohydrate metabolism
			Immunity and defense
P61769	Beta-2-microglobulin	Cytoplasm	Immunity and defense
P61916	Epididymal secretory protein E1	Lysosome	Lipid, fatty acid and steroid metabolism
			Transport
P61978	Heterogeneous nuclear ribonucleoprotein K	Nucleus	Nucleoside, nucleotide and nucleic acid metabolism
			Protein metabolism and modification
			Signal transduction
			Intracellular protein traffic
			Apoptosis
P62081	40S ribosomal protein S7	Nucleus	Protein metabolism and modification
P62158	Calmodulin	Membrane	Signal transduction
			Cell cycle
			Cell proliferation and differentiation
P62195	Protease regulatory subunit 8, 26S	Nucleus	Protein metabolism and modification
P62244	40S ribosomal protein S15a	Cytoplasm	Protein metabolism and modification
P62249	40S ribosomal protein S16	Cytoplasm	Protein metabolism and modification
P62263	40S ribosomal protein S14	Cytoplasm	Protein metabolism and modification
P62266	40S ribosomal protein S23	Cytoplasm	Protein metabolism and modification
P62269	40S ribosomal protein S18	Cytoplasm	Protein metabolism and modification
P62280	40S ribosomal protein S11	Cytoplasm	Protein metabolism and modification
P62318	Small nuclear ribonucleoprotein Sm D3	Nucleus	Nucleoside, nucleotide and nucleic acid metabolism
P62328	Thymosin beta-4	Cytoskeleton	Cell structure and mobility
P62330	ADP-ribosylation factor 6	Membrane	Intracellular protein traffic
P62424	60S ribosomal protein L7a	Membrane Fraction	Protein metabolism and modification

Accession No	Protein Name	Cellular Localisation	Functional Terms
P62736	Actin, aortic smooth muscle	Cytoskeleton	Intracellular protein traffic
			Transport
			Cell cycle
			Cell structure and mobility
P62753	40S ribosomal protein S6	Nucleus	Protein metabolism and modification
P62805	Histone H4	Nucleus	Nucleoside, nucleotide and nucleic acid metabolism
P62807	Histone H2B.a/g/h/k/l	Intracellular	Other metabolism
P62829	60S ribosomal protein L23	Cytoplasm	Protein metabolism and modification
P62847	40S ribosomal protein S24	Cytoplasm	Protein metabolism and modification
P62851	40S ribosomal protein S25	Cytoplasm	Protein metabolism and modification
P62854	40S ribosomal protein S26	Cytoplasm	Protein metabolism and modification
P62861	40S ribosomal protein S30	Cytoplasm	Protein metabolism and modification
P62888	60S ribosomal protein L30	Cytoplasm	Protein metabolism and modification
P62899	60S ribosomal protein L31	Cytoplasm	Protein metabolism and modification
P62906	60S ribosomal protein L10a	Cytoplasm	Protein metabolism and modification
P62910	60S ribosomal protein L32	Cytoplasm	Protein metabolism and modification
P62917	60S ribosomal protein L8	Cytoplasm	Protein metabolism and modification
P62937	Peptidyl-prolyl cis-trans isomerase A (Cyclophilin A)	Extracellular Region	Protein metabolism and modification
			Intracellular protein traffic
			Immunity and defense
P62988	Ubiquitin	Nucleus	Protein metabolism and modification
P63104	14-3-3 protein zeta/delta	Cytoplasm	Signal transduction
			Protein targeting and localization
			Cell cycle
P63167	Dynein light chain 1, cytoplasmic	Membrane	Intracellular protein traffic
			Developmental processes
P63173	60S ribosomal protein L38	Cytoplasm	Protein metabolism and modification
P63220	40S ribosomal protein S21	Cytoplasm	Protein metabolism and modification
P63241	Eukaryotic initiation factor 5A isoform I variant A	Envelope	Protein metabolism and modification
P63313	Thymosin beta-10	Cytoskeleton	Cell structure and mobility

Accession No	Protein Name	Cellular Localisation	Functional Terms
P67775	Serine/threonine-protein phosphatase 2A catalytic subunit alpha isoform	Cytoskeleton	Lipid, fatty acid and steroid metabolism
			Nucleoside, nucleotide and nucleic acid metabolism
			Protein metabolism and modification
			Signal transduction
			Cell adhesion
			Apoptosis
			Developmental processes
			Cell proliferation and differentiation
			Other metabolism
P67936	Tropomyosin alpha 4 chain	Cytoskeleton	Muscle contraction
			Developmental processes
			Cell structure and mobility
P68032	Actin, alpha cardiac muscle 1	Cytoskeleton	Intracellular protein traffic
			Transport
			Cell cycle
			Cell structure and mobility
P68036	Ubiquitin-conjugating enzyme E2 L3	Intracellular	Protein metabolism and modification
P68104	eukaryotic translation elongation factor 1 alpha 1	Cytoplasm	Protein metabolism and modification
P68366	Tubulin alpha-1 chain	Cytoskeleton	Intracellular protein traffic
			Cell cycle
			Cell structure and mobility
P68431	Histone H3.1	Nucleus	Nucleoside, nucleotide and nucleic acid metabolism
P68871	Hemoglobin beta chain	Cytoplasm	Transport
			Other metabolism
P69891	Hemoglobin subunit gamma-1	Cytoplasm	Transport
			Blood circulation and gas exchange
P69905	Hemoglobin alpha subunit	Cytoplasm	Transport
			Blood circulation and gas exchange
P78417	Glutathione transferase omega 1	Cytoplasm	Immunity and defense
P80188	Neutrophil gelatinase-associated lipocalin	Cytoplasm	Transport

Accession No	Protein Name	Cellular Localisation	Functional Terms
			Immunity and defense
			Oncogenesis
P80511	Protein S100-A12	Cytoplasm	Immunity and defense
P80723	Brain abundant, membrane attached signal protein 1; Brain acid soluble protein 1	Membrane	Intracellular protein traffic
			Neuronal activities
			Developmental processes
P81605	Dermcidin precursor	Extracellular Region	Immunity and defense
P83731	60S ribosomal protein L24	Cytoplasm	Protein metabolism and modification
P84103	Splicing factor, arginine/serine-rich 3	Nucleus	Nucleoside, nucleotide and nucleic acid metabolism
P98088	Mucin-5AC precursor	Extracellular Region	Developmental processes
P98187	Cytochrome P450 4F8	Endoplasmic Reticulum	Lipid, fatty acid and steroid metabolism
			Electron transport
P99999	Cytochrome c	Mitochondrion	Electron transport
			Apoptosis
Q00610	clathrin heavy chain 1	Membrane	Signal transduction
			Intracellular protein traffic
			Neuronal activities
Q00688	FK506-binding protein 3	Nucleus	Protein metabolism and modification
			Signal transduction
			Immunity and defense
			Neuronal activities
			Cell cycle
Q00796	Sorbitol dehydrogenase	ND	Carbohydrate metabolism
			Other metabolism
Q00839	heterogeneous nuclear ribonucleoprotein U isoform a	Nucleus	Nucleoside, nucleotide and nucleic acid metabolism
Q01082	Spectrin beta chain, brain 1	Cytoskeleton	Cell structure and mobility
Q01105	Protein SET	Nucleus	Nucleoside, nucleotide and nucleic acid metabolism
			Apoptosis
			Cell cycle
Q01459	Di-N-acetylchitobiase precursor	Lysosome	Carbohydrate metabolism

Accession No	Protein Name	Cellular Localisation	Functional Terms
Q01469	Fatty acid-binding protein, epidermal	Cytoplasm	Lipid, fatty acid and steroid metabolism
			Coenzyme and prosthetic group metabolism
			Signal transduction
			Transport
			Developmental processes
Q01518	CAP, adenylate cyclase-associated protein 1 (yeast)	Cytoskeleton	Signal transduction
			Cell structure and mobility
Q01954	Zinc finger protein basonuclin-1	Nucleus	Sensory perception
			Developmental processes
Q02383	Semenogelin-2	Extracellular Region	Developmental processes
Q02413	Desmoglein-1 precursor	Membrane	Signal transduction
			Cell adhesion
Q02487	Desmocollin-2 precursor	Cytoskeleton	Signal transduction
			Cell adhesion
Q02818	Nucleobindin-1 precursor	Extracellular Region	Homeostasis
Q02878	60S ribosomal protein L6	Cytoplasm	Protein metabolism and modification
Q03013	glutathione S-transferase M4 isoform 1	Cytoskeleton	Immunity and defense
Q03252	Lamin B2	Envelope	Cell structure and mobility
Q05524	Alpha-enolase, lung specific	Cytoplasm	Carbohydrate metabolism
Q05639	Elongation factor 1-alpha 2	Nucleus	Protein metabolism and modification
Q05682	Caldesmon	Cytoskeleton	Cell cycle
			Cell structure and mobility
Q06323	Proteasome activator complex subunit 1	Cytoplasm	Protein metabolism and modification
Q06830	Peroxiredoxin 1	Cytoplasm	Immunity and defense
Q07065	Cytoskeleton associated protein 4	Membrane	ND
Q07157	Tight-junction protein ZO-1	Membrane	Cell structure and mobility
Q07654	trefoil factor 3 precursor	Extracellular Region	Signal transduction
			Developmental processes
			Cell structure and mobility
Q08188	Protein-glutamine gamma-glutamyltransferase E precursor	Membrane	Developmental processes

Accession No	Protein Name	Cellular Localisation	Functional Terms
Q08380	Galectin-3-binding protein precursor	Extracellular Region	Signal transduction
			Immunity and defense
Q08EQ4	Thymosin beta-4-like protein 1	Cytoplasm	Cell structure and mobility
Q09666	Neuroblast differentiation-associated protein AHNAK	Nucleus	Developmental processes
Q0VD83	apolipoprotein B48 receptor	Membrane	Lipid, fatty acid and steroid metabolism
Q10588	ADP-ribosyl cyclase 2 precursor	Membrane	Nucleoside, nucleotide and nucleic acid metabolism
Q12802	A-kinase anchor protein 13 (AKAP 13)	Membrane	Amino acid metabolism
			Signal transduction
			Cell adhesion
			Intracellular protein traffic
			Transport
			Apoptosis
			Cell cycle
			Cell structure and mobility
Q12841	Follistatin-related protein 1 precursor	Extracellular Region	Homeostasis
Q12888	Tumor suppressor p53-binding protein 1	Nucleus	Nucleoside, nucleotide and nucleic acid metabolism
Q12906	Interleukin enhancer-binding factor 3	Nucleus	Nucleoside, nucleotide and nucleic acid metabolism
			Cell cycle
Q13011	Delta(3,5)-Delta(2,4)-dienoyl-CoA isomerase, mitochondrial precursor	Cytoplasm	Carbohydrate metabolism
			Lipid, fatty acid and steroid metabolism
			Coenzyme and prosthetic group metabolism
Q13162	Peroxiredoxin-4	Mitochondrion	Immunity and defense
Q13231	Chitotriosidase-1 precursor	Extracellular Region	Carbohydrate metabolism
Q13283	Ras-GTPase-activating protein-binding protein 1	Nucleus	Transport
Q13445	Transmembrane emp24 domain-containing protein 1 precursor	Membrane	Intracellular protein traffic
Q13557	calcium/calmodulin-dependent protein kinase II delta isoform 1	Intracellular	Protein metabolism and modification
			Signal transduction
			Muscle contraction
			Developmental processes
Q13614	Myotubularin-related protein 2	Nucleus	Lipid, fatty acid and steroid metabolism

Accession No	Protein Name	Cellular Localisation	Functional Terms
			Developmental processes
Q13765	Nascent polypeptide-associated complex alpha subunit	Cytoplasm	Nucleoside, nucleotide and nucleic acid metabolism
Q13813	Spectrin alpha chain, brain	Cytoskeleton	Protein metabolism and modification
			Cell structure and mobility
Q13835	Plakophilin 1	Cytoskeleton	Signal transduction
			Cell adhesion
Q13867	Bleomycin hydrolase	Nucleus	Protein metabolism and modification
Q14055	Collagen alpha-2(IX) chain precursor	Extracellular Region	Signal transduction
			Cell adhesion
			Developmental processes
Q14116	Interleukin-18 precursor	Extracellular Region	Signal transduction
			Immunity and defense
Q14134	Tripartite motif-containing protein 29	Cytoplasm	Protein metabolism and modification
Q14166	Tubulin--tyrosine ligase-like protein 12	ND	Protein metabolism and modification
Q14210	Lymphocyte antigen 6D precursor	Membrane	Cell adhesion
Q14508	WAP four-disulfide core domain protein 2 precursor	Extracellular Region	Protein metabolism and modification
Q14515	SPARC-like protein 1 precursor	Extracellular Region	ND
Q14624	Inter-alpha-trypsin inhibitor heavy chain H4 precursor	Extracellular Region	Protein metabolism and modification
Q14651	Plastin-1	Cytoplasm	Cell structure and mobility
Q14677	Clathrin interactor-1	Cytoplasm	Signal transduction
Q14764	Major vault protein	Nucleus	Intracellular protein traffic
			Protein targeting and localization
			Immunity and defense
Q14839	Chromodomain helicase-DNA-binding protein 4	Nucleus	Nucleoside, nucleotide and nucleic acid metabolism
Q15056	Eukaryotic translation initiation factor 4H	Cytoplasm	Protein metabolism and modification
Q15075	Early endosome antigen 1	Membrane	Intracellular protein traffic
Q15084	Protein disulfide-isomerase A6 precursor	Endoplasmic Reticulum	Protein metabolism and modification
Q15149	Plectin-1	Membrane	Cell structure and mobility
Q15185	Prostaglandin E synthase 3 (Cytosolic prostaglandin E2 synthase)	Nucleus	Lipid, fatty acid and steroid metabolism
			Signal transduction

Accession No	Protein Name	Cellular Localisation	Functional Terms
Q15365	Poly(rC)-binding protein 1	Nucleus	Protein metabolism and modification
Q15424	Scaffold attachment factor B	Nucleus	Nucleoside, nucleotide and nucleic acid metabolism
Q15459	Splicing factor 3 subunit 1	Nucleus	Nucleoside, nucleotide and nucleic acid metabolism
Q15468	SCL-interrupting locus protein	Cytoplasm	Cell proliferation and differentiation
Q15555	Microtubule-associated protein RB/EB family member 2	Cytoskeleton	Cell cycle
			Cell proliferation and differentiation
			Cell structure and mobility
Q15642	Cdc-42 interacting protein 4	Cytoplasm	Cell cycle
			Cell structure and mobility
Q15643	Thyroid receptor-interacting protein 11	Golgi Apparatus	Nucleoside, nucleotide and nucleic acid metabolism
Q15651	High mobility group nucleosome-binding domain-containing protein 3	Nucleus	Nucleoside, nucleotide and nucleic acid metabolism
			Cell cycle
Q15843	NEDD8	Nucleus	Protein metabolism and modification
Q15847	Adipose most abundant gene transcript 2 protein	Cellular	ND
Q16270	Insulin-like growth factor-binding protein 7 precursor	Extracellular Region	Cell proliferation and differentiation
Q16363	Laminin-alpha-4 chain precursor	Extracellular Region	Signal transduction
			Cell adhesion
Q16537	serine/threonine protein phosphatase 2A, 56 kDa regulatory subunit, epsilon isoform	Cytoplasm	Protein metabolism and modification
			Signal transduction
Q16610	Extracellular matrix protein 1 precursor	Extracellular Region	Signal transduction
Q16629	Splicing factor, arginine/serine-rich 7	Nucleus	Nucleoside, nucleotide and nucleic acid metabolism
Q16695	Histone H3.1t	Intracellular	Nucleoside, nucleotide and nucleic acid metabolism
Q16825	Tyrosine-protein phosphatase non-receptor type 21	Cytoskeleton	Protein metabolism and modification
			Developmental processes
			Cell structure and mobility
Q1U7T2	Oligopeptide/dipeptide ABC transporter, ATP-binding protein-like	ND	ND
Q32MZ4	Leucine-rich repeat flightless-interacting protein 1	Cytoskeleton	Nucleoside, nucleotide and nucleic acid metabolism
Q3KNS1	Patched domain-containing protein 3	Membrane	Signal transduction
			Developmental processes
Q3KQU3	MAP7 domain-containing protein 1	ND	Developmental processes

Accession No	Protein Name	Cellular Localisation	Functional Terms
			Cell structure and mobility
Q3MII2	Serine proteinase inhibitor	Cytoplasm	Protein metabolism and modification
Q495M9	Usher syndrome type-1G protein	Cytoskeleton	Sensory perception
Q4L180	GPBP-interacting protein 130d	Cytoskeleton	ND
Q53FA7	Putative quinone oxidoreductase	Cellular	Carbohydrate metabolism
			Nucleoside, nucleotide and nucleic acid metabolism
			Apoptosis
Q53GZ6	Heat shock 70kDa protein 8 isoform 1	Cytoplasm	Protein metabolism and modification
			Immunity and defense
Q53RT3	Retroviral-like aspartic protease 1 precursor	Membrane	Protein metabolism and modification
			Developmental processes
Q5CZC0	Fibrous sheath-interacting protein 2	ND	ND
Q5D862	Filaggrin-2 (FLG-2)	ND	Miscellaneous
Q5H9J7	Protein BEX5	Cytoplasm	ND
Q5QNY2	Heat shock 70kDa protein	ND	Protein metabolism and modification
			Immunity and defense
Q5T0N1	Tetratricopeptide repeat-containing protein	ND	Protein metabolism and modification
			Signal transduction
Q5T0Z8	Uncharacterized protein C6orf132	ND	ND
Q5T3I0	G patch domain containing 4 protein isoform 1	Intracellular	ND
Q5TZ20	Olfactory receptor 2G6	Membrane	Signal transduction
			Neuronal activities
			Sensory perception
Q5TZA2	Rootletin	Cytoskeleton	Cell cycle
Q5VTE0	Putative elongation factor 1-alpha-like 3	Cytoplasm	Protein metabolism and modification
Q5VTM1	Protein FAM25	ND	ND
Q6E0U4	Dermokine precursor	Extracellular Region	Cell proliferation and differentiation
Q6IBS0	Twinfilin-2	Cytoskeleton	Cell structure and mobility
Q6N089	Ig gamma-1 chain C region	Membrane	Immunity and defense
Q6P2D8	X-ray radiation resistance-associated protein 1	Nucleus	Immunity and defense

Accession No	Protein Name	Cellular Localisation	Functional Terms
Q6P3W6	Neuroblastoma breakpoint family member 10	Cytoplasm	ND
Q6P4A8	Putative phospholipase B-like 1 precursor	Extracellular Region	Lipid, fatty acid and steroid metabolism
Q6UWN5	Ly6/PLAUR domain-containing protein 5 precursor	Membrane	ND
Q6UWP8	Suprabasin precursor	Extracellular Region	ND
Q6WCQ1	Myosin phosphatase-Rho interacting protein	Cytoskeleton	Signal transduction
			Cell structure and mobility
Q6XPR3	Repetin	Extracellular Region	Developmental processes
			Cell proliferation and differentiation
Q6ZMR5	Transmembrane protease, serine 11A	Membrane	Protein metabolism and modification
Q6ZN66	Guanylate-binding protein 6	ND	Immunity and defense
Q6ZVX7	Putative uncharacterized protein LOC342897	ND	Protein metabolism and modification
Q71DI3	Histone H3.2	Nucleus	Nucleoside, nucleotide and nucleic acid metabolism
Q71UI9	Histone H2AV	Nucleus	Nucleoside, nucleotide and nucleic acid metabolism
Q71UM5	40S ribosomal protein S27-like protein	Cellular	Protein metabolism and modification
Q7L7L0	Histone H2A type 3	Nucleus	Nucleoside, nucleotide and nucleic acid metabolism
Q7Z3Z4	Piwi-like protein 4	ND	Nucleoside, nucleotide and nucleic acid metabolism
			Developmental processes
Q7Z406	myosin, heavy chain 14 isoform 1	Cytoskeleton	Developmental processes
			Cell structure and mobility
Q86SG5	Protein S100-A7-like 1	Cytoplasm	Immunity and defense
			Cell proliferation and differentiation
Q86T26	Transmembrane protease, serine 11B	Membrane	Protein metabolism and modification
Q86UP2	Kinectin	Membrane	Intracellular protein traffic
			Cell structure and mobility
Q86VD1	MORC family CW-type zinc finger 1	Nucleus	Developmental processes
Q86XP0	Cytosolic phospholipase A2 delta	Membrane	Lipid, fatty acid and steroid metabolism
			Immunity and defense
			Cell proliferation and differentiation
Q86YZ3	Hornerin	Cytoplasm	Miscellaneous
Q8IUE6	Histone H2A type 2-B	Nucleus	Nucleoside, nucleotide and nucleic acid metabolism

Accession No	Protein Name	Cellular Localisation	Functional Terms
Q8IUS5	Abhydrolase domain-containing protein 7	Membrane	Nucleoside, nucleotide and nucleic acid metabolism
			Immunity and defense
Q8IVV2	Lipoxygenase homology domain-containing protein 1	ND	ND
Q8IY18	SMC5 structural maintenance of chromosomes 5-like 1	Nucleus	Nucleoside, nucleotide and nucleic acid metabolism
Q8IY33	MICAL-like protein 2	Cytoskeleton	Cell structure and mobility
Q8IZQ1	WD repeat and FYVE domain-containing protein 3	Cytoplasm	Nucleoside, nucleotide and nucleic acid metabolism
			Signal transduction
			Protein targeting and localization
Q8N0V4	Leucine-rich repeat LGI family member 2 precursor	Extracellular Region	ND
Q8N1A0	Keratin-like protein KRT222	Cytoskeleton	Cell structure and mobility
Q8N257	Histone H2B type 3-B	Nucleus	Nucleoside, nucleotide and nucleic acid metabolism
Q8N2Z9	Centromere protein S	Nucleus	Signal transduction
Q8N355	IGLC1 protein	Membrane	Immunity and defense
Q8N568	Serine/threonine-protein kinase DCAMKL2	ND	Protein metabolism and modification
			Neuronal activities
			Cell structure and mobility
Q8N6Q3	CD177 antigen precursor	Membrane	ND
Q8N7U6	EF-hand domain-containing family member B	ND	ND
Q8NA31	Coiled-coil domain-containing protein 79	ND	Nucleoside, nucleotide and nucleic acid metabolism
Q8NAC3	Interleukin-17 receptor C precursor	Membrane	ND
Q8NBI6	Protein C3orf21	Membrane	ND
Q8NC51	Plasminogen activator inhibitor 1 RNA-binding protein	Nucleus	Nucleoside, nucleotide and nucleic acid metabolism
Q8NCB2	CaM kinase-like vesicle-associated protein	Membrane	Protein metabolism and modification
			Signal transduction
Q8NCR0	UDP-GalNAc:beta-1,3-N-acetylgalactosaminyltransferase 2	Golgi Apparatus	Protein metabolism and modification
Q8NFC6	Protein FAM44A	ND	ND
Q8NGC9	Olfactory receptor 11H4	Membrane	Signal transduction
			Sensory perception
Q8NHM4	Putative trypsin-6	Extracellular Region	Protein metabolism and modification
			Cell structure and mobility

Accession No	Protein Name	Cellular Localisation	Functional Terms
Q8NHS3	Major facilitator superfamily domain-containing protein 8	Lysosome	Immunity and defense
Q8TC20	Cancer-associated gene 1 protein	ND	ND
Q8TD31	Coiled-coil alpha-helical rod protein 1	Nucleus	Intracellular protein traffic
			Protein targeting and localization
			Developmental processes
Q8TDC3	BR serine/threonine-protein kinase 1	Nucleus	Protein metabolism and modification
Q8TDL5	Long palate, lung and nasal epithelium carcinoma associated protein 1	Extracellular Region	Immunity and defense
Q8TE68	Epidermal growth factor receptor kinase substrate 8-like protein 1	Cellular	Signal transduction
			Cell structure and mobility
Q8TER0	Sushi, nidogen and EGF-like domain-containing protein 1	ND	Signal transduction
			Immunity and defense
			Developmental processes
			Cell structure and mobility
Q8TER5	Protein SOLO	Intracellular	Signal transduction
Q8WV44	Tripartite motif protein 41	Nucleus	Protein metabolism and modification
Q8WVV4	Premature ovarian failure, 1B	ND	Developmental processes
Q8WW22	DnaJ homolog subfamily A member 4	Membrane	Protein metabolism and modification
			Immunity and defense
Q8WWI1	LIM domain only protein 7	Nucleus	Muscle contraction
Q8WWY7	WAP four-disulfide core domain protein 12 precursor	Extracellular Region	Protein metabolism and modification
Q8WXH0	Nesprin-2	Nucleus	Cell structure and mobility
Q8WXI7	Mucin-16	Membrane	Cell adhesion
Q8WXX0	Ciliary dynein heavy chain 7	Cytoskeleton	Developmental processes
Q8WYL5	Protein phosphatase Slingshot homolog 1	Membrane	Protein metabolism and modification
			Signal transduction
			Developmental processes
			Cell structure and mobility
Q8WYP5	transcription factor ELYS	Nucleus	Nucleoside, nucleotide and nucleic acid metabolism
			Developmental processes
Q8WZ42	Titin	Nucleus	Muscle contraction

Accession No	Protein Name	Cellular Localisation	Functional Terms
Q92597	Protein NDRG1	Nucleus	Cell proliferation and differentiation
Q92614	Myosin-XVIIIa (Myosin containing a PDZ domain)	Nucleus	Muscle contraction
			Developmental processes
			Cell structure and mobility
Q92736	Ryanodine recpetor 2	Membrane	Signal transduction
			Transport
			Muscle contraction
Q92765	Secreted frizzled-related protein 3 precursor	Extracellular Region	Signal transduction
Q92817	Envoplakin	Cytoskeleton	Cell adhesion
			Developmental processes
			Cell structure and mobility
Q92820	Gamma-glutamyl hydrolase precursor	Lysosome	Coenzyme and prosthetic group metabolism
Q92876	Kallikrein-6 precursor	Extracellular Region	Protein metabolism and modification
Q93077	Histone H2A type 1-C	Intracellular	Nucleoside, nucleotide and nucleic acid metabolism
Q93100	Phosphorylase b kinase regulatory subunit beta	Membrane	Carbohydrate metabolism
Q96BT7	Alkylated DNA repair protein alkB homolog 8	ND	ND
Q96C19	EF-hand domain-containing protein 2	ND	Developmental processes
Q96C86	Scavenger mRNA decapping enzyme DcpS	Nucleus	Nucleoside, nucleotide and nucleic acid metabolism
Q96CS3	UBX domain-containing protein 8	Cytoplasm	ND
Q96F07	cytoplasmic FMR1 interacting protein 2	Membrane	Signal transduction
			Developmental processes
Q96FF9	Sororin	Nucleus	Cell cycle
Q96FQ6	Protein S100-A16	ND	ND
Q96FX8	p53 apoptosis effector related to PMP-22	Cytoskeleton	Apoptosis
Q96HC4	PDZ and LIM domain protein 5	Cytoskeleton	Developmental processes
			Cell structure and mobility
Q96HE7	ERO1-like protein alpha precursor	Membrane	Protein metabolism and modification
			Electron transport
			Intracellular protein traffic
			Transport

Accession No	Protein Name	Cellular Localisation	Functional Terms
			Miscellaneous
Q96JD0	Amyloid lambda 6 light chain variable region SAR	ND	ND
Q96JY6	PDZ and LIM domain protein 2	Cell Surface	Cell structure and mobility
Q96KC8	DnaJ homolog subfamily C member 1	Cytoplasm	Protein metabolism and modification
Q96P63	Serpin B12	Cytoplasm	Protein metabolism and modification
Q96PQ0	VPS10 domain-containing receptor SorCS2 precursor	Membrane	Intracellular protein traffic
Q96QH2	PML-RARA-regulated adapter molecule 1 (PRAM-1) (PRAM)	ND	Signal transduction
Q96RY5	Protein cramped-like	Nucleus	Nucleoside, nucleotide and nucleic acid metabolism
Q96S94	Cyclin-L2	Nucleus	Nucleoside, nucleotide and nucleic acid metabolism
			Cell cycle
			Cell proliferation and differentiation
Q96TA1	Niban-like protein	ND	Oncogenesis
Q99102	mucin 4 isoform a	Extracellular Region	Signal transduction
			Cell adhesion
Q99497	DJ-1 protein	Nucleus	Nucleoside, nucleotide and nucleic acid metabolism
			Immunity and defense
			Cell proliferation and differentiation
Q99523	Sortilin precursor	Membrane	Intracellular protein traffic
Q99538	Legumain precursor	Lysosome	Protein metabolism and modification
Q99835	Smoothened homolog precursor	Membrane	Signal transduction
Q99877	Histone H2B type 1-N	Intracellular	Other metabolism
Q99880	Histone H2B.c	Intracellular Nucleus	Other metabolism
Q9BPY8	Homeodomain-only protein		Developmental processes
			Cell proliferation and differentiation
Q9BQE3	Tubulin alpha-1C chain	Cytoskeleton	Intracellular protein traffic
			Cell cycle
			Cell structure and mobility
Q9BQR3	Serine protease 27 precursor	Extracellular Region	Protein metabolism and modification
Q9BRA2	Thioredoxin-like protein 5	Cytoplasm	Nucleoside, nucleotide and nucleic acid metabolism
			Electron transport

Accession No	Protein Name	Cellular Localisation	Functional Terms
Q9BS26	Thioredoxin domain-containing protein 4 precursor	Membrane	Protein metabolism and modification
Q9BW04	specifically androgen-regulated protein	Cytoplasm	ND
Q9BXL7	Caspase recruitment domain-containing protein 11	Membrane	Signal transduction
			Apoptosis
Q9BYB0	proline-rich synapse-associated protein 2 isoform 1	Membrane	Signal transduction
			Cell structure and mobility
Q9BYE4	Small proline-rich protein 2G	Cytoskeleton	Developmental processes
			Cell proliferation and differentiation
Q9BYT8	Neurolysin, mitochondrial	Mitochondrion	Protein metabolism and modification
			Intracellular protein traffic
Q9BZA7	Protocadherin-11 X-linked precursor	Membrane	Signal transduction
			Cell adhesion
			Developmental processes
			Cell proliferation and differentiation
Q9C0A6	SET domain-containing protein 5	Nucleus	Oncogenesis
Q9C0I9	Leucine-rich repeat-containing protein 27	ND	Immunity and defense
			Miscellaneous
Q9GZP4	UPF0424 protein C1orf128	ND	ND
Q9GZV4	Eukaryotic translation initiation factor 5A-2	Cytoplasm	Protein metabolism and modification
Q9H008	Phospholysine phosphohistidine inorganic pyrophosphate phosphatase	ND	Other metabolism
Q9H0W9	Ester hydrolase C11orf54	Nucleus	Other metabolism
Q9H1E1	Ribonuclease 7 precursor	Extracellular Region	Nucleoside, nucleotide and nucleic acid metabolism
Q9H201	Epsin-3	Nucleus	Signal transduction
			Intracellular protein traffic
Q9H251	Cadherin related 23	Intracellular	Signal transduction
			Cell adhesion
			Sensory perception
			Developmental processes
Q9H361	Polyadenylate-binding protein 3	Cytoplasm	Nucleoside, nucleotide and nucleic acid metabolism
Q9H3S7	Tyrosine-protein phosphatase non-receptor type 23	Cytoplasm	Protein metabolism and modification

Accession No	Protein Name	Cellular Localisation	Functional Terms
			Signal transduction
Q9H4M9	EH-domain-containing protein 1	Cellular	Intracellular protein traffic
			Neuronal activities
Q9H5V8	CUB domain-containing protein 1	Membrane	Cell adhesion
			Cell proliferation and differentiation
			Cell structure and mobility
Q9H6S3	Epidermal growth factor receptor kinase substrate 8-like protein 2	Cytoplasm	Signal transduction
			Cell structure and mobility
Q9H7D7	WD repeat-containing protein 26	Cytoplasm	Signal transduction
Q9HAY6	Beta,beta-carotene 15,15'-monooxygenase	ND	Coenzyme and prosthetic group metabolism
			Sensory perception
Q9HC84	Mucin-5B precursor	Extracellular Region	Developmental processes
Q9HCE3	Zinc finger protein 532	Nucleus	Nucleoside, nucleotide and nucleic acid metabolism
Q9HD89	Resistin precursor	Extracellular Region	Other metabolism
Q9NQ38	Serine protease inhibitor Kazal-type 5	Extracellular Region	Homeostasis
Q9NR45	Sialic acid synthase	Cytoplasm	Carbohydrate metabolism
			Protein metabolism and modification
Q9NRL2	Bromodomain adjacent to zinc finger domain protein 1A	Nucleus	Nucleoside, nucleotide and nucleic acid metabolism
Q9NS15	latent transforming growth factor beta binding protein 3	Extracellular Region	Signal transduction
			Sensory perception
			Developmental processes
Q9NSY1	BMP-2-inducible protein kinase	Nucleus	Protein metabolism and modification
Q9NU02	Ankyrin repeat domain-containing protein 5	ND	ND
Q9NX62	Inositol monophosphatase 3	Membrane	Lipid, fatty acid and steroid metabolism
Q9NYK1	Toll-like receptor 7 precursor	Membrane	Signal transduction
			Developmental processes
Q9NYQ8	Protocadherin Fat 2 precursor	Cellular	Signal transduction
			Cell adhesion
			Developmental processes
Q9NZH8	Interleukin-1 family member 9	Extracellular Region	Signal transduction

Accession No	Protein Name	Cellular Localisation	Functional Terms
			Immunity and defense
Q9NZQ8	Transient receptor potential cation channel, subfamily M, member 5	Membrane	Transport
Q9NZT1	Calmodulin-like protein 5	ND	Signal transduction
			Cell cycle
			Cell proliferation and differentiation
Q9P0G3	Kallikrein-14	Extracellular Region	Protein metabolism and modification
Q9P1Z0	Zinc finger and BTB domain-containing protein 4	Nucleus	Nucleoside, nucleotide and nucleic acid metabolism
Q9P202	Whirlin	Cytoplasm	Sensory perception
Q9P258	Protein RCC2	Nucleus	Cell cycle
Q9UBC9	Small proline-rich protein 3	Envelope	Developmental processes
			Cell proliferation and differentiation
Q9UBD6	Rhesus-associated C glycoprotein	Membrane	Transport
Q9UBG3	Cornulin	Membrane	Immunity and defense
			Cell structure and mobility
Q9UBR2	Cathepsin Z precursor	Cytoplasm	Protein metabolism and modification
Q9UBX7	Kallikrein 11 precursor	Extracellular Region	Protein metabolism and modification
			Developmental processes
			Cell cycle
			Cell proliferation and differentiation
Q9UFN0	Protein NipSnap3A	Cytoplasm	Intracellular protein traffic
Q9UGL9	NICE-1 protein	ND	ND
Q9UGM3	deleted in malignant brain tumors 1 isoform c precursor	Extracellular Region	Transport
			Cell proliferation and differentiation
			Oncogenesis
Q9UGV6	High mobility group protein 1-like 10	Intracellular	Nucleoside, nucleotide and nucleic acid metabolism
Q9UH77	Kelch-like protein	Cytoskeleton	Neuronal activities
			Cell structure and mobility
Q9UHG3	Prenylcysteine oxidase precursor	Lysosome	Amino acid metabolism
			Protein metabolism and modification
			Electron transport

Accession No	Protein Name	Cellular Localisation	Functional Terms
Q9UHL4	Dipeptidyl-peptidase 2 precursor	Lysosome	Protein metabolism and modification
Q9UI42	Carboxypeptidase A4 precursor	Extracellular Region	Protein metabolism and modification
Q9UIV8	Serpin B13	Cellular	Protein metabolism and modification
Q9UJY1	Heat shock protein beta-8	Intracellular	Protein metabolism and modification
			Immunity and defense
Q9UKR0	Kallikrein-12 precursor	Extracellular Region	Protein metabolism and modification
Q9UKR3	Kallikrein 13 precursor	Extracellular Region	Protein metabolism and modification
Q9UL16	Coiled-coil domain-containing protein 19	Soluble Fraction	ND
Q9UL52	Transmembrane protease, serine 11E	Membrane	Protein metabolism and modification
Q9ULH0	Ankyrin repeat-rich membrane spanning protein	Cytoplasm	Signal transduction
Q9ULH4	Leucine-rich repeat and fibronectin type-III domain-containing protein 2 precursor	Membrane	Signal transduction
			Developmental processes
Q9ULV0	Myosin-5B	Cytoskeleton	ND
Q9UM54	Myosin-6	Envelope	Sensory perception
			Cell structure and mobility
Q9UNZ2	NSFL1 cofactor p47	Nucleus	Cell cycle
Q9UPP5	KIAA1107 protein	ND	ND
Q9UPQ7	PDZ domain-containing RING finger protein 3	ND	Signal transduction
Q9UPT5	Exocyst complex component 7	Cytoskeleton	Intracellular protein traffic
Q9UPY3	dicer1	Intracellular	Nucleoside, nucleotide and nucleic acid metabolism
Q9Y285	Phenylalanyl-tRNA synthetase alpha chain	Cytoplasm	Protein metabolism and modification
Q9Y2B0	MIR-interacting saposin-like protein precursor	Membrane	Cell structure and mobility
Q9Y2V2	Calcium-regulated heat stable protein 1	Cytoplasm	Nucleoside, nucleotide and nucleic acid metabolism
			Signal transduction
Q9Y3T6	R3H and coiled-coil domain-containing protein 1	ND	ND
Q9Y446	Plakophilin-3	Cytoskeleton	Signal transduction
			Cell adhesion
Q9Y490	Talin-1	Membrane	Cell structure and mobility
Q9Y4K1	Absent in melanoma 1 protein	Cellular	Oncogenesis
Q9Y5Y6	Suppressor of tumorigenicity protein 14	Membrane	Protein metabolism and modification

Accession No	Protein Name	Cellular Localisation	Functional Terms
			Cell structure and mobility
Q9Y6R7	Fc fragment of IgG binding protein	Membrane	Cell adhesion
			Developmental processes
Q9Y6V0	Protein piccolo (Aczonin)	Cytoskeleton	Neuronal activities
			Cell structure and mobility
A1A5D9	Coiled-coil domain-containing protein 64B	ND	ND
A1L4H1	Scavenger receptor cysteine-rich domain-containing protein LOC284297	Extracellular Region	ND
A6NIZ1	Ras-related protein Rap-1b-like protein	Membrane	Signal transduction
A6NL71	Transmembrane protease, serine 11E2	Membrane	Protein metabolism and modification
A6NMB1	Sialic acid-binding Ig-like lectin 16	Membrane	Cell adhesion
A8MW06	Thymosin beta-4-like protein 3	Cytoskeleton	ND
O00231	26S proteasome non-ATPase regulatory subunit 11	Intracellular	Protein metabolism and modification
O00515	Ladinin-1	Extracellular Region	ND
O14639	Actin-binding LIM protein 1	Cytoskeleton	Cell structure and mobility
O14818	Proteasome subunit alpha type-7	Nucleus	Protein metabolism and modification
O14908	PDZ domain-containing protein GIPC1	Soluble Fraction	Oncogenesis
			Signal transduction
			Developmental processes
O14949	Cytochrome b-c1 complex subunit 8	Mitochondrion	Electron transport
			Transport
O14950	Myosin regulatory light chain MRLC2	Cytoskeleton	Muscle contraction
			Cell structure and mobility
O14979	Heterogeneous nuclear ribonucleoprotein D-like	Nucleus	Nucleoside, nucleotide and nucleic acid metabolism
O15143	Actin-related protein 2/3 complex subunit 1B	Cytoskeleton	Cell structure and mobility
O43150	Arf-GAP with SH3 domain, ANK repeat and PH domain-containing protein 2	Golgi Apparatus	Transport
O43189	PHD finger protein 1	Nucleus	Developmental processes
O43768	Alpha-endosulfine	Intracellular	Signal transduction
O60701	UDP-glucose 6-dehydrogenase	ND	Carbohydrate metabolism
			Electron transport
O60869	Endothelial differentiation-related factor 1	Nucleus	Nucleoside, nucleotide and nucleic acid metabolism

Accession No	Protein Name	Cellular Localisation	Functional Terms
O75347	Tubulin-specific chaperone A	Cytoplasm	Protein metabolism and modification
O75368	SH3 domain-binding glutamic acid-rich-like protein	ND	ND
O75874	Isocitrate dehydrogenase [NADP] cytoplasmic	Intracellular	Carbohydrate metabolism
O75969	A-kinase anchor protein 3	Cytoplasm	Signal transduction
			Protein targeting and localization
O94833	Bullous pemphigoid antigen 1, isoforms 6/9/10	Cytoplasm	Cell structure and mobility
O95197	Reticulon-3	Endoplasmic Reticulum	Intracellular protein traffic
O95782	AP-2 complex subunit alpha-1	Golgi Apparatus	Intracellular protein traffic
O95817	BAG family molecular chaperone regulator 3	Intracellular	Apoptosis
O95833	Chloride intracellular channel protein 3	Nucleus	Transport
			Homeostasis
P00167	Cytochrome b5	Mitochondrion	Lipid, fatty acid and steroid metabolism
			Electron transport
P00966	Argininosuccinate synthase	Cytoplasm	Amino acid metabolism
P01036	Cystatin-S	Extracellular Region	Protein metabolism and modification
P01598	Ig kappa chain V-I region EU	Extracellular Region	Immunity and defense
P01599	Ig kappa chain V-I region Gal	Extracellular Region	Immunity and defense
P01614	Ig kappa chain V-II region Cum	Extracellular Region	Immunity and defense
P01619	Ig kappa chain V-III region B6	Extracellular Region	Immunity and defense
P01623	Ig kappa chain V-III region WOL	Extracellular Region	Immunity and defense
P01700	Ig lambda chain V-I region HA	Extracellular Region	Immunity and defense
P01717	Ig lambda chain V-IV region Hil	Extracellular Region	Immunity and defense
P01765	Ig heavy chain V-III region TIL	Extracellular Region	Immunity and defense
P01767	Ig heavy chain V-III region BUT	Extracellular Region	Immunity and defense
P02144	Myoglobin	Cytoplasm	Muscle contraction
P02452	Collagen alpha-1(I) chain	Membrane Fraction	Cell structure and mobility
P02654	Apolipoprotein C-I	Extracellular Region	Lipid, fatty acid and steroid metabolism
P02656	Apolipoprotein C-III	Extracellular Region	Transport
			Lipid, fatty acid and steroid metabolism
P02735	Serum amyloid A protein	Extracellular Region	Immunity and defense

Accession No	Protein Name	Cellular Localisation	Functional Terms
P04179	Superoxide dismutase [Mn], mitochondrial	Mitochondrion	Electron transport
P04632	Calpain small subunit 1	Nucleus	Protein metabolism and modification
			Signal transduction
P04839	Cytochrome b-245 heavy chain	Membrane	Electron transport
			Transport
			Immunity and defense
P06576	ATP synthase subunit beta, mitochondrial [Precursor]	Mitochondrion	Nucleoside, nucleotide and nucleic acid metabolism
P06703	Protein S100-A6	Envelope	Cell proliferation and differentiation
			Signal transduction
			Cell cycle
P06727	Apolipoprotein A-IV	Extracellular Region	Lipid, fatty acid and steroid metabolism
P06737	Glycogen phosphorylase, liver form	Soluble Fraction	Carbohydrate metabolism
P06899	Histone H2B type 1-J	Nucleus	Miscellaneous
P07099	Epoxide hydrolase 1	Membrane Fraction	Lipid, fatty acid and steroid metabolism
			Immunity and defense
P07288	Prostate-specific antigen	Extracellular Region	Protein metabolism and modification
P07602	Proactivator polypeptide	Lysosome	Lipid, fatty acid and steroid metabolism
P07738	Bisphosphoglycerate mutase	ND	Carbohydrate metabolism
P07741	Adenine phosphoribosyltransferase	Cytoplasm	Nucleoside, nucleotide and nucleic acid metabolism
P07910	Heterogeneous nuclear ribonucleoproteins C1/C2	Nucleus	Nucleoside, nucleotide and nucleic acid metabolism
P07949	Proto-oncogene tyrosine-protein kinase receptor ret	Golgi Apparatus	Protein metabolism and modification
			Oncogenesis
			Cell proliferation and differentiation
			Cell cycle
			Signal transduction
			Developmental processes
P08133	Annexin A6	Cytoplasm	Other metabolism
P08637	Low affinity immunoglobulin gamma Fc region receptor III-A	Membrane	Signal transduction
			Immunity and defense
P08865	40S ribosomal protein SA	Nucleus	Protein metabolism and modification

Accession No	Protein Name	Cellular Localisation	Functional Terms
P09467	Fructose-1,6-bisphosphatase 1	Cytoplasm	Carbohydrate metabolism
P09669	Cytochrome c oxidase polypeptide VIc	Cytoplasm	ND
P0C0L4	Complement C4-A	Extracellular Region	Immunity and defense
P0C7M2	Putative heterogeneous nuclear ribonucleoprotein A1-like protein 3	Nucleus	Nucleoside, nucleotide and nucleic acid metabolism
P10768	S-formylglutathione hydrolase	Cytoplasm	ND
P10809	60 kDa heat shock protein, mitochondrial	Cell Surface	Protein metabolism and modification
P11215	Integrin alpha-M	Membrane	Cell adhesion
P12838	Neutrophil defensin 4	Extracellular Region	Immunity and defense
P13073	Cytochrome c oxidase subunit 4 isoform 1, mitochondrial	Nucleus	Electron transport
P13473	Lysosome-associated membrane glycoprotein 2	Lysosome	Protein metabolism and modification
			Intracellular protein traffic
P15428	15-hydroxyprostaglandin dehydrogenase [NAD+]	Cytoplasm	Lipid, fatty acid and steroid metabolism
P15531	Nucleoside diphosphate kinase A	Nucleus	Nucleoside, nucleotide and nucleic acid metabolism
P16152	Carbonyl reductase [NADPH] 1	Cytoplasm	Other metabolism
P16949	Stathmin	Intracellular	Signal transduction
P17096	High mobility group protein HMG-I/HMG-Y	Nucleus	Nucleoside, nucleotide and nucleic acid metabolism
P17174	Aspartate aminotransferase, cytoplasmic	Cytoplasm	Amino acid metabolism
P17655	Calpain-2 catalytic subunit	Membrane	Protein metabolism and modification
			Signal transduction
P17987	T-complex protein 1 subunit alpha	Cytoplasm	Protein metabolism and modification
P18077	60S ribosomal protein L35a	Cytoplasm	Protein metabolism and modification
P18124	60S ribosomal protein L7	Cytoplasm	Protein metabolism and modification
P19823	Inter-alpha-trypsin inhibitor heavy chain H2	Extracellular Region	ND
P19971	Thymidine phosphorylase precursor	Cytoplasm	Nucleoside, nucleotide and nucleic acid metabolism
P20671	Histone H2A type 1-D	Nucleus	Nucleoside, nucleotide and nucleic acid metabolism
P20674	Cytochrome c oxidase subunit 5A, mitochondrial	Mitochondrion	Signal transduction
P20933	N(4)-(beta-N-acetylglucosaminyl)-L-asparaginase	Lysosome	Amino acid metabolism
P20962	Parathymosin	Nucleus	Nucleoside, nucleotide and nucleic acid metabolism
			Cell proliferation and differentiation
P22234	Multifunctional protein ADE2	ND	Nucleoside, nucleotide and nucleic acid metabolism

Accession No	Protein Name	Cellular Localisation	Functional Terms
P22307	Non-specific lipid-transfer protein	Cytoplasm	Other metabolism
P22392	Nucleoside diphosphate kinase B	Nucleus	Nucleoside, nucleotide and nucleic acid metabolism
P23381	Tryptophanyl-tRNA synthetase, cytoplasmic	Soluble Fraction	Protein metabolism and modification
P24539	ATP synthase subunit b, mitochondrial	Mitochondrion	Electron transport
P25398	40S ribosomal protein S12	Cytoplasm	Protein metabolism and modification
P25705	ATP synthase subunit alpha, mitochondrial [Precursor]	Mitochondrion	ND
P26583	High mobility group protein B2	Nucleus	Signal transduction
			Nucleoside, nucleotide and nucleic acid metabolism
P26599	Polypyrimidine tract-binding protein 1	Nucleus	Nucleoside, nucleotide and nucleic acid metabolism
P27105	Erythrocyte band 7 integral membrane protein	Cytoskeleton	Cell structure and mobility
P27348	14-3-3 protein theta	Cytoplasm	Signal transduction
P27635	60S ribosomal protein L10	Cytoplasm	Protein metabolism and modification
P28072	Proteasome subunit beta type-6	Intracellular	Protein metabolism and modification
P28676	Grancalcin	Membrane	Immunity and defense
P29144	Tripeptidyl-peptidase 2	Cytoplasm	Protein metabolism and modification
P29692	Elongation factor 1-delta	Cytoplasm	Protein metabolism and modification
P29966	Myristoylated alanine-rich C-kinase substrate	Cytoskeleton	Signal transduction
P30040	Endoplasmic reticulum protein ERp29	Endoplasmic Reticulum	Intracellular protein traffic
P30044	Peroxiredoxin-5, mitochondrial precursor	Mitochondrion	Immunity and defense
P30085	UMP-CMP kinase	Nucleus	Nucleoside, nucleotide and nucleic acid metabolism
P30519	Heme oxygenase 2	Membrane	Coenzyme and prosthetic group metabolism
P34931	Heat shock 70 kDa protein 1L	Cytoplasm	Protein metabolism and modification
P35998	26S protease regulatory subunit 7	Cytoplasm	Protein metabolism and modification
P36873	Serine/threonine-protein phosphatase PP1-gamma catalytic subunit	Nucleus	Apoptosis
			Developmental processes
			Protein metabolism and modification
			Nucleoside, nucleotide and nucleic acid metabolism
			Carbohydrate metabolism
			Cell structure and mobility
P40199	Carcinoembryonic antigen-related cell adhesion molecule 6	Membrane Fraction	Cell adhesion

Accession No	Protein Name	Cellular Localisation	Functional Terms
P40925	Malate dehydrogenase, cytoplasmic	Cytoplasm	Carbohydrate metabolism
P41219	Peripherin	Cytoplasm	Cell structure and mobility
P43686	26S protease regulatory subunit 6B	Mitochondrion	Protein metabolism and modification
P45880	Voltage-dependent anion-selective channel protein 2	Mitochondrion	Transport
P46063	ATP-dependent DNA helicase Q1	Nucleus	Nucleoside, nucleotide and nucleic acid metabolism
P46779	60S ribosomal protein L28	Cytoplasm	Cell cycle
P46781	40S ribosomal protein S9	Cytoplasm	Protein metabolism and modification
P46782	40S ribosomal protein S5	Cytoplasm	Protein metabolism and modification
P46783	40S ribosomal protein S10	Cytoplasm	Protein metabolism and modification
P47755	F-actin-capping protein subunit alpha-2	Cytoplasm	Cell structure and mobility
P47897	Glutaminyl-tRNA synthetase	Soluble Fraction	Protein metabolism and modification
P48163	NADP-dependent malic enzyme	Nucleus	Carbohydrate metabolism<br>Amino acid metabolism<br>Other metabolism
P48643	T-complex protein 1 subunit epsilon	Nucleus	Protein metabolism and modification
P49207	60S ribosomal protein L34	Cytoplasm	Protein metabolism and modification
P49720	Proteasome subunit beta type-3	Nucleus	Protein metabolism and modification
P49902	Cytosolic purine 5'-nucleotidase	Cytoplasm	Nucleoside, nucleotide and nucleic acid metabolism
P50213	Isocitrate dehydrogenase [NAD] subunit alpha, mitochondrial	Mitochondrion	Electron transport
P50238	Cysteine-rich protein 1	Cytoplasm	Developmental processes
P50395	Rab GDP dissociation inhibitor beta	Cell Surface	Intracellular protein traffic<br>Transport<br>Signal transduction
P50552	Vasodilator-stimulated phosphoprotein	Cytoplasm	Cell structure and mobility
P50990	T-complex protein 1 subunit theta	Cytoplasm	Protein metabolism and modification
P51665	26S proteasome non-ATPase regulatory subunit 7	Intracellular	Protein metabolism and modification<br>Developmental processes<br>Cell cycle<br>Cell proliferation and differentiation
P53007	Tricarboxylate transport protein, mitochondrial	Mitochondrion	Transport

Accession No	Protein Name	Cellular Localisation	Functional Terms
P53396	ATP-citrate synthase	Cytoplasm	Lipid, fatty acid and steroid metabolism
			Coenzyme and prosthetic group metabolism
			Carbohydrate metabolism
P53634	Dipeptidyl-peptidase 1	Lysosome	Protein metabolism and modification
P55957	BH3-interacting domain death agonist	Mitochondrion	Signal transduction
			Apoptosis
			Developmental processes
P56211	cAMP-regulated phosphoprotein 19	Intracellular	Protein metabolism and modification
P56385	ATP synthase subunit e, mitochondrial	Mitochondrion	Nucleoside, nucleotide and nucleic acid metabolism
P58876	Histone H2B type 1-D	ND	ND
P59998	Actin-related protein 2/3 complex subunit 4	Cytoskeleton	Cell structure and mobility
P60660	Myosin light polypeptide 6	Cytoskeleton	Muscle contraction
P61019	Ras-related protein Rab-2A	Membrane	Signal transduction
			Intracellular protein traffic
P61026	Ras-related protein Rab-10	Golgi Apparatus	Intracellular protein traffic
P61088	Ubiquitin-conjugating enzyme E2 N	Nucleus	Protein metabolism and modification
P61158	Actin-related protein 3	Cytoskeleton	Intracellular protein traffic
			Transport
			Cell cycle
			Cell structure and mobility
P61204	ADP-ribosylation factor 3	Golgi Apparatus	Intracellular protein traffic
P61247	40S ribosomal protein S3a	Cytoplasm	Protein metabolism and modification
P61313	60S ribosomal protein L15	Cytoplasm	Protein metabolism and modification
P61353	60S ribosomal protein L27	Cytoplasm	Signal transduction
			Protein targeting and localization
			Cell cycle
P61604	10 kDa heat shock protein, mitochondrial	Mitochondrion	Protein metabolism and modification
P62241	40S ribosomal protein S8	Cytoplasm	Protein metabolism and modification
P62258	14-3-3 protein epsilon	Cytoplasm	Protein metabolism and modification
P62277	40S ribosomal protein S13	Nucleus	Protein metabolism and modification

Accession No	Protein Name	Cellular Localisation	Functional Terms
P62701	40S ribosomal protein S4, X isoform	Cytoplasm	Protein metabolism and modification
P62750	60S ribosomal protein L23a	Cytoplasm	Protein metabolism and modification
P62826	GTP-binding nuclear protein Ran	Nucleus	Cell cycle
			Intracellular protein traffic
			Nucleoside, nucleotide and nucleic acid metabolism
			Signal transduction
P62841	40S ribosomal protein S15	Cytoplasm	Protein metabolism and modification
P62857	40S ribosomal protein S28	Cytoplasm	Protein metabolism and modification
P62942	Peptidyl-prolyl cis-trans isomerase FKBP1A	Cytoplasm	Muscle contraction
P62987	60S ribosomal protein L40	Cytoplasm	ND
P63000	Ras-related C3 botulinum toxin substrate 1	Cytoplasm	Signal transduction
			Cell structure and mobility
P63244	Guanine nucleotide-binding protein subunit beta-2-like 1	Cytoplasm	Signal transduction
			Protein targeting and localization
P67809	Nuclease-sensitive element-binding protein 1	Cytoplasm	Nucleoside, nucleotide and nucleic acid metabolism
P68371	Tubulin beta-2C chain	Cytoskeleton	Intracellular protein traffic
			Cell cycle
			Cell structure and mobility
P78329	Cytochrome P450 4F2	Cytoplasm	Lipid, fatty acid and steroid metabolism
			Electron transport
P80748	Ig lambda chain V-III region LOI	Extracellular Region	Immunity and defense
P84098	60S ribosomal protein L19	Cytoplasm	Nucleoside, nucleotide and nucleic acid metabolism
Q01130	Splicing factor, arginine/serine-rich 2	Nucleus	Nucleoside, nucleotide and nucleic acid metabolism
Q02543	60S ribosomal protein L18a	Cytoplasm	Protein metabolism and modification
Q04760	Lactoylglutathione lyase	Cytoplasm	Other metabolism
Q07020	60S ribosomal protein L18	Cytoplasm	Protein metabolism and modification
Q07955	Splicing factor, arginine/serine-rich 1	Nucleus	Nucleoside, nucleotide and nucleic acid metabolism
Q08AI8	Uncharacterized protein C2orf54	ND	ND
Q12792	Twinfilin-1	Cytoskeleton	Protein metabolism and modification
Q13404	Ubiquitin-conjugating enzyme E2 variant 1	Nucleus	Protein metabolism and modification

Accession No	Protein Name	Cellular Localisation	Functional Terms
Q14002	Carcinoembryonic antigen-related cell adhesion molecule 7	Membrane Fraction	Cell adhesion
Q14247	Src substrate cortactin	Cytoskeleton	Cell structure and mobility
Q14687	Genetic suppressor element 1	ND	ND
Q14790	Caspase-8	Cytoskeleton	Apoptosis
			Protein metabolism and modification
Q14847	LIM and SH3 domain protein 1	Cytoskeleton	Muscle contraction
Q14CN2	Calcium-activated chloride channel regulator 4	Membrane	Transport
Q15181	Inorganic pyrophosphatase	Cytoplasm	Other metabolism
Q15369	Transcription elongation factor B polypeptide 1	Nucleus	Nucleoside, nucleotide and nucleic acid metabolism
Q15691	Microtubule-associated protein RP/EB family member 1	Cytoskeleton	Cell cycle
			Cell proliferation and differentiation
			Cell structure and mobility
Q15849	Urea transporter, kidney	Membrane Fraction	Intracellular protein traffic
Q16719	Kynureninase	Mitochondrion	Amino acid metabolism
Q16762	Thiosulfate sulfurtransferase	Mitochondrion	Electron transport
Q16778	Histone H2B type 2-E	Nucleus	Nucleoside, nucleotide and nucleic acid metabolism
Q16851	UTP--glucose-1-phosphate uridylyltransferase	Cytoplasm	Nucleoside, nucleotide and nucleic acid metabolism
Q32NC0	UPF0711 protein C18orf21	ND	ND
Q53F39	Metallophosphoesterase 1	Membrane Fraction	ND
Q5JNZ5	Putative 40S ribosomal protein S26-like 1	ND	ND
Q5QNW6	Histone H2B type 2-F	Nucleus	Nucleoside, nucleotide and nucleic acid metabolism
Q5SSG8	Mucin-21	Membrane Fraction	ND
Q5SWL7	PRAME family member 14	ND	ND
Q5T013	Putative hydroxypyruvate isomerase	Cytoplasm	Nucleoside, nucleotide and nucleic acid metabolism
Q5T749	Keratinocyte proline-rich protein	Cytoplasm	ND
Q6S8J3	ANKRD26-like family C member 1A	Intracellular	ND
Q6UX06	Olfactomedin-4	Extracellular Region	Oncogenesis
Q6WKZ4	Rab11 family-interacting protein 1	Endosome	Protein targeting and localization
			Transport
Q6XQN6	Nicotinate phosphoribosyltransferase	Cytoplasm	ND

Accession No	Protein Name	Cellular Localisation	Functional Terms
Q6ZT12	E3 ubiquitin-protein ligase UBR3	Cytoplasm	ND
Q7Z589	Protein EMSY	Nucleus	Nucleoside, nucleotide and nucleic acid metabolism
Q7Z7A1	Centriolin	ND	ND
Q86V81	THO complex subunit 4	Nucleus	Nucleoside, nucleotide and nucleic acid metabolism
Q86YQ8	Copine-8	Membrane	Transport
			Miscellaneous
Q8IUD2	ELKS/RAB6-interacting/CAST family member 1	Membrane	Intracellular protein traffic
Q8NCW5	Apolipoprotein A-I-binding protein	Extracellular Region	ND
Q8TD20	Solute carrier family 2, facilitated glucose transporter member 12	Membrane	Carbohydrate metabolism
			Transport
Q8WUF5	RelA-associated inhibitor	Nucleus	Apoptosis
			Cell cycle
			Cell proliferation and differentiation
Q8WUM4	Programmed cell death 6-interacting protein	Cytoplasm	Apoptosis
			Cell proliferation and differentiation
Q92522	Histone H1x	Nucleus	Nucleoside, nucleotide and nucleic acid metabolism
Q92985	Interferon regulatory factor 7	Nucleus	Immunity and defense
			Oncogenesis
Q93070	Ecto-ADP-ribosyltransferase 4	Membrane Fraction	Other metabolism
Q96AE4	Far upstream element-binding protein 1	Nucleus	ND
Q96E52	Metalloendopeptidase OMA1, mitochondrial	Mitochondrion	Protein metabolism and modification
Q96GM5	SWI/SNF-related matrix-associated actin-dependent regulator of chromatin subfamily D member 1	Nucleus	Nucleoside, nucleotide and nucleic acid metabolism
Q96KP4	Cytosolic non-specific dipeptidase	Cytoplasm	Lipid, fatty acid and steroid metabolism
Q96L46	Calpain small subunit 2	Membrane	Protein metabolism and modification
			Signal transduction
Q96S53	Dual specificity testis-specific protein kinase 2	Nucleus	Protein metabolism and modification
			Signal transduction
			Cell structure and mobility
Q99615	DnaJ homolog subfamily C member 7	ND	Protein metabolism and modification

Accession No	Protein Name	Cellular Localisation	Functional Terms
Q99729	Heterogeneous nuclear ribonucleoprotein A/B	Nucleus	Nucleoside, nucleotide and nucleic acid metabolism
Q99816	Tumor susceptibility gene 101 protein	Endosome	Nucleoside, nucleotide and nucleic acid metabolism
			Cell cycle
			Cell proliferation and differentiation
Q99832	T-complex protein 1 subunit eta	Cytoplasm	Protein metabolism and modification
Q99878	Histone H2A type 1-J	Nucleus	Nucleoside, nucleotide and nucleic acid metabolism
Q99879	Histone H2B type 1-M	Intracellular	Other metabolism
Q9BPX5	Actin-related protein 2/3 complex subunit 5-like protein	Cytoskeleton	Cell adhesion
Q9BQI0	Allograft inflammatory factor 1-like	Cytoskeleton	Immunity and defense
			Cell cycle
			Cell proliferation and differentiation
Q9BRG1	Vacuolar protein-sorting-associated protein 25	Nucleus	Nucleoside, nucleotide and nucleic acid metabolism
			Other metabolism
			Transport
			Protein metabolism and modification
			Intracellular protein traffic
			Protein targeting and localization
Q9BRL6	Splicing factor, arginine/serine-rich 2B	Nucleus	Nucleoside, nucleotide and nucleic acid metabolism
Q9BTM1	Histone H2A.J	Nucleus	Nucleoside, nucleotide and nucleic acid metabolism
Q9BVA1	Tubulin beta-2B chain	Cytoskeleton	Intracellular protein traffic
			Cell cycle
			Cell structure and mobility
Q9BVK6	Transmembrane emp24 domain-containing protein 9	Cytoplasm	Intracellular protein traffic
Q9BWD1	Acetyl-CoA acetyltransferase, cytosolic	Cytoplasm	Protein metabolism and modification
Q9BYE2	Transmembrane protease, serine 13	Membrane	Protein metabolism and modification
Q9H299	SH3 domain-binding glutamic acid-rich-like protein 3	Nucleus	Homeostasis
Q9H444	Charged multivesicular body protein 4b	Endosome	Protein targeting and localization
Q9H910	Hematological and neurological expressed 1-like protein	Intracellular	ND
Q9HC10	Otoferlin	Membrane Fraction	ND
Q9HCY8	S100A14	Cytoplasm	ND

Accession No	Protein Name	Cellular Localisation	Functional Terms
Q9NP79	Vacuolar protein sorting-associated protein VTA1 homolog	Endosome	Protein targeting and localization
Q9NQ39	Putative 40S ribosomal protein S10-like protein	Endoplasmic Reticulum	ND
Q9NQC3	Reticulon-4	Endoplasmic Reticulum	Neuronal activities
Q9NSA3	Beta-catenin-interacting protein 1	Nucleus	Signal transduction
			Nucleoside, nucleotide and nucleic acid metabolism
Q9NTK5	Obg-like ATPase 1	Cytoplasm	Nucleoside, nucleotide and nucleic acid metabolism
Q9NYL9	Tropomodulin-3	Cytoskeleton	Cell structure and mobility
Q9NZN3	EH domain-containing protein 3	Nucleus	Intracellular protein traffic
			Neuronal activities
Q9P0L0	Vesicle-associated membrane protein-associated protein A	Membrane Fraction	Protein targeting and localization
Q9P1U1	Actin-related protein 3B	Cytoplasm	Cell structure and mobility
Q9P2E9	Ribosome-binding protein 1	Endoplasmic Reticulum	Miscellaneous
Q9UBL6	Copine-7	Membrane Fraction	Intracellular protein traffic
Q9UHA7	Interleukin-1 family member 6	Extracellular Region	Immunity and defense
			Signal transduction
Q9UHD0	Interleukin-19	Extracellular Region	Immunity and defense
Q9UJU6	Drebrin-like protein	Cytoplasm	Cell structure and mobility
Q9UJV9	Probable ATP-dependent RNA helicase DDX41	Nucleus	Nucleoside, nucleotide and nucleic acid metabolism
Q9UKX3	Myosin-13	Cytoplasm	Muscle contraction
Q9UL25	Ras-related protein Rab-21	Golgi Apparatus	Signal transduction
			Intracellular protein traffic
Q9UNX3	60S ribosomal protein L26-like 1	Intracellular	Protein metabolism and modification
Q9Y281	Cofilin-2	Cytoskeleton	Cell structure and mobility
Q9Y376	Calcium-binding protein 39	Cytoplasm	Other metabolism
Q9Y3U8	60S ribosomal protein L36	Cytoplasm	Intracellular protein traffic
Q9Y5Z4	Heme-binding protein 2	Cytoplasm	Blood circulation and gas exchange
A8K5I0	Heat shock 70 kDa protein 1B*	Mitochondrion	Apoptosis
			Nucleoside, nucleotide and nucleic acid metabolism
O00468	Agrin precursor*	Extracellular Region	Signal transduction
			Other metabolism

Accession No	Protein Name	Cellular Localisation	Functional Terms
			Cell proliferation and differentiation
O00592	Podocalyxin-like precursor isoform 1*	Membrane	Cell adhesion
			Cell structure and mobility
O43490	Prominin 1*	Membrane	Neuronal activities
O75339	Cartilage intermediate layer protein*	Extracellular Region	Signal transduction
O75556	Secretoglobin family 2A, member 1*	ND	ND
O95994	Anterior gradient protein 2 homolog precursor*	Extracellular Region	Developmental processes
P00746	Complement factor D precursor*	Extracellular Region	Immunity and defense
			Protein metabolism and modification
P00797	Renin*	Cytoplasm	Signal transduction
			Cell proliferation and differentiation
			Protein metabolism and modification
P02730	Solute carrier family 4,anion exchanger,member 15*	Cytoskeleton	Transport
P04350	Tubulin beta-4 chain*	Cytoskeleton	Cell structure and mobility
P04745	Alpha-amylase*	ND	Carbohydrate metabolism
P05114	Non-histone chromosomal protein HMG-14*	Nucleus	Nucleoside, nucleotide and nucleic acid metabolism
P06314	Ig kappa light chain VLJ region*	ND	ND
P07437	Tubulin, beta polypeptide*	Cytoskeleton	Cell structure and mobility
P0AA25	IgE Fv SPE7 chain B complexed with a recomb.thioredoxin*	ND	Carbohydrate metabolism
P10253	Alpha-glucosidase*	Cytoplasm	Carbohydrate metabolism
P11499	Heat shock protein HSP 90-beta*	Mitochondrion	Protein metabolism and modification
P12821	Angiotensin I converting enzyme isoform 1*	Extracellular Region	Protein metabolism and modification
			Transport
P14550	Alcohol dehydrogenase [NADP+]*	Membrane	Nucleoside, nucleotide and nucleic acid metabolism
P15144	Membrane alanine aminopeptidase precursor*	Golgi Apparatus	Protein metabolism and modification
			Cell proliferation and differentiation
P17066	Heat shock 70 kDa protein 6*	Cytoplasm	Immunity and defense
			Protein metabolism and modification
P19021	Peptidylglycine alpha-amidating monooxygenase*	Membrane	Protein metabolism and modification
P19120	Heat shock cognate 71 kDa protein*	Cytoplasm	Protein metabolism and modification

Accession No	Protein Name	Cellular Localisation	Functional Terms
P19440	Gamma-glutamyl transpeptidase*	Membrane	Protein metabolism and modification
			Amino acid metabolism
P19827	Inter-alpha (globulin) inhibitor H1*	ND	Carbohydrate metabolism
P21589	5' nucleotidase,ecto*	Membrane	Nucleoside, nucleotide and nucleic acid metabolism
			Immunity and defense
P22352	Plasma glutathione peroxidase*	Extracellular Region	Protein metabolism and modification
P24821	Tenascin C*	Extracellular Region	Developmental processes
			Immunity and defense
			Signal transduction
P27169	Serum paroxonase 1*	Membrane Fraction	Amino acid metabolism
P36222	Chitinase 3-like 1*	Extracellular Region	Carbohydrate metabolism
P47710	Casein alphaS1*	ND	Transport
P49061	Metalloproteinase inhibitor 1 precursor*	Extracellular Region	Developmental processes
			Cell proliferation and differentiation
			Homeostasis
P52823	Stanniocalcin 1 precursor*	ND	Signal transduction
P63102	14-3-3 zeta isoform*	Cytoplasm	Immunity and defense
			Transport
P68363	Tubulin alpha-1B chain*	Cytoskeleton	Cell structure and mobility
Q12889	Oviductal glycoprotein*	ND	Carbohydrate metabolism
Q13421	Mesothelin precursor *	Golgi Apparatus	Signal transduction
			Cell adhesion
			Developmental processes
Q13938	Calcyphosine isoform a*	Cytoplasm	Signal transduction
Q15768	Ephrin B3*	Membrane	Cell structure and mobility
Q16651	Prostasin preproprotein*	Membrane	Protein metabolism and modification
Q4VJB6	14-3-3 protein epsilon isoform transcript variant 1*	Mitochondrion	Cell proliferation and differentiation
			Intracellular protein traffic
			Transport
			Signal transduction

Accession No	Protein Name	Cellular Localisation	Functional Terms
Q53G64	Anterior gradient 2 homolog*	Endoplasmic Reticulum	Transport
Q5JNX2	Complement component 4a*	Extracellular Region	Immunity and defense
Q68CK4	Leucine-rich alpha-2-glycoprotein 1*	Nucleus	Cell proliferation and differentiation
Q6NUR7	Villin 23*	Membrane	Cell structure and mobility
Q6PJF2	IgM, Fab fragment of cold agglutinin chain C*	ND	ND
Q86WI1	Fibrocystin L*	Membrane	ND
Q92520	Predicted osteoblast protein*	ND	ND
Q92743	HtrA serine peptidase 1*	Extracellular Region	Protein metabolism and modification
			Developmental processes
Q99536	Vesicle amine transport protein 1*	Nucleus	Cell structure and mobility
